# Ontogeny in the tube-crested dinosaur *Parasaurolophus* (Hadrosauridae) and heterochrony in hadrosaurids

**DOI:** 10.7717/peerj.182

**Published:** 2013-10-22

**Authors:** Andrew A. Farke, Derek J. Chok, Annisa Herrero, Brandon Scolieri, Sarah Werning

**Affiliations:** 1 Raymond M. Alf Museum of Paleontology, Claremont, CA, USA; 2 The Webb Schools, Claremont, CA, USA; 3 Department of Integrative Biology, Museum of Paleontology, and Museum of Vertebrate Zoology, University of California, Berkeley, CA, USA

**Keywords:** *Parasaurolophus*, Ontogeny, Hadrosauridae, Kaiparowits Formation, Cretaceous, Dinosauria, Lambeosaurinae, Ornithischia, Heterochrony

## Abstract

The tube-crested hadrosaurid dinosaur *Parasaurolophus* is remarkable for its unusual cranial ornamentation, but little is known about its growth and development, particularly relative to well-documented ontogenetic series for lambeosaurin hadrosaurids (such as *Corythosaurus*, *Lambeosaurus*, and *Hypacrosaurus*). The skull and skeleton of a juvenile *Parasaurolophus* from the late Campanian-aged (∼75.5 Ma) Kaiparowits Formation of southern Utah, USA, represents the smallest and most complete specimen yet described for this taxon. The individual was approximately 2.5 m in body length (∼25% maximum adult body length) at death, with a skull measuring 246 mm long and a femur 329 mm long. A histological section of the tibia shows well-vascularized, woven and parallel-fibered primary cortical bone typical of juvenile ornithopods. The histological section revealed no lines of arrested growth or annuli, suggesting the animal may have still been in its first year at the time of death. Impressions of the upper rhamphotheca are preserved in association with the skull, showing that the soft tissue component for the beak extended for some distance beyond the limits of the oral margin of the premaxilla. In marked contrast with the lengthy tube-like crest in adult *Parasaurolophus*, the crest of the juvenile specimen is low and hemicircular in profile, with an open premaxilla-nasal fontanelle. Unlike juvenile lambeosaurins, the nasal passages occupy nearly the entirety of the crest in juvenile *Parasaurolophus*. Furthermore, *Parasaurolophus* initiated development of the crest at less than 25% maximum skull size, contrasting with 50% of maximum skull size in hadrosaurs such as *Corythosaurus*. This early development may correspond with the larger and more derived form of the crest in *Parasaurolophus*, as well as the close relationship between the crest and the respiratory system. In general, ornithischian dinosaurs formed bony cranial ornamentation at a relatively younger age and smaller size than seen in extant birds. This may reflect, at least in part, that ornithischians probably reached sexual maturity prior to somatic maturity, whereas birds become reproductively mature after reaching adult size.

## Introduction

Ontogenetic changes in the vertebrate skull have numerous functional, ecological, and behavioral consequences (e.g., Erickson, Lappin & Vliet, 2003; Herrel & Gibb, 2006; Herrel & O’Reilly, 2006; Cole, 2010). Variation in the timing and degree of development of these changes relative to the ancestral condition (heterochrony; e.g., Gould, 1977; Alberch et al., 1979; Klingenberg, 1998; Smith, 2001) is responsible, in part, for the diversity seen even among closely related species. The ontogeny of the skull in ornithischian dinosaurs has received particular attention, due to their elaborate horns, crests, casques and domes in a number of species, variously interpreted to function in visual display, sound production, and intraspecific combat (see Hone, Naish & Cuthill, 2012 for a recent summary). These cranial modifications demonstrate considerable variation in their morphology as well as heterochrony in their appearance and modification. For instance, the dome-headed pachycephalosaurs show early development of peripheral spikes and knobs and late development of an enlarged central dome (Horner & Goodwin, 2009; Schott et al., 2011; Schott & Evans, 2012), whereas the horned dinosaurs (ceratopsians) have early and continuous development of horns and frills with a final burst of extreme modification to the horns and marginal bones of the frill late in ontogeny (Dodson, 1976; Sampson, Ryan & Tanke, 1997; Horner & Goodwin, 2006; Currie, Langston & Tanke, 2008). These developmental patterns have been leveraged to better inform speculation on cranial function in each of these groups.

Among the hadrosaurids, or duck-billed dinosaurs, lambeosaurines are remarkable for their heavily modified nasal passages within a bony crest. Various functional hypotheses have been proposed for this anatomical complex, including air storage during underwater feeding, enhanced olfaction, housing for a salt gland, vocal resonance chambers, and visual display for mate attraction and/or species recognition (reviewed in Weishampel, 1981a). Currently, vocalization and visual display together are the most broadly accepted hypotheses (Evans, 2006), based in part on ontogenetic patterns for the crests. These structures are not well-manifested externally until the skull reaches approximately 50 percent of maximum adult size, and then apparently grew continuously and with strong positive allometry relative to the rest of the skull (Dodson, 1976; Evans, 2010). These patterns of cranial ontogeny are best documented in Lambeosaurini, the clade of “helmet-crested” lambeosaurines that includes taxa such as *Corythosaurus*, *Lambeosaurus*, and *Hypacrosaurus* (Dodson, 1975; Horner & Currie, 1994; Evans, Forster & Reisz, 2005; Evans, Ridgely & Witmer, 2009; Evans, 2010; Bailleul, Hall & Horner, 2012). Data from a number of well-preserved specimens representing individuals of various sizes and ontogenetic stages allow detailed comparisons of growth and anatomy in closely related species. Importantly, results show that some diagnostic anatomical features arise early in development (e.g., the lack of a premaxilla-nasal fontanelle in *Hypacrosaurus altispinus*), whereas others (e.g., the distinct hatchet-shaped crest of *Lambeosaurus lambei*) arise later (Evans, 2010). In any case, the final adult profile is not completed until late in ontogeny, when the animals reach nearly full adult skull size. Although these data have been critical in defining models of lambeosaurine ontogeny, the narrow taxonomic sampling limits application of these models across the clade.

In gross view, the cranial crests of lambeosaurins are fairly uniform, dominated by a hemicircular profile sometimes augmented with a caudally projecting spike. This contrasts with the condition in Parasaurolophini, the other major clade of lambeosaurines that includes *Parasaurolophus* and *Charonosaurus*. Parasaurolophins are notable for their greatly elongated, tubular crests that project caudally from the skull. The differences between adult parasaurolophins and lambeosaurins almost certainly reflect different ontogenetic trajectories, but the ontogeny of the skull in general and the crest in particular is poorly known in parasaurolophins. Sullivan & Bennett (2000) referred an incomplete and disarticulated skull from New Mexico to *Parasaurolophus*, but this specimen (approximately one-third the size of an adult) did not include any portion of the skull roof except for a possible postorbital. Evans, Reisz & Dupuis (2007) referred a braincase from Alberta to *Parasaurolophus*, from an individual approximately half of adult size. Although the crest itself was not preserved, the frontal platform that supported the crest was well-developed (in contrast with the poorly developed platform of lambeosaurins at all ontogenetic stages), implying that the tubular crest was already at least partially developed in that individual. This limited evidence suggests fundamental differences between the cranial development of parasaurolophins and lambeosaurins.

Heterochrony in hadrosaurid dinosaurs has received limited attention to date, perhaps in part due to the absence of multiple comprehensive growth series for this clade. One of the first treatments (Weishampel & Horner, 1994) focused primarily on the interplay between body size and age, positing a reduction in skeletal maturity at hatching. Along with the retention of small teeth into adulthood (Weishampel, Norman & Grigorescu, 1993), this would suggest paedomorphosis (prolonged retention of juvenile characters through development relative to the ancestral condition (Alberch et al., 1979)) as a factor in development of these structures. Peramorphosis—acceleration and/or exaggeration of growth in certain features relative to the ancestral condition (Alberch et al., 1979)—was implicated in the development of cranial ornamentation and the oral margins in many hadrosaurids (Long & McNamara, 1997). Additional work on the postcranial skeleton showed heterochrony in some aspects of its ontogeny, such as peramorphosis of the supraacetabular process in the ilium of *Hypacrosaurus* relative to other hadrosaurids (Guenther, 2009, within the conceptual framework of sequence heterochrony). Overall, heterochrony in the evolution of lambeosaurine cranial ornamentation has received little detailed evaluation.

**Figure 1:**
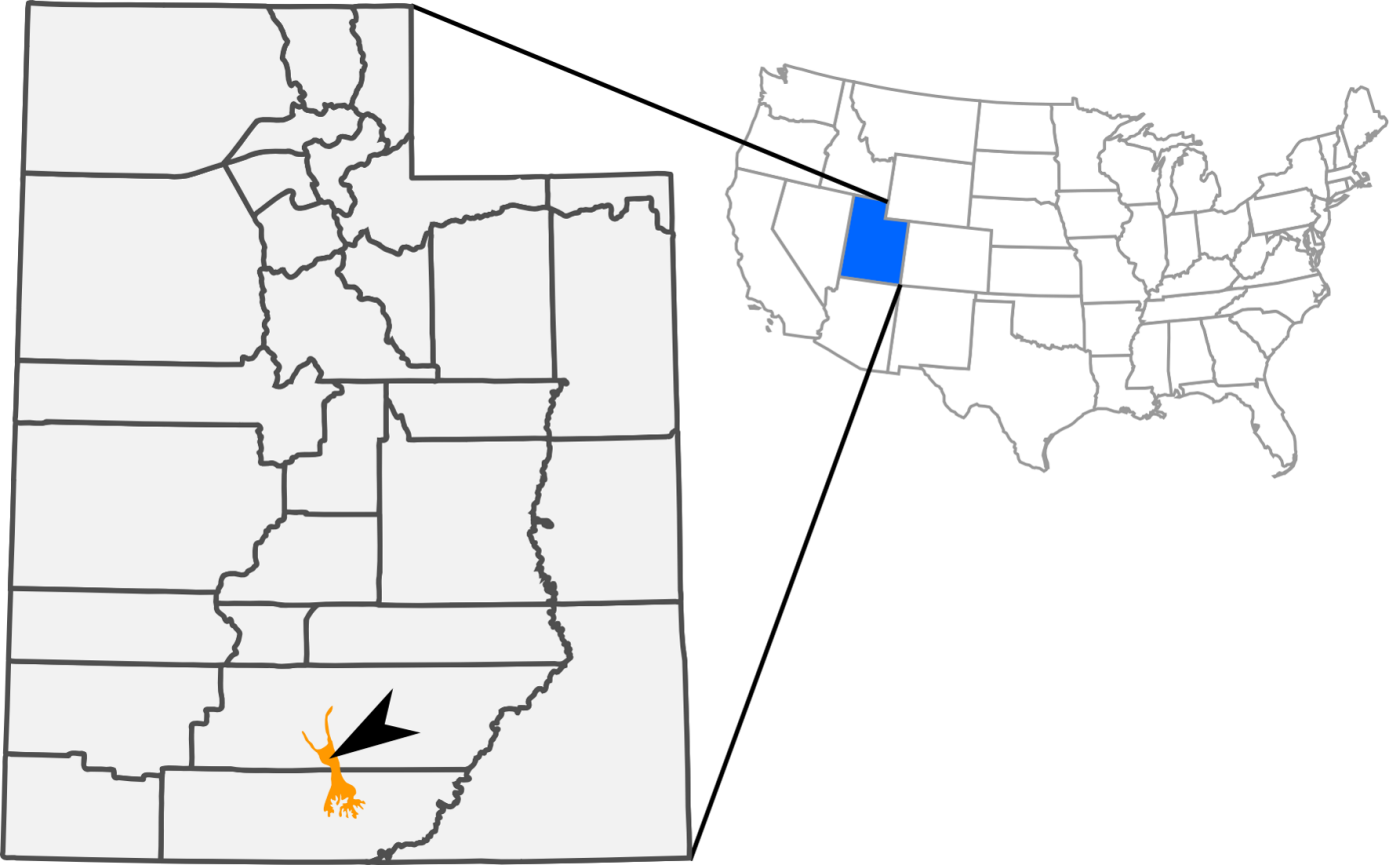
Outcrops of Kaiparowits Formation (orange) within the state of Utah, USA. The arrow indicates the approximate site of RAM V200921, the locality where RAM 14000 was discovered.

During the 2009 joint field season for The Webb Schools and the Raymond M. Alf Museum of Paleontology (RAM, Claremont, California, USA), high school student Kevin Terris discovered the articulated skeleton and skull of a small hadrosaurid dinosaur (total body length ∼2.5 m). The specimen originated in the late Campanian (∼75.5 million years old) Kaiparowits Formation, exposed within Grand Staircase-Escalante National Monument in southern Utah (Fig. 1; Roberts, Deino & Chan, 2005; Roberts, 2007). This fossil (RAM 14000) is here referred to *Parasaurolophus*, representing the ontogenetically youngest and most complete specimen ever recovered for the genus.

**Figure 2:**
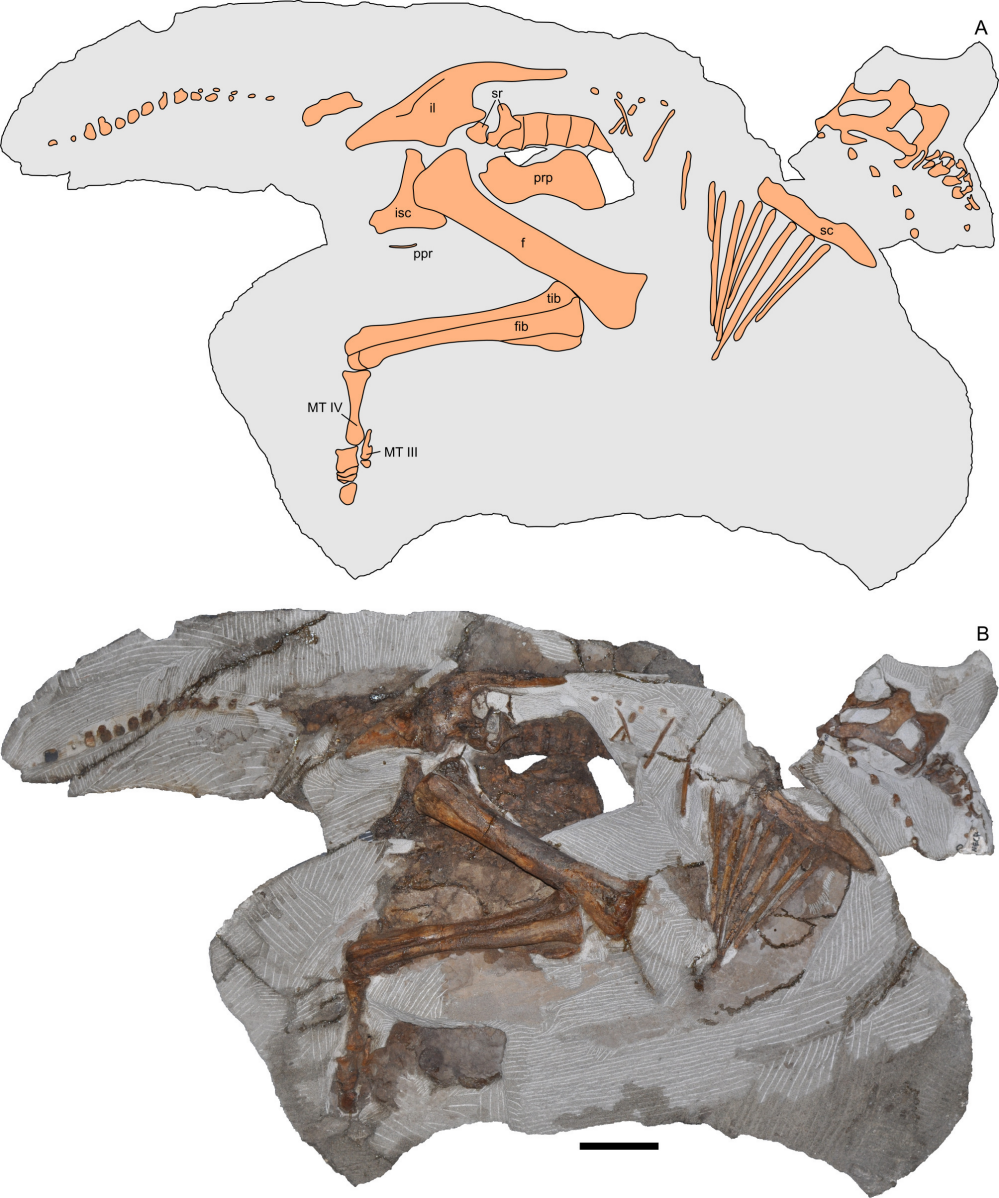
Skeleton of *Parasaurolophus* sp., RAM 14000, in right lateral view. (A) interpretive drawing; (B) photograph. Bones are bounded by solid lines and colored orange; matrix is gray. Abbreviations: f, femur; fib, fibula; il, ilium; isc, ischium; MT III, metatarsal III; MT IV, metatarsal IV; ppr, postpubic rod; prp, prepubic process; sc, scapula; sr, sacral rib; tib, tibia. Scale bar equals 10 cm.

**Figure 3:**
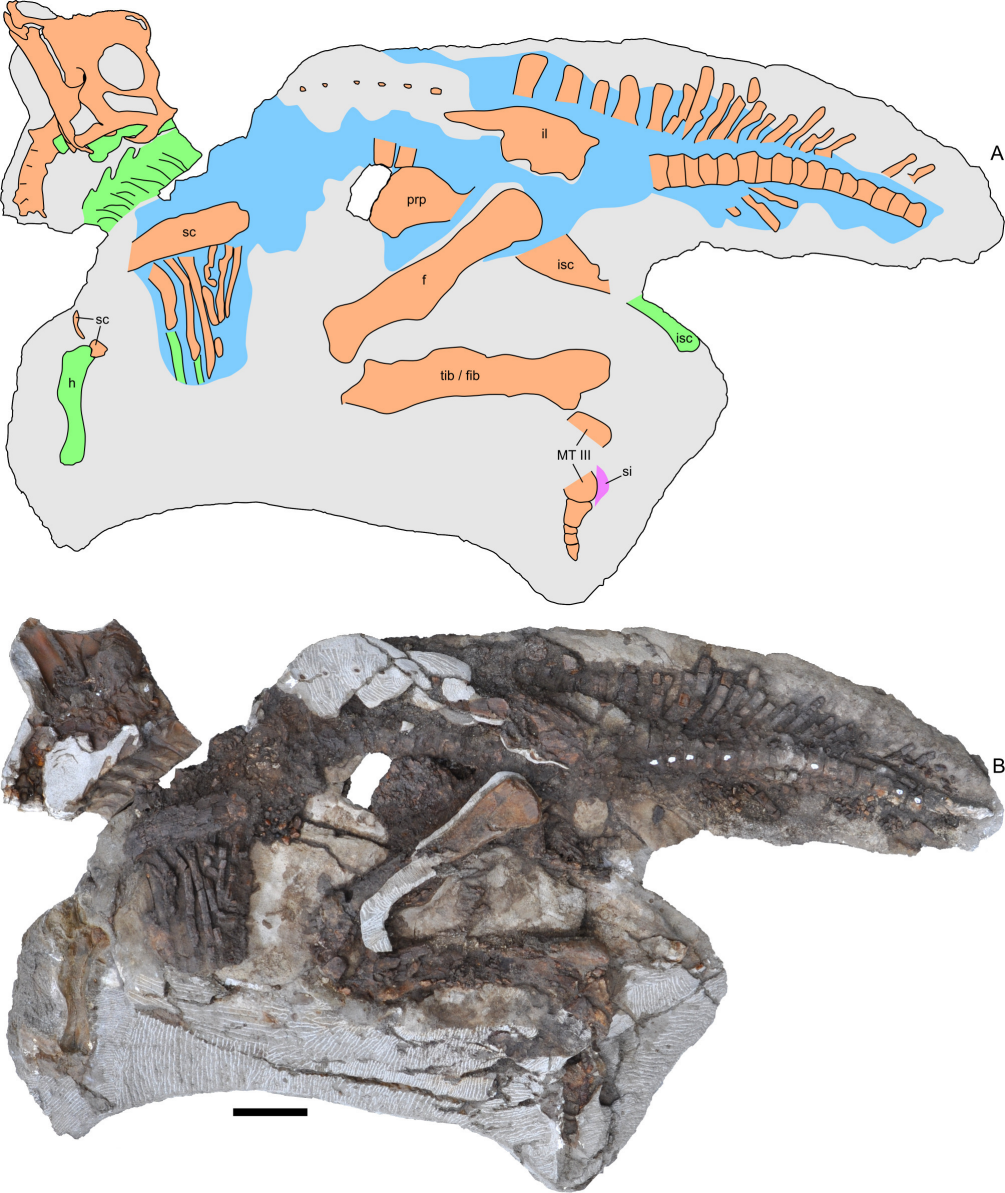
Skeleton of *Parasaurolophus* sp., RAM 14000, in left lateral view. (A) interpretive drawing; (B) photograph. Bones are bounded by solid lines and colored orange; blue indicates areas of fragmented and powdered bone due to weathering, and green indicates bone impressions. The pink area indicates the location of skin impressions shown in Fig. 21. In (A), the left half of the skull is indicated. A detailed outline of the medial surface of the right half of the skull shown in (B) is contained in Fig. 13B. Abbreviations: f, femur; fib, fibula; h, humerus; il, ilium; isc, ischium; MT III, metatarsal III; prp, prepubic process; sc, scapula; si, skin impression; tib, tibia. Scale bar equals 10 cm.

**Figure 4:**
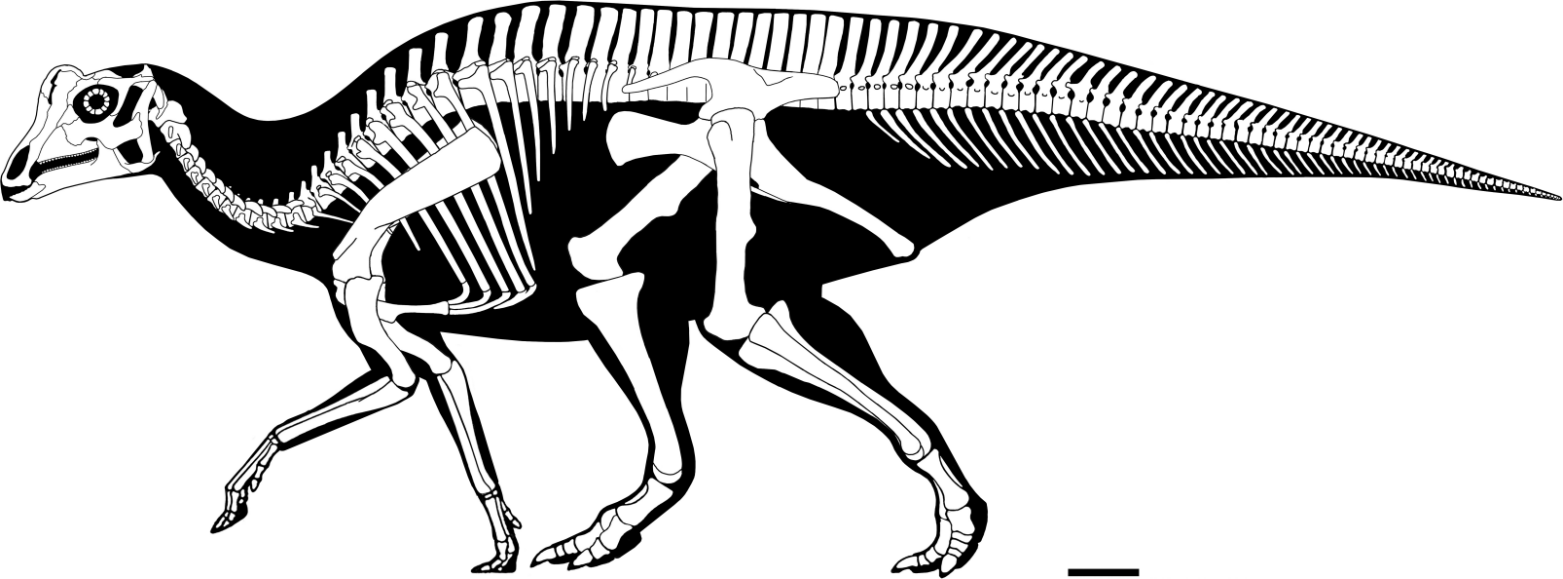
Reconstructed skeleton of juvenile *Parasaurolophus* sp., in left lateral view, based on RAM 14000. Missing elements are patterned after other lambeosaurines (particularly a juvenile *Lambeosaurus* sp., AMNH 5340). Scale bar equals 10 cm. Reconstruction courtesy of and copyright Scott Hartman.

The nearly complete skull, articulated postcranial skeleton, and associated soft-tissue in RAM 14000 (Figs. 2–4) provide important new data on anatomy and ontogeny in *Parasaurolophus* and hadrosaurids in general. Here, we present a comprehensive description of RAM 14000, placing it within the broader context of ontogeny and heterochrony in lambeosaurines and other dinosaurs. Critically, the specimen provides the best record to date of an early ontogenetic stage in a parasaurolophin, clearly elucidating previously suspected differences between the ontogeny in this clade and in lambeosaurins. Furthermore, the specimen provides a starting point for a broader discussion of heterochrony and “odd” cranial structures in dinosaurs.

### Institutional abbreviations

AMNH, American Museum of Natural History, New York, New York, USA; BYU, Brigham Young University, Provo, Utah, USA; CMN, Canadian Museum of Nature, Ottawa, Ontario, Canada; CPC, Colección Paleontológica de Coahuila, Museo del Desierto, Saltillo, Coahuila, México; NMMNH, New Mexico Museum of Natural History, Albuquerque, New Mexico, USA; OUVC, Ohio University Veterinary Collection, Athens, Ohio, USA; PIN, Paleontological Institute, Russian Academy of Sciences, Moscow, Russia; PMU, Museum of Evolution, Uppsala University, Uppsala, Sweden; RAM, Raymond M. Alf Museum of Paleontology, Claremont, California, USA; ROM, Royal Ontario Museum, Toronto, Ontario, Canada; SMP, State Museum of Pennsylvania, Harrisburg, Pennsylvania, USA; TMP, Royal Tyrrell Museum of Paleontology, Drumheller, Alberta, Canada; UCMP, University of California Museum of Paleontology, Berkeley, California, USA; UMNH, Natural History Museum of Utah, Salt Lake City, Utah, USA.

## Methods

### Fieldwork and preparation

All fieldwork was conducted under United States Department of the Interior Bureau of Land Management Paleontological Resources Use Permit (surface collection permit UT06-001S and excavation permit UT10-006E-Gs). For specific locality information, see the “Systematic Paleontology” section below.

After discovery in 2009, the specimen was stabilized with polyvinyl acetate (Vinac™PVA-15, McGean Rohco, Inc., Cleveland, Ohio, USA) dissolved in acetone. Because of weathering, portions of the pedal phalanges and the right half of the skull were collected in 2009, separately from the rest of the skeleton. Surface dry screening uncovered additional bone fragments. During the 2010 field season, the specimen was encased in a plaster and burlap field jacket and airlifted from Grand Staircase-Escalante National Monument by helicopter. Subsequently, the fossil was mechanically prepared using pneumatic engravers of varying sizes (PaleoTools, Brigham City, Utah, USA; Chicago Pneumatic, Independence, Ohio, USA). A minimal amount of matrix was left in place, in order to support and preserve the relative positions of the bones as well as soft tissue impressions. Full field and lab documentation are on file at RAM.

### CT scanning

In order to better visualize internal cranial anatomy, the skull of RAM 14000 was CT scanned on a Toshiba Aquilion 64 scanner at Pomona Valley Hospital Medical Center, Claremont, California, USA. For the large skull blocks, the specimen was initially scanned at 120 kV and 350 mA, slice thickness of 0.5 mm and reconstruction diameter of 300 mm. This resulted in an in-plane resolution of 0.586 mm by 0.586 mm per pixel. After additional preparation, the specimen was rescanned. The left side of the skull was scanned at 120 kV and 400 mA, slice thickness of 0.5 mm, and reconstruction diameter of 229.687 mm, using a standard bone reconstruction algorithm, resulting in an in-plane resolution of 0.45 mm by 0.45 mm per pixel. The isolated portion of the braincase and maxilla were also scanned at identical parameters except for a reconstruction diameter of 140.625 mm, resulting in an in-plane resolution of 0.274 mm by 0.274 mm. The resulting data were then segmented and measured in 3D Slicer 4.2 (available at www.slicer.org; Gering et al., 1999; Pieper, Halle & Kikinis, 2004; Pieper et al., 2006). Because of internal fracturing of the specimen and areas of poor contrast between bone and matrix, a combination of automatic thresholding and manual segmentation were used in order to visualize endocranial features. All CT scan and segmentation data are reposited at Figshare (Table 1, Article S1), and downsampled versions of the mesh are contained in Figs. S1 and S2.

**Table 1:** Summary of digital data available via Figshare. Additional information is contained in Article S1.

Element	Data type	URL
Braincase	Segmentation data	http://dx.doi.org/10.6084/m9.figshare.664171
Braincase	CT scan data	http://dx.doi.org/10.6084/m9.figshare.664167
Braincase	Surface models	http://dx.doi.org/10.6084/m9.figshare.692150
Maxilla	CT scan data	http://dx.doi.org/10.6084/m9.figshare.664168
Skull (left half)	CT scan data	http://dx.doi.org/10.6084/m9.figshare.664169
Skull (left half)	Segmentation data	http://dx.doi.org/10.6084/m9.figshare.691047
Skull (left half with structures)	Surface models	http://dx.doi.org/10.6084/m9.figshare.692151
Skull (left half)	Surface model	http://dx.doi.org/10.6084/m9.figshare.692152
Skull and neck (right half)	CT scan data	http://dx.doi.org/10.6084/m9.figshare.664170
Skull and neck (right half)	Segmentation data	http://dx.doi.org/10.6084/m9.figshare.691053
Skull and neck (right half)	Surface models	http://dx.doi.org/10.6084/m9.figshare.692153
Squamosal (right)	Surface models	http://dx.doi.org/10.6084/m9.figshare.797519
Skeleton (right side)	Surface model	http://dx.doi.org/10.6084/m9.figshare.796442
Humerus (right)	Surface model	http://dx.doi.org/10.6084/m9.figshare.692155
Hind limb (right)	Surface model	http://dx.doi.org/10.6084/m9.figshare.796441
Pedal phalanges (right)	Surface models	http://dx.doi.org/10.6084/m9.figshare.797520
Pedal ungual (right)	Surface models	http://dx.doi.org/10.6084/m9.figshare.797515

### Photogrammetry

Because the humerus was preserved as a natural mold, we produced a digital cast of the element using photogrammetry. 12 color photos at 4000 × 3000 pixel resolution were acquired with a Nikon CoolPix L22 digital camera (Nikon Inc., Melville, New York, USA), and were resized to 2000 × 1500 pixels. Data were processed using BundlerTools (available at server.topoi.hu-berlin.de/groups/bundlertools/), which in turn uses Bundler 0.4, CMVS, and PMVS2. The resulting raw point cloud was processed further in MeshLab 1.3.0 (available at www.meshlab.org), in which a surface mesh was produced using a Poisson surface reconstruction algorithm (Octree Depth = 10, Solver Divide = 9, 1 sample per node, Surface Offsetting = 1). Because the original mesh represented a natural mold, normals were inverted to produce a digital cast. The mesh was scaled by comparison with measurements of the original specimen, and data were exported in STL file format. A downsampled version of the mesh is contained in Fig. S4.

A similar procedure was used with a series of photos of the right side of the skeleton, to produce additional 3D renderings. Photographs at 2848 × 4288 pixel resolution were acquired with a Nikon D90 SLR digital camera (Nikon, Inc., Melville, New York, USA) fitted with a Tamron 179D lens (Tamron Co., Ltd., Saitama, Japan), and were resized to 2000 × 1500 pixels. Ten separate reconstructions were generated, for the ventral, central, and caudal portions of the rib cage (utilizing 15, 24, and 24 photos, respectively), femur (16 photos), tibia and fibula (29 photos), pes (27 photos), pelvic region (21 photos), skull (17 photos), tail (24 photos), and dorsal view of the skeletal block (18 photos). The point clouds were aligned and meshed in MeshLab (Poisson surface reconstruction algorithm, Octree Depth = 12, Solver Divide = 12, 5 samples per node, Surface Offsetting = 1). The original point clouds and surface mesh are reposited at Figshare. A downsampled version of the mesh is contained in Fig. S3. The point clouds for the hind limb were combined and meshed to produce a separate rendering of this part of the body; a downsampled version of this mesh is contained in Fig. S5. All surface meshes are reposited at Figshare (Table 1, Article S1).

### Laser scanning

A disarticulated squamosal and pedal phalanges were laser scanned to produce full-color digital models. The original point clouds were captured using a NextEngine 3D color laser scanner (NextEngine, Inc., Santa Monica, California). For each element, a series of individual scans (varying depending upon the complexity of the element) were acquired at a resolution of 6,200 points/cm^2^. The individual scans were stitched together in ScanStudio HD Pro 1.3.2 (NextEngine, Inc., Santa Monica, California) and fused into a single watertight mesh. All surface meshes, along with full technical details, are reposited at Figshare (Table 1, Article S1).

### Histological sampling

Two samples from the right tibia were extracted for histological analysis. This bone was chosen because of its excellent preservation and easy accessibility on the specimen. Additionally, studies in other ornithischian dinosaurs (the basal iguanodontian *Tenontosaurus tilletti* and the hadrosaurine hadrosaurid *Maiasaura peeblesorum*) suggest that the tibia undergoes less remodeling at midshaft than do other skeletal elements, a characteristic critical for estimating the age of the animal at death using lines of arrested growth (Horner, de Ricqlès & Padian, 2000; Werning, 2012). Thus, the tibia is an ideal element for histological study.

The position of natural cracks in the bone precluded sampling exactly at the tibial mid-diaphysis. However, we were able to sample at two points slightly proximal to this point. The more proximal sample “A” was taken 120 mm from the proximal end of the bone (∼39 percent of the total tibial length, 307 mm), and sample “B” was taken 135 mm (∼44 percent total length) from the proximal end of the bone (Fig. 18D). Prior to sampling, we photographed and molded the surface of this region to document original morphology. Afterward, the sampled region was refilled with plaster to approximate the original anatomy.

We removed both samples using a Dremel Moto-Tool Model 395 rotary tool (Dremel, Inc., Racine, Wisconsin, USA) and small chisel. Because the tibia is partially embedded in matrix, only the caudolateral quadrant of the shaft, rather than a full cross-section, was extracted. We estimate the maximum craniocaudal diameter of the tibia at these points to be 40 mm. Both samples include both compact and cancellous bone; the cortex of sample A is ∼12 mm thick, and sample B is ∼15–16 mm thick. The longitudinal sections made from sample A span 12 mm (proximo-distally) along the diaphysis. Given the maximum diameter relative to the thickness of the sections, and that the medullary cavity is open (i.e., not completely filled by cancellous bone) at both points, we think our samples likely capture most if not all of the preserved histology in this quadrant of the bone.

Histological samples were prepared by S Werning at UCMP. Before embedding, the periosteal surfaces were cleaned with acetone to remove any traces of polyvinyl acetate. Both samples were then embedded in Silmar-41 clear polyester casting resin (Interplastic Corporation, Saint Paul, Minnesota, USA) catalyzed with methyl ethyl ketone peroxide (Norac, Inc., Helena, Arkansas, USA) at 1 percent by mass and allowed them to cure for 48 h at room temperature. Thick transverse (cross-sectional) sections (1–1.5 mm) were cut using a diamond-tipped wafering blade on a low-speed Isomet lapidary saw (Buehler, Inc., Lake Bluff, Illinois), mounted to glass slides, and ground to optical clarity using the materials and methods described in Werning (2012). Two slides in transverse section were made from sample A and three from sample B. Additionally, two slides in longitudinal section were made from some of the remaining embedded portion of sample A. All histological slides are reposited at RAM.

### Histological imaging

All slides were examined under regular transmitted light, elliptically polarized light (i.e., using a full wave retarder or red tint plate, λ = 530 nm) and crossed plane polarizing filters. The filters were used to enhance birefringence. Overlapping digital images photographs (50% overlap by eye in X and Y directions) were taken using a D300 DSLR camera (Nikon Inc., Melville, New York, USA) mounted to an Optiphot2-Pol light transmission microscope (Nikon Inc.). To image the entire slide or radial “transects”, digital images were photomontaged using Autopano Giga 2.0 64Bit (Kolor, Challes-les-Eaux, France), using the program settings described in Werning (2012).

High-resolution histological images are digitally reposited online for scholarly use at MorphoBank (http://morphobank.org/permalink/?P836, project p836; see Table 2 for a list of accession numbers). Digital images larger than 25,000 pixels in either dimension were digitally reduced (preserving original dimension ratios) to allow processing on MorphoBank. These edits were made after scale bars had been added.

**Table 2:** MorphoBank (project 836) accession numbers for high-resolution histological images used in this study.

Section	View	Accession #	Image contents
A	XS	M193513	Entire section A (entire slide), brightfield
		M193511	Radial transect through section A, brightfield
		M283554	Inner cortex, brightfield
		M283547	Inner cortex, brightfield
		M283550	Inner cortex, elliptically polarized light
		M283548	Mid-cortex, brightfield
		M283551	Mid-cortex, elliptically polarized light
		M283553	Outer cortex, brightfield
		M283549	Outer cortex, brightfield
		M283552	Outer cortex, elliptically polarized light
		M193514	Osteocytes
		M193515	Osteocytes
A	LS	M193522	Entire section A (entire slide), brightfield
		M283543	Inner cortex, brightfield
		M283544	Mid-cortex, brightfield
		M283545	Outer cortex, brightfield
		M283546	Osteocytes
B	XS	M151601	Entire section B (entire slide), brightfield
		M193512	Radial transect through section B, brightfield
		M283539	Inner cancellous region, brightfield
		M283540	Outer cancellous region, brightfield
		M283537	Inner cortex, brightfield
		M283541	Inner/mid-cortex, brightfield
		M283538	Outer cortex, brightfield
		M283542	Outer cortex, brightfield

**Notes.**

LS longitudinal section XS cross (transverse) section

These images can be accessed online at: http://www.morphobank.org/permalink/?P836.

### Linear measurements

**Figure 5:**
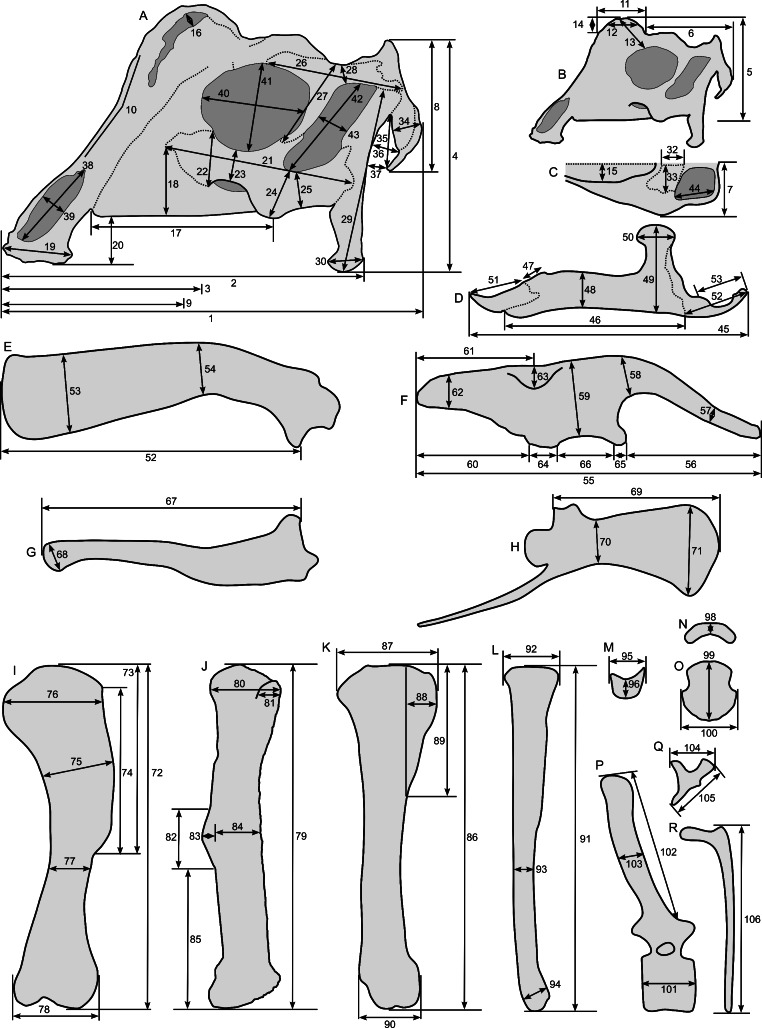
Standards for skeletal measurements. Those for the skull and lower jaw augment standards published elsewhere (Dodson, 1975; Evans, 2010). Numbers associated with each measurement correspond to those in Tables 3–9. (A) and (B) skull in left lateral view; (C) right half of caudal section of skull in dorsal view; (D) mandible in left lateral view; (E) scapula; (F) ilium; (G) ischium; (H) pubis; (I) humerus; (J) femur; (K) tibia; (L) fibula; (M) calcaneum; (N) pedal phalanx; (O) pedal ungual; (P) caudal vertebra (also used for other vertebrae); (Q) cervical rib (also used for sacral rib); (R) dorsal rib. (A–M) and (P–R) are in right lateral view; (N) and (O) are in dorsal view. Drawings are not to scale.

Linear measurements under 300 mm were measured to the nearest 0.l mm with digital calipers, and non-linear measurements as well as those over 300 mm were measured to the nearest mm with a cloth measuring tape. Landmarks for most cranial measurements were patterned after those in Dodson (1975) and Evans (2010), and are diagrammed along with postcranial measurements in Fig. 5. Relevant measurements are contained in Tables 3–10.

**Table 3:** Measurements of the skull of *Parasaurolophus* sp., RAM 14000. The standards for these measurements (modified from those in Dodson, 1975, and Evans, 2010) are diagrammed in Fig. 5.

Element	Measurement and description		Value (mm)	
			Left	Right
Skull	1	Length from tip of rostrum to paroccipital process, parallel to maxillary tooth row	246.0	—
	2	Length from tip of rostrum to quadrate, parallel to maxillary tooth row	211.2	—
	3	Preorbital length, parallel to maxillary tooth row	125.2	—
	4	Height at caudal end, perpendicular to maxillary tooth row	142.0	—
	5	Height from maxillary tooth row to top of crest	120.1	—
	6	Length from caudal end of crest to paroccipital process	112.8	—
	7	Maximum width across postorbitals from midline	46.1	—
	8	Height of caudal plane, perpendicular to tooth row	81.6	—
External naris	38	Maximum length	55.1	—
	39	Maximum width	21.9	—
Orbit	40	Maximum length	62.4	58.9
	41	Maximum height	53.5	48.0
Infratemporal fenestra	42	Maximum length	63.7	—
	43	Maximum width	18.8	20.1
Supratemporal fenestra	44	Maximum length on lateral edge	42.6	—

**Notes.**

Dashes indicate missing measurements.

**Table 4:** Measurements of individual cranial bones of *Parasaurolophus* sp., RAM 14000. The standards for these measurements (modified from those in Dodson, 1975; Evans, 2010) are diagrammed in Fig. 5.

Element	Measurement and description		Value (mm)	
			Left	Right
Crest	9	Length from rostrum to crest midpoint, parallel to tooth row	118.7	—
	10	Angle between crest and snout	120°	—
	11	Length parallel to tooth row, level with skull roof	61.8	—
	12	Length of crest at half-height	46.9	—
	13	Crest height above orbit, from postorbital/prefrontal suture	61.8	—
	14	Height of crest above skull roof	25.1	—
	15	Maximum width of crest from midline	46.1	—
	16	Maximum width of premaxillary-nasal fontanelle	9.4	—
Maxilla	17	Length along tooth row	117.7	110.1^*^
	18	Height from tooth row to jugal-maxilla suture	44.4^*^	31.8
Premaxilla	19	Straight-line length of oral margin, from midline	42.9	—
	20	Depression of oral margin below maxillary tooth row	32.6	—
Jugal	21	Maximum length	113.1^*^	107.7
	22	Maximum width of rostral process	—	35.6
	23	Minimum width below orbit	19.2	19.2
	24	Maximum width of blade	30.9	31.8
	25	Minimum width of quadrate process	22.2	21.7
Postorbital	26	Maximum length	83.7	—
	27	Maximum height	53.4	—
	28	Minimum width of caudal process	13.3	—
Quadrate	29	Maximum length	109.9	112.3
	30	Rostrocaudal length of lateral edge of distal condyle	18.2	17.6
	31	Mediolateral width of distal condyle (not shown)	21.4	—
Frontal	32	Length at midline	34.2	—
	33	Maximum width from midline	31.7	—
Paroccipital process	34	Maximum width	18.3	—
	35	Maximum length	32.4	—
	36	Maximum separation from quadrate	15.9	11.0
	37	Minimum separation from quadrate	11.1	9.9

**Notes.**

Dashes indicate missing measurements.

* indicates approximate measurement.

**Table 5:** Measurements of the lower jaw of *Parasaurolophus* sp., RAM 14000. The standards for these measurements (modified from those in Dodson, 1975) are diagrammed in Fig. 5.

Element	Measurement and description		Value (mm)	
			Left	Right
Mandible	45	Maximum length	223.5	—
Dentary	46	Maximum length along ventral edge	134.2	142.1
	47	Length of edentulous process to caudal edge of predentary	15.3	—
	48	Maximum height from ventral edge to alveoli	30.7	33.1
	49	Maximum height at coronoid process	—	72
	50	Maximum width of coronoid process	—	27.9
Predentary	51	Length parallel to midline	49.8	—
Surangular	52	Maximum length	54.8	46.2
	53	Length of retroarticular process	42.6	38.5

**Notes.**

Dashes indicate missing measurements.

**Table 6:** Measurements of the vertebrae of *Parasaurolophus* sp., RAM 14000. The standards for these measurements are diagrammed in Fig. 5.

Element	Measurement and description		Value (mm)
Cervical vertebra ?5	101	Maximum length of centrum	20.0
Cervical vertebra ?6	101	Maximum length of centrum	20.0
Dorsal vertebra ?14	101	Maximum length of centrum	28.8
Dorsal vertebra ?15	101	Maximum length of centrum	27.3
Dorsal vertebra ?16	101	Maximum length of centrum	26.7
Dorsal vertebra ?17	101	Maximum length of centrum	30.8
Dorsal vertebra ?18	101	Maximum length of centrum	35.8
Caudal vertebra ?2	103	Maximum craniocaudal length of neural spine	16.5
Caudal vertebra ?3	101	Maximum length of centrum	21.0
	103	Maximum craniocaudal length of neural spine	16.1
Caudal vertebra ?4	101	Maximum length of centrum	20.3
	103	Maximum craniocaudal length of neural spine	15.9
Caudal vertebra ?5	101	Maximum length of centrum	18.5
	103	Maximum craniocaudal length of neural spine	14.9
Caudal vertebra ?6	101	Maximum length of centrum	16.7
	102	Maximum proximodistal length of neural spine	107.9^*^
	103	Maximum craniocaudal length of neural spine	13.6
Caudal vertebra ?7	101	Maximum length of centrum	19.4
	103	Maximum craniocaudal length of neural spine	15.0
Caudal vertebra ?8	101	Maximum length of centrum	16.5
	103	Maximum craniocaudal length of neural spine	11.5
Caudal vertebra ?9	101	Maximum length of centrum	67.3
	103	Maximum craniocaudal length of neural spine	13.6
Caudal vertebra ?10	103	Maximum craniocaudal length of neural spine	10.3
Caudal vertebra ?12	101	Maximum length of centrum	19.3
	103	Maximum craniocaudal length of neural spine	8.6
Caudal vertebra ?13	101	Maximum length of centrum	19.5
	102	Maximum proximodistal length of neural spine	30.9
Caudal vertebra ?14	101	Maximum length of centrum	18.3
	102	Maximum proximodistal length of neural spine	29.2
Caudal vertebra ?15	101	Maximum length of centrum	19.1
	102	Maximum proximodistal length of neural spine	29.2
Caudal vertebra ?16	101	Maximum length of centrum	20.5
	103	Maximum craniocaudal length of neural spine	9.8
Caudal vertebra ?17	101	Maximum length of centrum	20.3
	103	Maximum craniocaudal length of neural spine	9.4
Caudal vertebra ?18	101	Maximum length of centrum	21.3
Caudal vertebra ?19	102	Maximum proximodistal length of neural spine	49.1

**Notes.**

* indicates approximate measurement.

**Table 7:** Measurements of the ribs of *Parasaurolophus* sp., RAM 14000. The standards for these measurements are diagrammed in Fig. 5.

Element	Measurement and description		Value (mm)
Cervical rib ?4	104	Maximum width between capitulum and tuberculum	19.6
	105	Maximum length from capitulum to distal end of shaft	27.1
Cervical rib ?5	104	Maximum width between capitulum and tuberculum	19.7
	105	Maximum length from capitulum to distal end of shaft	29.3
Cervical rib ?6	104	Maximum width between capitulum and tuberculum	17.9
	105	Maximum length from capitulum to distal end of shaft	30.8
Dorsal rib 1 (left)	106	Maximum length from capitulum to distal end of shaft	235.0
Dorsal rib 2 (left)	106	Maximum length from capitulum to distal end of shaft	285.0
Dorsal rib 3 (left)	106	Maximum length from capitulum to distal end of shaft	325.0
Sacral rib 1	104	Maximum width between capitulum and tuberculum	39.4
Sacral rib 1	105	Maximum length from capitulum to distal end of shaft	54.3
Torso length (left)		Distance between scapular glenoid and pelvic acetabulum	620.0
Rib cage		Maximum depth	339.0

**Table 8:** Measurements of the pectoral and pelvic elements of *Parasaurolophus* sp., RAM 14000. The standards for these measurements are diagrammed in Fig. 5.

Element	Measurement and description		Value (mm)
Scapula (left)	52	Maximum length	267.6^*^
	53	Maximum width of blade	55.0
	54	Minimum width of blade	36.3
Ilium	55	Greatest length	300.8
	56	Length of preacetabular process	120.7
	57	Minimum height of preacetabular process	21.5
	58	Maximum height of preacetabular process	36.8
	59	Maximum height of ilium	60.9
	60	Length of postacetabular process, ventral	89.9
	61	Length of postacetabular process, dorsal	104.7
	62	Minimum height of postacetabular process	30.8
	63	Mediolateral width of supraacetabular process	28.4
	64	Length of ischiadic peduncle	28.7
	65	Length of pubic peduncle	31.6
	66	Width of acetabulum	52.1
Ischium (left)	67	Maximum length	243.1
	68	Maximum width of distal end	30.0
Pubis	69	Length of prepubic blade	147.3
	70	Maximum depth of prepubic blade	83.0
	71	Minimum depth of prepubic blade	46.2

**Notes.**

* indicates approximate measurement.

**Table 9:** Measurements of the limb bones of *Parasaurolophus* sp., RAM 14000. The standards for these measurements are diagrammed in Fig. 5.

Element	Measurement and description		Value (mm)
Humerus	72	Maximum length	174.6
	73	Length of deltopectoral crest (1)	101.1
	74	Length of deltopectoral crest (2)	96.6
	75	Maximum width at deltopectoral crest	38.1
	76	Maximum width at proximal end	50.5
	77	Minimum diameter of diaphysis	24.5
	78	Maximum width at distal end	35.2
Femur	79	Maximum length	328.9
	80	Craniocaudal width of proximal end on lateral surface	66.8
	81	Craniocaudal length of cranial trochanter	25.8
	82	Proximodistal length of 4th trochanter	73.3
	83	Craniocaudal height of 4th trochanter	15.6
	84	Craniocaudal length at midshaft excluding 4th trochanter	40.9
	85	Distance between distal ends of 4th trochanter and femur	127.2
Tibia	86	Maximum length	306.9
	87	Maximum craniocaudal width at proximal end	80.6
	88	Maximum projection of cnemial crest	22.1
	89	Maximum proximodistal length of cnemial crest	113.0
	90	Maximum craniocaudal width at distal end	47.5
Fibula	91	Maximum length	288.3
	92	Maximum craniocaudal diameter at proximal end	43.2
	93	Minimum craniocaudal diameter of diaphysis	15.7
	94	Maximum craniocaudal diameter at distal end	25.2
Calcaneum	95	Maximum craniocaudal length	25.4
	96	Minimum proximodistal length	14.6
Metatarsal IV	97	Maximum length on dorsal midline (not shown)	100.1
Phalanx IV-1	98	Maximum length on dorsal midline	25.9
Phalanx IV-2	98	Maximum length on dorsal midline	8.8
Phalanx IV-3	98	Maximum length on dorsal midline	7.3
Phalanx IV-4	98	Maximum length on dorsal midline	2.3
Phalanx IV-5	99	Maximum length on dorsal midline	31.3
Phalanx IV-5	100	Maximum mediolateral width (estimated)	25.6
Phalanx III-2	98	Maximum length on dorsal midline	7.3
Phalanx III-3	98	Maximum length on dorsal midline	5.1
Phalanx III-4	99	Maximum length on dorsal midline	29.0

**Table 10:** Measurements of *Parasaurolophus* sp., RAM 14000, compared with those for selected other lambeosaurines. Measurements of *Parasaurolophus* sp., RAM 14000, compared with those for selected other lambeosaurines. Measurements for FMNH P 27393 are from Ostrom (1963), and measurements for other lambeosaurines (excepting RAM 14000) are from Lull & Wright (1942), Evans (2010), and Sullivan & Williamson (1999). The crest length for AMNH 5340 is estimated from photographs; the crest length for FMNH P 27393 is approximate. All cranial measurements follow those of Dodson (1975) and Evans (2010); crest length is from the top of the orbit to the maximum extent of the crest. AMNH 5340 is included as the most complete and best-known associated skeleton of a juvenile lambeosaurine. Complete measurements, as well as a description of the landmarks used for each measurement, are contained in Fig. 5 and Tables 2–8.

Taxon	*Parasaurolophus* sp.	*P. cyrtocristatus*	*P. walkeri*	*Lambeosaurus* sp.
**Specimen**	RAM 14000	FMNH P27393	ROM 768	AMNH 5340
Humerus length (mm)	175	565 (0.31)	520 (0.34)	305 (0.57)
Ilium length (mm)	301	975 (0.31)	1015 (0.30)	570 (0.53)
Prepubis length (mm)	147	430 (0.34)	516 (0.28)	260^*^ (0.57)
Ischium length (mm)	243^*^	1040 (0.23)	–	630^*^ (0.39)
Femur length (mm)	329	1105 (0.30)	1032 (0.32)	590 (0.56)
Tibia length (mm)	307	–	–	550 (0.56)
Fibula length (mm)	288	890 (0.32)	–	530^*^ (0.54)
MT IV length (mm)	100	335 (0.30)	–	–
Fibula/femur	0.88	0.80	–	0.90
Skull length (mm)	246	–	745 (0.33)	380 (0.65)
Quadrate length (mm)	111	–	272 (0.41)	165 (0.67)
Orbit length (mm)	60	–	105 (0.57)	77 (0.78)
Orbit height (mm)	50	–	170 (0.29)	82 (0.61)
Dentary length (mm)	138	–	455 (0.30)	–
Crest length (mm)	62	404^*^ (0.15)	970 (0.06)	90^*^ (0.69)

**Notes.**

The number in parentheses in each entry indicates the size relative to RAM 14000.

* indicates an incomplete or estimated element length.

### Skeletal completeness

In order to assess relative skeletal representation for the three most complete specimens of *Parasaurolophus* (FMNH P 27393, RAM 14000, and ROM 768), we tallied the preserved elements for each. The skull and mandible were considered a single unit, as were the sacrum and sacral ribs. Partial elements were counted as present in the specimen, and we only counted bilateral elements once (e.g., even if both humeri were present, this element was counted only once). Tallies are contained in Table S1.

### Nomenclatural conventions

In this paper, the following conventions are utilized. These are defined here so as to avoid confusion in the event of future systematic or phylogenetic revisions. Following the recent formal definition by Prieto-Márquez and colleagues (2013), the clade Lambeosaurini (lambeosaurins) includes all taxa closer to *Lambeosaurus lambei* than to *Parasaurolophus walkeri*, *Tsintaosaurus spinorhinus*, or *Aralosaurus tuberiferus*. This clade is approximately equivalent to the informally used but never formally defined “Corythosaurini” (Godefroit, Alifanov & Bolotsky, 2004; Evans & Reisz, 2007). Unless otherwise specified, comparisons here involve the North American genera *Corythosaurus*, *Lambeosaurus*, *Hypacrosaurus*, and *Velafrons*, as well as the Asian taxon *Nipponosaurus*. The clade Parasaurolophini (parasaurolophins) includes all taxa closer to *Parasaurolophus walkeri* than to *Lambeosaurus lambei*, *Tsintaosaurus spinorhinus*, or *Aralosaurus tuberiferus* (Godefroit, Alifanov & Bolotsky, 2004; Evans & Reisz, 2007; Prieto-Márquez et al., 2013). This includes two genera, *Parasaurolophus* and *Charonosaurus*. Unless otherwise specified, usage of the name *Parasaurolophus* alone refers to all three named species, *P. walkeri*, *P. cyrtocristatus*, and *P. tubicen*.

## Results

### Systematic paleontology

Dinosauria Owen, 1842
Ornithischia Seeley, 1888
Hadrosauridae Cope, 1869
Lambeosaurinae Parks, 1923
*Parasaurolophus* Parks, 1922
*Parasaurolophus* sp.

### Referred material

RAM 14000, a partial skull and articulated skeleton (Figs. 2 and 3).

### Locality and horizon

RAM V200921, Grand Staircase-Escalante National Monument, Garfield County, Utah, USA (Fig. 1); upper part of middle unit (sensu Roberts, 2007) of the Kaiparowits Formation; Late Cretaceous (late Campanian; Roberts, Deino & Chan, 2005). The site is stratigraphically between two locally prominent bentonites, tentatively correlated with bentonites KBC-109 and KBC-144 of Roberts, Deino & Chan (2005), both exposed less than 10 km away from RAM V200921 and dated to 75.51 + −0.15 Ma (Roberts et al., 2013). The specimen was preserved within a cross-bedded tabular sandstone, tentatively interpreted as a channel deposit following previous literature (Roberts, 2007). Detailed locality data are on file at the RAM and are available to qualified investigators upon request.

## Description

RAM 14000 is preserved in nearly perfect articulation, with the neck, hip, lower leg and metatarsals strongly flexed (opisthotonic posture, probably resulting from the fresh carcass’s immersion in water; Reisdorf & Wuttke, 2012; Figs. 2 and 3, Fig. S3). The right humerus and pedal digits are gently extended. The specimen was lying on its left side; although more bones are represented on this side, they are much more badly weathered than on the right. Tree roots, freeze-thaw cycles, and recent rodent activity fragmented and displaced many of the elements on the left side. In contrast, the right side is less complete in terms of element representation, but the quality of bone preservation is generally better than on the left side.

### Skull and mandible

**Figure 6:**
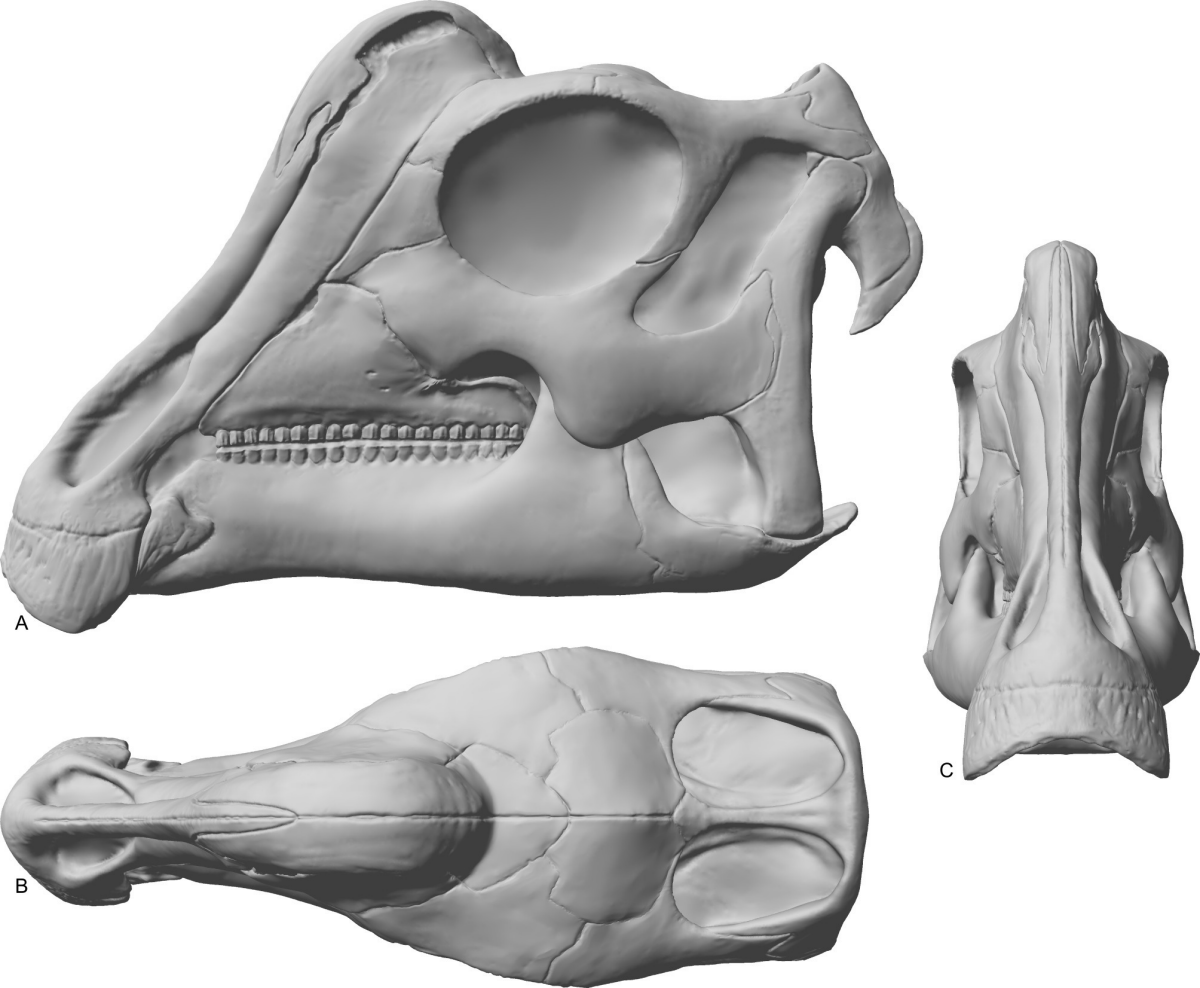
Reconstruction of the skull of *Parasaurolophus* sp., RAM 14000. (A) lateral view; (B) dorsal view; (C) rostral view. Missing elements (including sutural relationships that are not visible in RAM 14000) are patterned after other lambeosaurines, and the rhamphotheca is shown in place. Reconstruction copyright Ville Sinkkonen.

The skull of RAM 14000 was split in two (parasagittally) by erosion; in order to preserve visibility of internal structures, the two halves have not been reassembled. The skull and mandible are in articulation, with only slight displacement of the quadrate and mandible relative to each other. The left side is more complete, preserving nearly all elements (with the exception of a portion of the premaxilla). The dorsal and rostral portions of the right side are missing, with the exception of some elements (such as the maxilla, parts of the dentary, and braincase) that were separated from the main block by erosion. A digital reconstruction, based on RAM 14000 with missing sections modeled after juvenile lambeosaurins, is presented in Fig. 6. Measurements are included in Tables 3–5.

**Figure 7:**
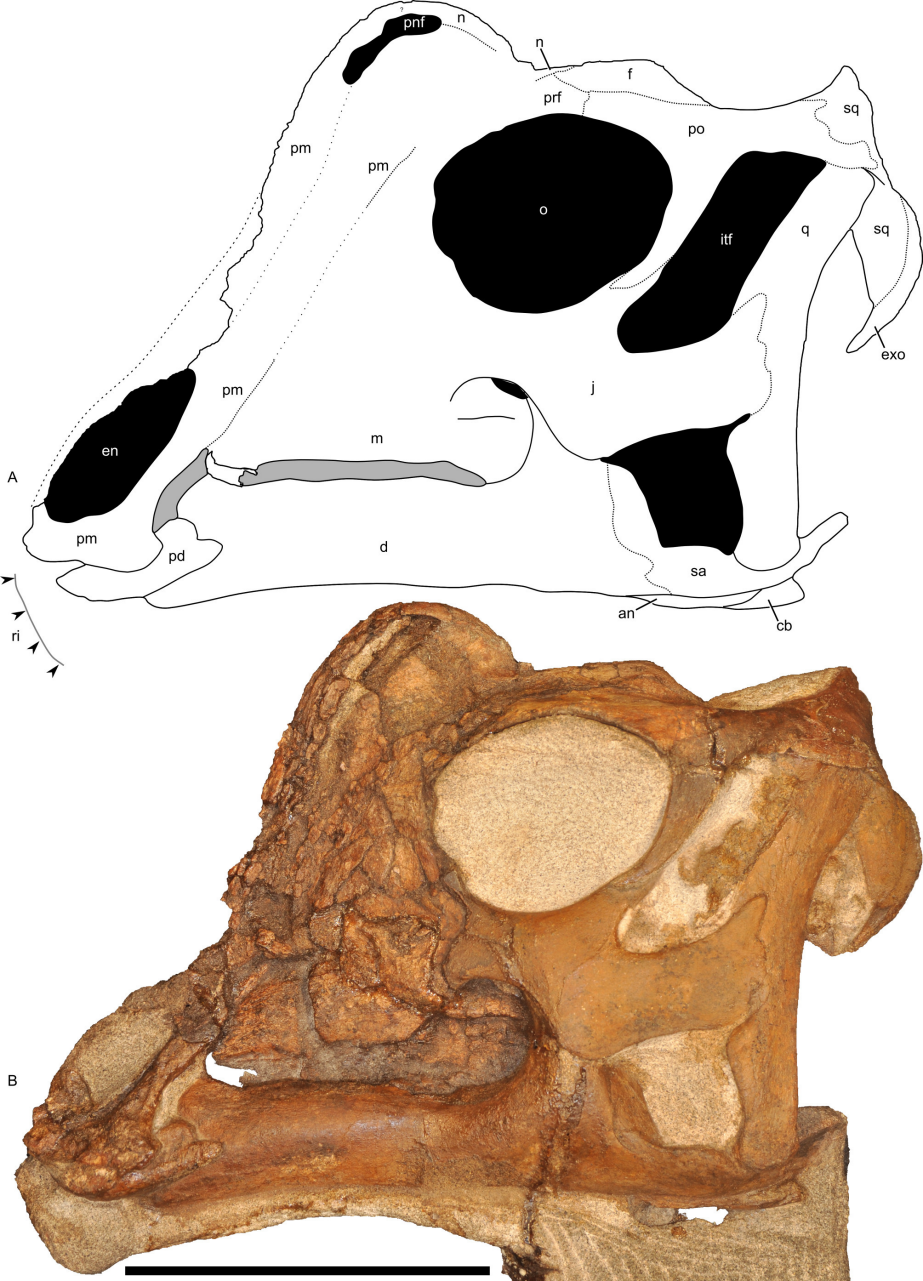
Left half of the skull of *Parasaurolophus* sp., RAM 14000, in lateral view. (A) interpretive drawing; (B) photograph. Abbreviations: an, angular; cb, first ceratobranchial; d, dentary; en, external naris; exo, exoccipital-opisthotic; f, frontal; itf, infratemporal fenestra; j, jugal; m, maxilla; n, nasal; o, orbit; pd, predentary; pm, premaxilla; pnf, premaxilla-nasal fontanelle; po, postorbital; prf, prefrontal; q, quadrate; ri, extent of impressions of upper rhamphotheca; sa, surangular; sq, squamosal. Scale bar equals 10 cm.

In lateral view (Fig. 7), the skull has a profile typical of a juvenile hadrosaur–squared caudally and triangular rostrally. The orbit is proportionately large and slightly longer than tall. The infratemporal fenestra is inclined caudally and quite narrow, with a slight constriction at its midpoint. Because the midline of the skull is missing, the exact shape of the supratemporal fenestra is unknown. However, the preserved portion is roughly trapezoidal. Individual bones and skull regions are described below.

#### Premaxilla

The premaxilla is the most prominent cranial bone in lateral view, extending from the upper “beak” to the dorsum of the skull. The bone is roughly divisible into three portions: a lower portion including the oral margin and external (bony) naris as well as caudodorsal and caudolateral processes that form the remainder of the premaxilla and much of the crest.

The rostroventral-most segment of the premaxilla forms the dorsal oral margin. In lateral view (Fig. 7), most of the edge of the beak is straight and only slightly inclined (relative to the maxillary tooth row), contrasting with the more inclined surface seen in most other lambeosaurine specimens (Evans, 2010), including *Parasaurolophus walkeri* (ROM 768). Furthermore, the caudal corner of the beak is sharply hooked to form a tab-like process below a broadly concave postoral margin. Although this process occurs to varying degrees in many lambeosaurines of all ontogenetic stages (Evans, 2010), the condition in RAM 14000 is unusually prominent and most similar to that in *Parasaurolophus walkeri* (Parks, 1922; Sullivan & Williamson, 1999), particularly in the combination of the tab-like process and rounded postoral margin. The only major difference is that the concavity in the postoral margin is sharper in ROM 768 (*Parasaurolophus walkeri*) than in RAM 14000. Measuring from the midline, the mediolateral width of the oral margin is estimated at 26 mm, and the estimated entire width of the free oral margin (perpendicular to the midline) is thus 52 mm. The oral margin is fairly uniform in outline, with no major denticulations.

The lower portion of the premaxilla encloses the external (bony) naris. The dorsal margin of the bone is eroded away, but its impression is preserved along the narial margin. The bony naris is roughly lenticular, rounded at its distal (rostroventral) end and pointed at its proximal (caudodorsal) end. The depression in the lateral surface of the premaxilla that houses the naris is delimited from the rest of the skull by a gentle ridge that is most prominent caudodorsally.

The caudolateral process of the premaxilla forms the ventral margin of the external (bony) naris and extends caudolaterally. Dorsally, the process contacts the caudodorsal process of the premaxilla. Although much of this suture is extremely fragmented, it appears quite straight along its preserved portions (Fig. 7A). This contrasts with the more sinuous suture seen in juvenile and adult *Hypacrosaurus*, *Corythosaurus*, and *Lambeosaurus* (Evans, 2010; Brink et al., 2011), but more closely matches the fairly straight suture (where it can be discerned) in specimens of *Parasaurolophus* (Sullivan & Williamson, 1999). Similarly, the sutures with the maxilla, lacrimal, and prefrontal, where they can be discerned, are straight, much closer to the condition in *Parasaurolophus* than in lambeosaurins. This may reflect the internal absence of an “S-loop” in the narial passages, a feature that occurs in lambeosaurins (e.g., Weishampel, 1981b; Evans, Ridgely & Witmer, 2009). The ventral portions of the process are comparatively narrow, but the process expands dorsally, where it forms part of the crest. The caudolateral process forms the ventral border of the premaxilla-nasal fontanelle and presumably contacts the nasal at the caudal extent of the fontanelle.

The caudodorsal process of the premaxilla, which forms much of the rostral profile of the skull, is poorly preserved. Its contact with the nasal cannot be interpreted with confidence due to extensive cracking, so no further comment will be offered here.

#### Nasal

Much of the nasal is poorly preserved in gross external view, with the exception of its suture with the frontal and a portion along the caudal margin of the crest (Fig. 7A). The nasal forms the rostrodorsal margin of the premaxilla-nasal fontanelle, as well as the caudal edge of the crest. The dorsal margin of the nasal is strongly rounded and almost horizontal, unlike the peaked margin seen in juvenile lambeosaurins ROM 758 and 759 (*Lambeosaurus* sp. and *Corythosaurus* sp., respectively). The nasal’s suture with the prefrontal is not readily visible, and the contact with the frontal is described with that element. The internasal suture in the crest is flat along the suture’s medial surface.

#### Crest (premaxilla and nasal)

The crest is roughly dome-shaped, with a broad and rounded profile. It is semi-circular in lateral view, with its midpoint rostral to the orbit (Fig. 7). Unlike adult lambeosaurines, including *Parasaurolophus*, the crest does not overhang the frontal. Based on the position of the premaxilla-nasal fontanelle, and its relationships in lambeosaurins, the nasal is inferred to be the bone that bounds the dorsal and caudal margins of the crest (Fig. 7A). The presence of a premaxilla-nasal fontanelle contrasts with its absence in adult *Parasaurolophus* and *Hypacrosaurus altispinus* of all ontogenetic stages, but is similar to juvenile *Corythosaurus*, *Lambeosaurus*, and *Hypacrosaurus stebingeri*, and probably also *Kazaklambia convincens* (Bell & Brink, in press; Horner & Currie, 1994; Evans, Forster & Reisz, 2005; Brink et al., 2011). Unlike juvenile lambeosaurins or *Kazaklambia convincens*, the fontanelle is exceptionally dorsally placed relative to the rest of the crest in RAM 14000.

**Figure 8:**
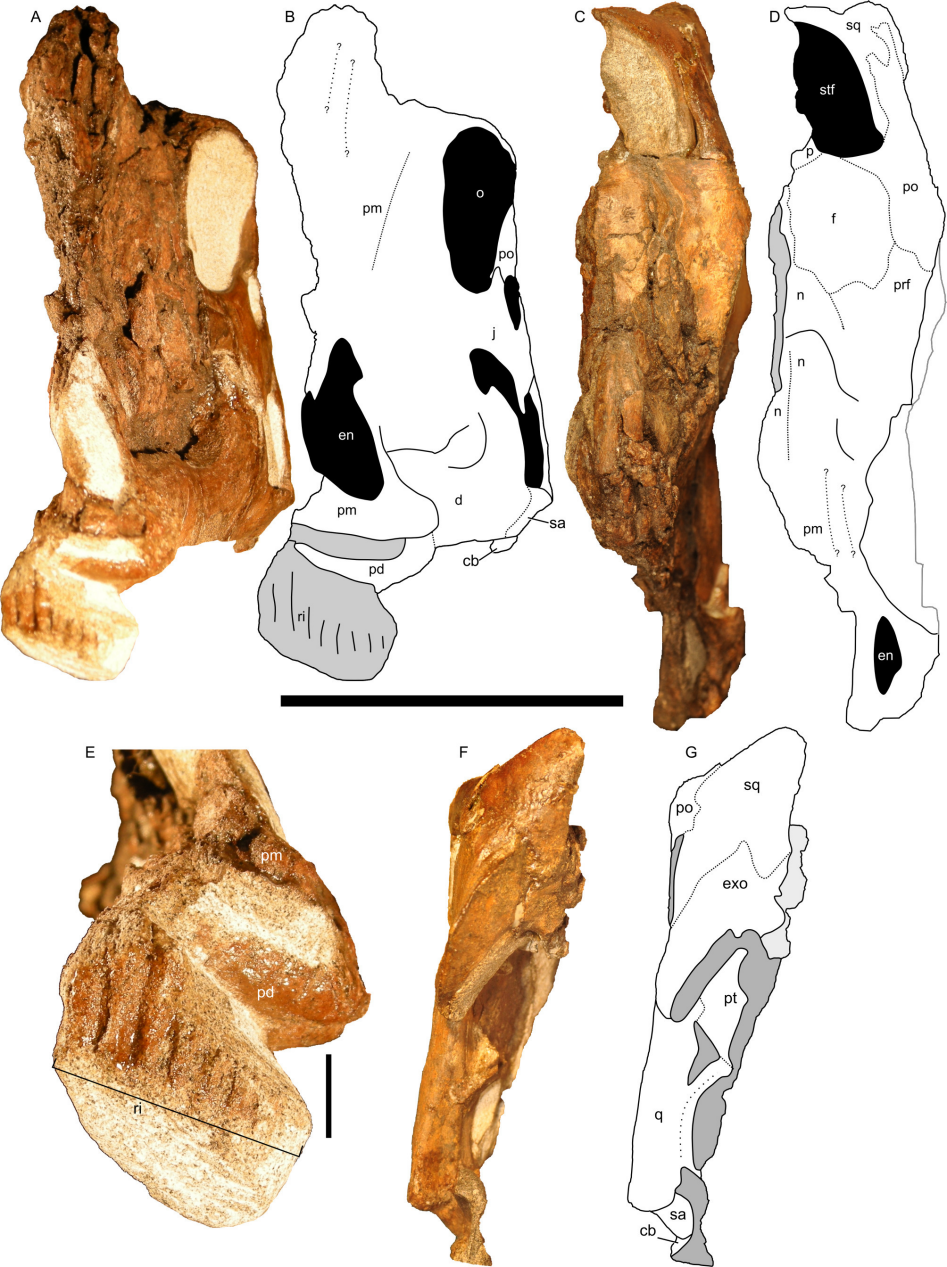
Left half of the skull of *Parasaurolophus* sp., RAM 14000. (A) and (B), rostral view; (C) and (D), dorsal view; (F) and (G) caudal view. (E) detail of the impression of the upper rhamphotheca in rostral view. (A), (C), (E), and (F) are photographs, and (B), (D), and (G) are interpretive line drawings. Abbreviations: cb, first ceratobranchial; d, dentary; en, external naris; exo, exoccipital-opisthotic; f, frontal; j, jugal; n, nasal; o, orbit; p, parietal; pd, predentary; pm, premaxilla; po, postorbital; prf, prefrontal; pt, pterygoid; q, quadrate; ri, impression of rhamphotheca; sa, surangular; sq, squamosal; stf, supratemporal fenestra. Scale bar equals 10 cm for (A–D) and (F–G), and 1 cm for (E). In (A) and (B), scale bar is approximately in the plane of the crest; in (C) and (D), the scale bar is approximately in the plane of the frontal bone; in (F) and (G), the scale bar is in the plane of the quadrate.

In dorsal and rostral view (Figs. 8A–8D), the margins of the crest are strongly rounded. The caudal margin is only gently tapered. This contrasts with the condition in both juvenile and adult lambeosaurins (*Corythosaurus*, *Lambeosaurus*, and *Hypacrosaurus*), in which a thin flange of bone projects from the caudal edge of the crest (Weishampel, 1981b; Evans, Ridgely & Witmer, 2009). In these animals, the flange of bone is not occupied by the nasal passages. In RAM 14000, the nasal passages fill nearly the entirety of the crest, similar to the condition in adult *Parasaurolophus* (Weishampel, 1981b; Sullivan & Williamson, 1999). Furthermore, the crest in RAM 14000 is quite broad, whereas the crest is also fairly narrow along its length in juvenile and adult lambeosaurins.

#### Nasal cavity

**Figure 9:**
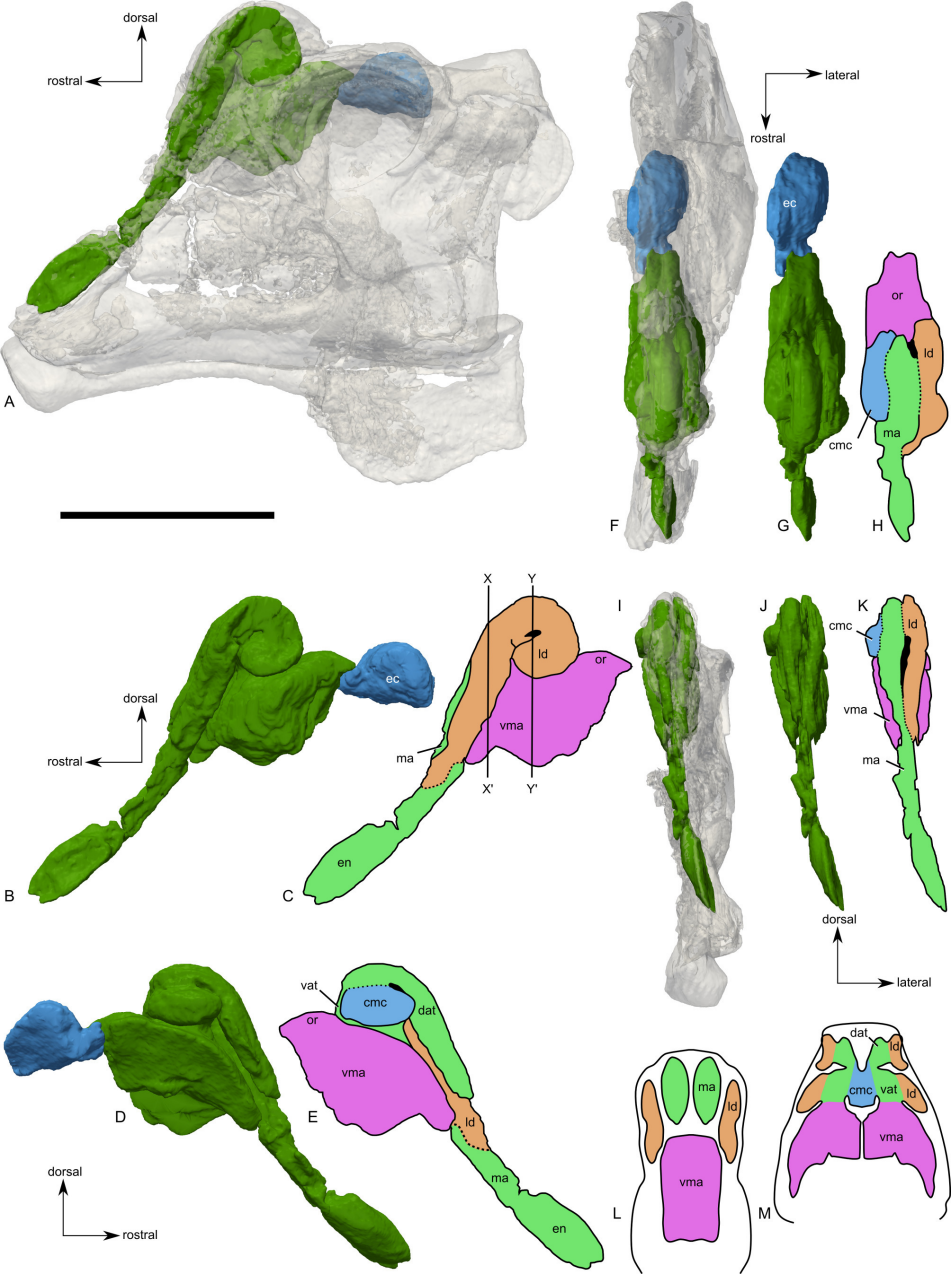
Skull of *Parasaurolophus* sp., RAM 14000, with digital reconstruction showing endocranial features. (A)–(C), left lateral view; (D)–(E), medial view; (F)–(H), dorsal view; (I)–(K), rostral view; (L)–(M), coronal section schematics. (A), (F), and (I) show the endocranial cavity (blue) and nasal passages (green) relative to the cranium, and (B), (D), (G), and (J) show the features without the skull bones. (C), (E), (H), and (K) show a schematic of the various parts of the nasal passages. The positions of the planes of section for (L) and (M) are indicated on (C) as X–X′ and Y–Y′, respectively. Dashed lines in (C), (E), (H), and (K) indicate areas of communication between different parts of the nasal passages. In (E), note that the dorsal ascending tract (dat) is not continuous with the naris; this is due to a missing section of the airway. Abbreviations: cmc, common median chamber (homologous to nasal cavity proper); dat, dorsal ascending tract (homologous to nasal vestibule); ec, endocranial cavity; en, external naris; ld, lateral diverticulum (homologous to nasal cavity proper); ma, main airway (homologous to nasal vestibule); or, olfactory region; vat, ventral ascending tract (homologous to nasopharyngeal duct); vma, ventral portion of main airway. Scale bar equals 10 cm.

The nasal passages are preserved only on the left side and were studied by gross examination of broken surfaces as well as using CT scans (Fig. 9, Fig. S1). Terminology for anatomical structures follows that of Evans, Ridgely & Witmer (2009) and Weishampel (1981b). The airway closest to the external naris is termed “proximal,” and the airway furthest from the naris and closest to the internal choanae is termed “distal.” Portions of the nasal passages and their surrounding bones, particularly the interval immediately caudal to the external naris, are heavily fractured. Furthermore, it appears that some areas were not completely ossified at the time of death, and we hypothesize that some aspects of the chambers may have become more prominently separated later in ontogeny. Thus, we must emphasize that aspects of our digital reconstructions may be subject to alternative interpretation. Points of particular concern are noted as such at the appropriate points in the description.

The external naris is ovoid and strongly elongated (Fig. 7). Part of the main airway distal to this point is fragmented and poorly preserved, but has been reconstructed based on the CT scan data as well as physical examination of the specimen itself. The reconstruction shows the airway to be straight in lateral view (Figs. 9A–9C), with no evidence for an S-loop as seen in lambeosaurins of all known post-embryonic stages (Horner & Currie, 1994; Evans, Ridgely & Witmer, 2009). It is possible that the S-loop simply wasn’t preserved, but based on the contours of the better-preserved distal airway, we do not consider this particularly likely.

**Figure 10:**
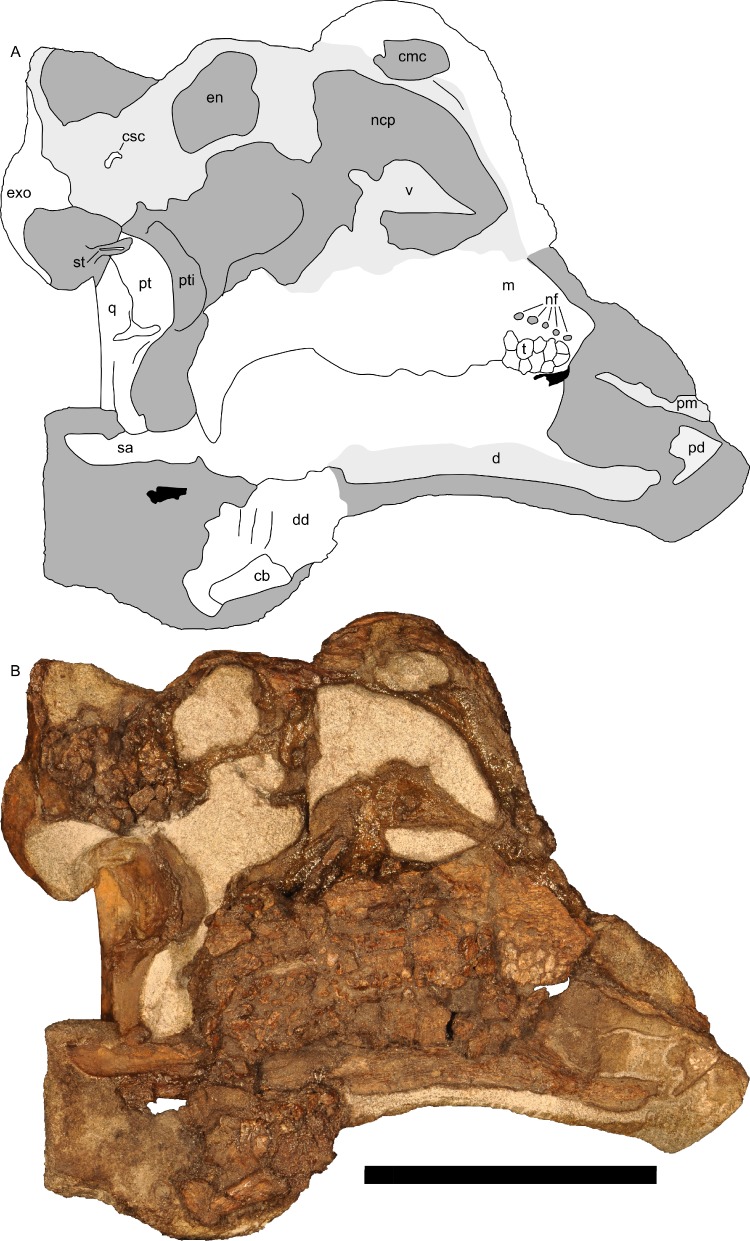
Left half of skull of *Parasaurolophus* sp., RAM 14000, in medial view. (A) interpretive drawing; (B) photograph. Abbreviations: cb, first ceratobranchial; cmc, common medial chamber; csc, caudal semicircular canal; d, dentary; dd, dentition from dentary (displaced); en, endocranial cavity; exo, exoccipital-opisthotic; m, maxilla; ncp, nasal cavity proper; nf, nutrient foramina; pd, predentary; pm, premaxilla; pt, pterygoid; pti, pterygoid impression; q, quadrate; sa, surangular; st, stapes; t, tooth; v, vomer. Scale bar equals 10 cm.

The main airway progresses in the segment known as the dorsal ascending tract, homologous to the nasal vestibule of other sauropsids (Weishampel, 1981b), and continues to the apex of the crest (Figs. 9D and 9E), measuring 170 mm from the proximal end of the airway to the summit of the dorsal ascending tract. At a sharp U-bend, the airway enters the section known as the ventral ascending tract (Figs. 9D and 9E), which drops ventrally to enter the main body of the skull. The ventral ascending tract (homologous to the nasopharyngeal duct of other sauropsids; Weishampel, 1981b) is only 33 mm long and much shorter than the dorsal equivalent. In lambeosaurins, this communication between the main airway and the rest of the skull is reconstructed to occur at the midline via a common median chamber (Evans, Ridgely & Witmer, 2009). By contrast, the airway of RAM 14000 enters the skull separately on both right and left sides, as is more usual for tetrapods. The common median chamber is clearly separated from the ventral aspects of the skull by a thin lamina of bone (preserved as an impression visible in medial view as well as a small piece of bone visible in CT scan; Figs. 9D, 9E, 9M and 10).

The common median chamber of the nasal airway is directly visible on the broken medial surface of the left half of the skull (Figs. 9D, 9E and 10). In profile, this chamber is oval and rostrocaudally elongated (25.5 mm long by 15 mm tall). It is positioned just above the level of the dorsal margin of the skull roof, at the very lower edge of the crest. Relative to the orbit, the common median chamber is dorsal and slightly rostral. This chamber, along with the lateral diverticulum, is probably homologous to the nasal cavity proper of other sauropsids (Weishampel, 1981b).

The lateral diverticulum is prominent, shaped approximately like a shepherd’s crook and coiled clockwise in left lateral view (Figs. 9A–9C). An incompletely ossified lamina separates the diverticulum from the main airway (Figs. 9I–9K); proximally, this coincides with a lamina of bone that may represent the premaxilla-nasal suture. Ventrally, the lateral diverticulum appears to communicate with the main nasal airway within the skull (Fig. 9M). In lambeosaurins, the lateral diverticulum does not communicate directly with the main airway in the skull, but is separated by a bony lamina. We hypothesize that a similar condition occurred in RAM 14000, but that the lamina was not completely ossified at the ontogenetic stage represented here. Density differences in the sediment are faintly visible in CT scan along this line. These are not definitively bone, and the morphology is suggestive of a soft tissue pattern that may have been preserved through early infilling of the skull by sediment (Daniel, 2012). As interpreted here, the lateral diverticulum diverges from the main airway approximately halfway between the external naris and the common median chamber (Fig. 9C). This is a much more proximal origination than in *Corythosaurus* (subadult CMN 34825 and juvenile ROM 759) and *Lambeosaurus* (juvenile ROM 758), but matches the condition seen in *Hypacrosaurus* (adult ROM 702). Thus, the lateral diverticulum is quite extensive in RAM 14000. Unlike *Hypacrosaurus*, however, the lateral diverticulum is not positioned ventrally to the main airway at any point; the two passages are genuinely parallel (as reconstructed for adult *Parasaurolophus*; Weishampel, 1981b). The apex of the lateral diverticulum opens to the premaxilla-nasal fontanelle. Thus, the lateral diverticulum is bordered primarily by the premaxillae, with a small contribution from the nasals.

**Figure 11:**
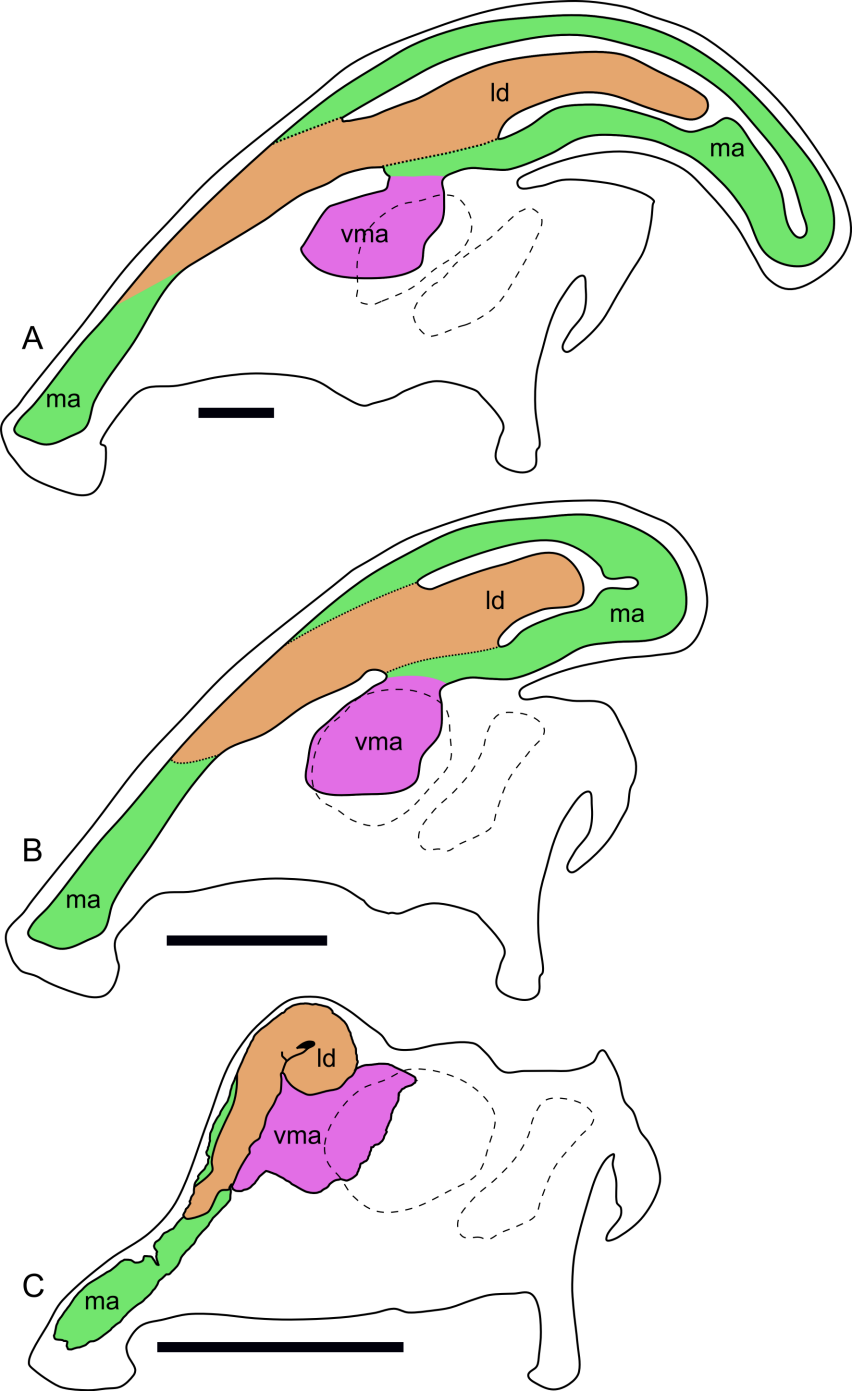
Ontogenetic changes in the nasal passages and crest of *Parasaurolophus*. All images illustrate the condition immediately lateral to the sagittal plane, and rostral is to the left in all images. (A), adult individual, modified after Ostrom (1963). The lateral diverticulum has been altered based on data from RAM 14000, indicating a more proximal origin for the chamber. (B), hypothetical subadult *Parasaurolophus*. (C), juvenile, based on RAM 14000. Note that the intermediate-sized individual is largely speculative, although the enlarged size of the crest is consistent with a referred braincase, CMN 8502 (Evans, Reisz & Dupuis, 2007). In (B) and (C), the dotted lines separating the lateral diverticulum and the main airway indicate that the diverticulum is obscuring the view of the main airway, and the two chambers run parallel to each other. Dashed lines indicate the positions of the left orbit and infratemporal fenestra. Abbreviations: ld, lateral diverticulum; ma, main airway; vma, ventral portion of main airway. Scale bars equal 10 cm.

Compared to reconstructions for *Parasaurolophus cyrtocristatus* and *P. walkeri* (Weishampel, 1981b), RAM 14000 displays several important departures from the adult condition (Fig. 11). Corresponding to the small crest, the nasal passages are much shorter in overall length. Unlike adult specimens, the ventral ascending tract of the nasal passages of RAM 14000 is quite short relative to the dorsal ascending tract. Furthermore, the lateral diverticulum of RAM 14000 is virtually the same length as the dorsal ascending tract. In adult *P. cyrtocristatus* and *P. walkeri*, the lateral diverticulum only extends slightly past the midpoint of the crest (Fig. 11A), and is reconstructed as a blind-ended chamber (Ostrom, 1963; Weishampel, 1981b). This reconstruction should be tested against CT scan data. The hooked morphology of the lateral diverticulum in RAM 14000 is reminiscent of the condition reconstructed for *P. tubicen* (Sullivan & Williamson, 1999).

The olfactory region of RAM 14000, as in juvenile lambeosaurins (ROM 758, 759), is a subdivision of the nasal cavity located rostral to the olfactory bulbs and caudal to the entrance of the main airway to the respiratory region contained within the bulk of the skull (“antorbital region”; Figs. 9C and 9H). In lateral view, the olfactory region is strongly dorsally arched and approximately level with the rostral half of the orbit (Figs. 9B and 9C), as seen in other lambeosaurins for which data are available. In dorsal view (Figs. 9F–9H), the olfactory region is less strongly tapered caudad than in lambeosaurins (ROM 758, 759; CMN 34825).

#### Maxilla

**Figure 12:**
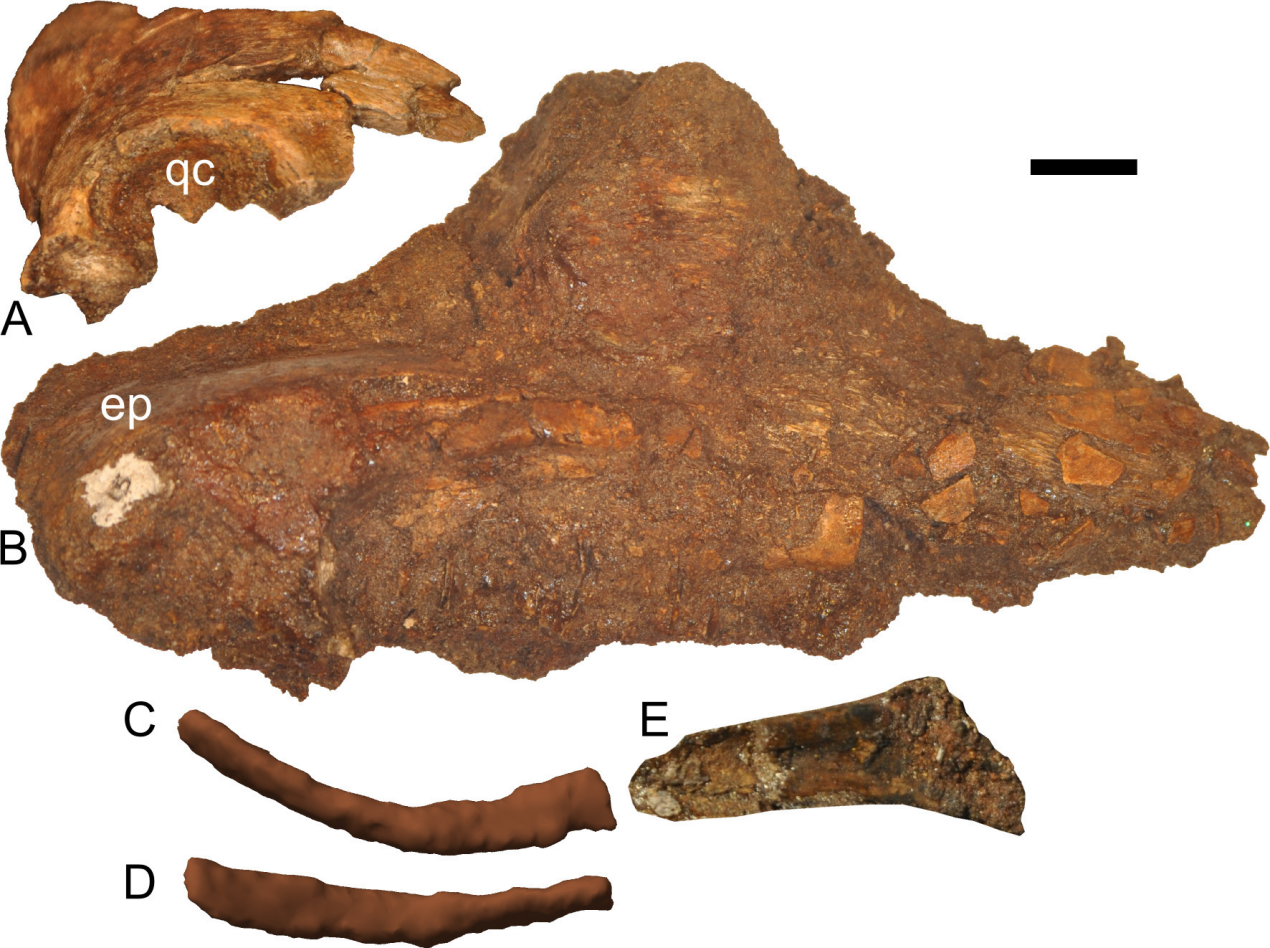
Disarticulated skull elements of *Parasaurolophus* sp., RAM 14000. (A) partial right squamosal in lateral view; (B) right maxilla in lateral view; (C–E) right first ceratobranchial in lateral (C, E) and dorsal (D) views. (C) and (D) are reconstructed from CT scan data. (C) includes the caudal portion of the element, and (E) includes the rostral portion, with the relative position of the two parts approximating their original relationship. Abbreviations: ep, ectopterygoid; qc, quadrate cotyle. Scale bar equals 1 cm.

Like other hadrosaurids, the maxilla is triangular in lateral view (Figs. 7 and 12B), apparently with a straight suture with the premaxilla (unlike some lambeosaurins; e.g., *Hypacrosaurus altispinus*, ROM 702). Fracturing and weathering obscure many additional details.

The prominent ectopterygoid ridge extends from the base of the maxilla’s dorsal process to the caudal edge of the maxilla (Fig. 12B). A marked ventral curvature in the ridge from rostral to caudal corresponds with the shape of the ectopterygoid.

Along the flattened medial surface of the maxilla, a series of alveolar foramina, one between each alveolus, forms a dorsally arched sequence (Fig. 10). A subtle ridge, increasing in prominence caudally, occurs immediately dorsal to the foramina and continues at least for the rostral third of the maxilla; the caudal extent is obscured by fracturing. This morphology can only be evaluated on the left maxilla; the medial surface of the right maxilla is too poorly preserved.

CT scans indicate approximately 20 tooth positions in the maxillary tooth row, with two (rostrally) to three (at mid-point of tooth row) teeth in each file. The greatest internal height of the tooth file is 20 mm at the middle of the bone, and the smallest height is 8 mm at the rostral margin. As exposed on the left maxilla, there were usually two functional teeth on the wear surface at a time. The wear surfaces on each functional tooth range from 4 to 7 mm tall and 3 to 5 mm wide, and the maximum height of the wear surface as exposed at alveolus 5 is 15 mm. Adult *Parasaurolophus* have 40 or more tooth positions in the maxilla (NMMNH P-25100, PMU.R1250; Sullivan & Williamson, 1999), twice the number in RAM 14000. This low tooth count is typical of juvenile hadrosaurids (Suzuki, Weishampel & Minoura, 2004).

#### Jugal

**Figure 13:**
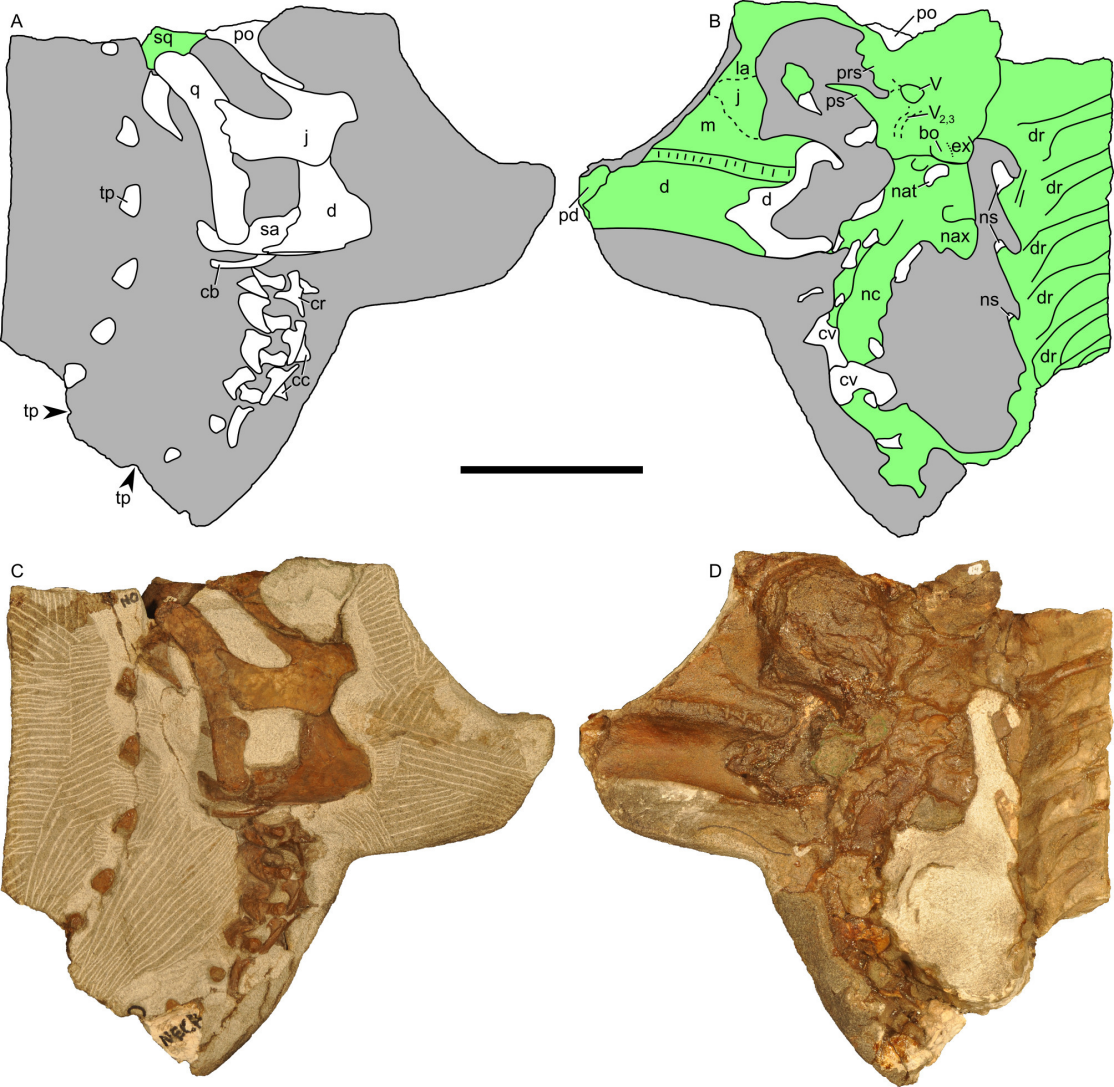
Skull and neck of *Parasaurolophus* sp., RAM 14000. (A) and (C) are in right lateral view; (B) and (D) are a medial view of the same block. (A), (B), interpretive drawings; (C), (D), photographs. Abbreviations: bo, basioccipital; cb, first ceratobranchial; cc, centrum of cervical vertebra; cr, cervical rib; cv, cervical vertebra; d, dentary; dr, dorsal rib; ex, exoccipital; j, jugal; la, lacrimal; m, maxilla; nat, neural arch of atlas; nax, neural spine of axis; nc, neural canal; ns, neural spine; pd, predentary; po, postorbital; prs, presphenoid; ps, parasphenoid; q, quadrate; sa, surangular; sq, squamosal; tp, transverse process; *V*, foramen for CN V; *V*_2,3_, sulcus for CN *V*_2_ and *V*_3_. Bone is shown in white, impressions of bone are shown in green, and rock without bone impressions is shown in gray. Scale bar equals 10 cm.

**Figure 14:**
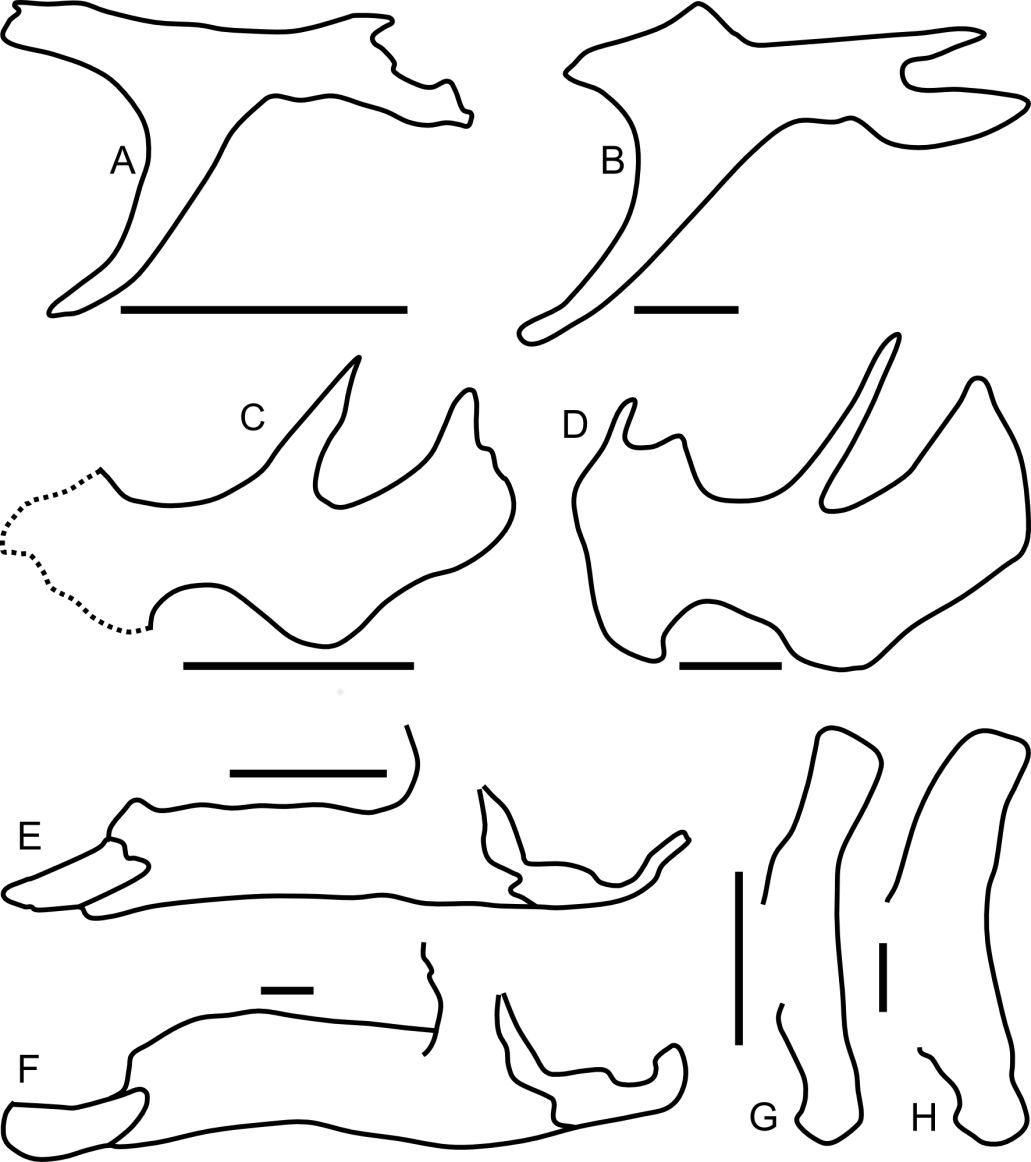
Ontogenetic changes in selected cranial elements of *Parasaurolophus*. Juvenile elements (A, C, E, G) are from RAM 14000; adult elements (B, D, F, H) are from the holotype of *P. walkeri* (ROM 768). All elements are in left lateral view. (A) and (B) postorbital; (C) and (D) jugal; (E) and (F) lower jaw; (G) and (H) quadrate. The jugal in (C) is a composite of the bone preserved on the left side and the impressions of the sutural regions on the right side. *Parasaurolophus walkeri* elements are redrawn and modified after Evans, Reisz & Dupuis (2007). Scale bars equal 5 cm.

Although the left jugal is more complete, crushing obscures the sutures along the rostral margin (Fig. 7). The right side preserves the impressions of these sutures (Figs. 13B and 13D), and the following description is thus a composite of both sides. The jugal forms part of the rostral margin and the entire caudal margin of both the orbit and infratemporal fenestra. The rostral process, along its contact with the maxilla and lacrimal, is triangular and sharply pointed (Fig. 13B). The ventral edge of this rostral process is longer than the dorsal edge, unlike most lambeosaurins of various ontogenetic stages (in which the ventral edge is equal to or shorter in length to the dorsal edge) but similar to the condition in *Parasaurolophus walkeri* (ROM 768; Fig. 14D) as well as a larger juvenile *Parasaurolophus* sp. (SMP VP-1090). A distinct, slightly constricted extension occurs at the rostral end of this rostral process, visible as an impression on the right side, which creates a hooked ventral margin on the process. The ventral and dorsal margins of this rostral process are more acutely angled than seen in adult *P. walkeri* (Figs. 14C and 14D). These shape differences may due, at least in part, to the relatively larger orbit in juveniles. The postorbital process is inclined parallel to the quadratojugal process and tapers along the infratemporal fenestra towards an articulation with the descending process of the postorbital. The quadrate process is tapered and caudodorsally inclined at a 40° angle. Its caudodorsal edge is exceptionally pointed compared to other lambeosaurines, and is not expanded relative to the rest of the process as in *Kazaklambia convincens*. The jugal is dorsoventrally constricted ventral to the orbit (19 mm tall) and on the quadrate process ventral to the infratemporal fenestra (22 mm tall). Similar constrictions are also seen in *Corythosaurus*, *Lambeosaurus*, and other *Parasaurolophus* (Evans, 2010). The angle between the postorbital and quadrate processes is quite tight, similar to the condition in *Hypacrosaurus*, *Parasaurolophus*, and *Kazaklambia convincens* (Bell & Brink, in press; Rozhdestvensky, 1968). As preserved, the jugal forms only the ventral third and quarter of the rostral and caudal margins of the infratemporal fenestra, respectively (Figs. 7 and 13).

#### Quadrate

The quadrate is complete on both sides, but the right quadrate is slightly displaced ventrally and both quadrates are slightly displaced laterally. The quadrate forms the caudal margins of the infratemporal fenestra and the skull (Figs. 7 and 13). The dorsal condyle of the quadrate articulates with the squamosal cotyle, as is typical of hadrosaurids. Dorsal to its contact with the jugal, the quadrate is slightly concave caudally and is inclined caudodorsally at 30° relative to vertical. The ventral third of the quadrate is straight. The surface for articulation with the caudal process of the jugal is rostrally bifurcated, resulting in an S-shaped sutural surface (Fig. 7); the dorsal half of the quadrate tapers along the infratemporal fenestra towards this articulation. The dorsal condyle of the quadrate is triangular (with a rounded and medially directed apex) in dorsal view, whereas it is rounded in lateral view. The ventral end is rounded in lateral view and trapezoidal with a saddle-shaped articular surface in ventral view. The ventral condyle of the quadrate is 21.4 mm wide and 18.2 mm long on its lateral edge and 8.7 mm long on its medial edge, respectively. In caudal view, the quadrate is straight but slightly bowed medially (Figs. 8F and 8G). The quadrate articulates with the pterygoid wing rostromedially along a V-shaped suture, extending from the quadratojugal to the dorsal margin of the infratemporal fenestra (Fig. 10). The pterygoid flange of the quadrate is only partially preserved, forming a plate-like and slightly concave (in medial view) region of bone (Fig. 10). At its ventral third, the caudal edge of the quadrate is flattened; dorsally, the element’s caudal edge tapers to a rounded ridge. The quadrate in RAM 14000 is more gracile than seen in adult *Parasaurolophus* (Figs. 14G and 14H).

#### Quadratojugal

The quadratojugal is not visible on the left side, but CT scans indicate that the rest of the element is displaced rostromedially relative to the jugal. The quadratojugal is a thin, sinuous and rostrally inclined element that rostrodorsally tapers to a point and buttresses the quadrate caudoventrally.

#### Squamosal

The squamosal is thin and arched dorsally, with a concave quadrate cotyle on its ventrolateral surface (Fig. 12A). The prequadratic process is sharply pointed rostrodorsally. The postquadratic process has a straight rostral border and a convex caudal border that abuts the paroccipital process (Fig. 7). The squamosal forms the caudolateral margin of the supratemporal fenestra and the dorsal margin of the infratemporal fenestra. Measuring from its edge on the base of the paroccipital process to the dorsal margin of the squamosal, the element is 67 mm tall. The caudomedial corner of the squamosal hooks upward in lateral view, and the dorsal surface of the squamosal is entirely convex. The medial extents of the squamosals are not preserved, so we cannot determine if they contacted each other as in most lambeosaurins, *Kazaklambia convincens* and adult *Parasaurolophus*, or were separated by the parietals as in *Velafrons* (Bell & Brink, in press; Gates et al., 2007; Brink et al., 2011).

#### Lacrimal

The lacrimal forms the mid-rostral margin of the orbit. Sutures with the prefrontal are difficult to interpret, as are those with the premaxilla. Impressions on the right side (Fig. 13B) show that the lacrimal articulates ventrally with the jugal along a caudoventrally inclined, slightly ventrally convex suture.

#### Postorbital

The postorbital is T-shaped in lateral view (Figs. 7 and 14A), bounding part of the dorsal margin of the orbit and nearly the entire caudal margin as well. The postorbital articulates with the prefrontal rostromedially along a straight suture and the frontal medially along a more sinuous suture (Fig. 8D). The jugal process is slightly curved rostrally and forms most of the rostrodorsal margin of the infratemporal fenestra, tapering alongside the caudal towards articulation with the ascending process of the jugal. The caudal process of the postorbital measures 13 mm wide at its narrowest point, but broadens caudally. The caudal-most portion of the caudal process thins and splits into dorsal and ventral prongs (Fig. 7A), as in *Parasaurolophus* and lambeosaurins except for *Hypacrosaurus altispinus* (Evans, 2010); the ventral prong is more extensive. This process overlaps the dorsal surface of the squamosal, and forms a small part of the rostrolateral margin of the supratemporal fenestra. In lateral view, the dorsal edge of the postorbital is slightly concave, unlike the convex margin in *P. walkeri* (ROM 768). The maximum length of the jugal and caudal processes are roughly equal, similar to lambeosaurins of various sizes, but unlike adult *Parasaurolophus* (where the jugal process is longer; NMMNH P-25100, ROM 768) or *Charonosaurus* (where the caudal process is longer). Similarly, the rostral process of the postorbital is much shorter in adult *Parasaurolophus* (e.g., ROM 768, Fig. 14B) than in RAM 14000. Consequently, the proportion of the skull roof in RAM 14000 formed by the postorbital is much greater than that formed by the squamosal in lateral view (Fig. 7A), unlike adult *Parasaurolophus*. Unlike *Kazaklambia convincens* or *Charonosaurus jiayinensis* (Bell & Brink, in press), the postorbital lacks a dome on its rostral process in RAM 14000.

#### Frontal

The left frontal is nearly completely preserved with visible sutures, except for its extreme caudomedial portion (Figs. 8C and 8D). In dorsal view, the frontal articulates with the prefrontal rostrolaterally along a linear suture that trends laterally along its caudal extent. The suture with the postorbital is comparatively linear also, with a slight medial trend from rostral to caudal. The contact with the parietal is obscured, but a small portion of the frontal’s contribution to the supratemporal fenestra is visible. The paired nasals form a triangular prong that laps onto the rostral end of the dorsal surface of the frontals (Fig. 8D). This morphology is unique relative to the rounded or squared contact in lambeosaurins and adult *Parasaurolophus*, where the sutures can be determined (Evans, Reisz & Dupuis, 2007; Brink et al., 2011). It also differs from *Kazaklambia convincens*, where a prong of the paired frontals inserts between the nasals on the midline (Bell & Brink, in press). Adult and subadult *Parasaurolophus* have a nasofrontal suture that is expanded caudodorsally and sharply angled relative to the rest of the skull roof (Evans & Reisz, 2007); there is no evidence in CT scan or direct visual observation of such a feature in RAM 14000. Thus, the condition here is comparable to the non-angled and unexpanded state in lambeosaurin juveniles and adults, as well as the condition in *K. convincens*. Similarly, the individual frontal in RAM 14000 is approximately as long at the midline (measuring from the caudal extent of the nasal suture to the rostral extent of the parietal suture) as it is wide (34.2 mm vs. 31.7 mm, a ratio of 1.08; doubling to approximate the width across both frontals produces a ratio of 0.54). The median frontal dome is thus fairly elongate (Fig. 7). This too contrasts with the condition in adult and subadult *Parasaurolophus* (where the frontal is wider than long) and is more similar to the state in lambeosaurins of various growth stages (Evans, Reisz & Dupuis, 2007). Similar to other lambeosaurines, the frontal does not reach the orbital rim.

#### Prefrontal

Only the sutures on the caudal edge of the left prefrontal are clearly visible (Figs. 7, 8C and 8D). Here, the bone forms a triangular point interposed between the medial margin of the postorbital and the lateral margin of the frontal, as in other lambeosaurines. The bone forms the rostrodorsal margin of the orbit and contacts the lacrimal ventrally. Based on the extent of the premaxilla, it is unlikely that the prefrontal formed any significant portion of the crest in RAM 14000 (unlike adult lambeosaurines but similar to many subadult specimens; Evans, Forster & Reisz, 2005).

#### Ectopterygoid

The ectopterygoid sits atop the caudodorsal margin of the caudal process of the maxilla, extending medial to the coronoid as viewed on CT scans. The element is best-preserved on the right side (Fig. 12B), showing that the ectopterygoid is a thin and broad element with a prominent ventral bend at its caudal third. The mediolateral width of the ectopterygoid and its relationship to structures such as the pterygoid cannot be visualized because of weathering.

#### Pterygoid

The pterygoid is visible only on the left side (Fig. 10), with just its caudal quadrate wing preserved. The wing is thin (<1 mm) and gently concave medially, paralleling the corresponding medial surface of the quadrate ramus. As viewed in CT scan, the nearly complete pterygoid on the right side is typical of the condition expected for hadrosaurids (Ostrom, 1961; Heaton, 1972).

#### Palatine

The palatine is not sufficiently preserved or exposed to comment upon its morphology.

#### Vomer

The caudodorsal portion of the vomer is exposed on the left half of the skull (Fig. 10). The preserved dorsal edge is acutely angled, and the rostral edge of the element tapers rostrolaterally towards its (inferred) insertion between the premaxillae. The apex of the vomer is located just rostral to the rostral end of the orbit, at approximately the same height (dorso-ventral level). The vomer is not sufficiently preserved for detailed comparison with the element in other hadrosaurids.

#### Braincase

**Figure 15:**
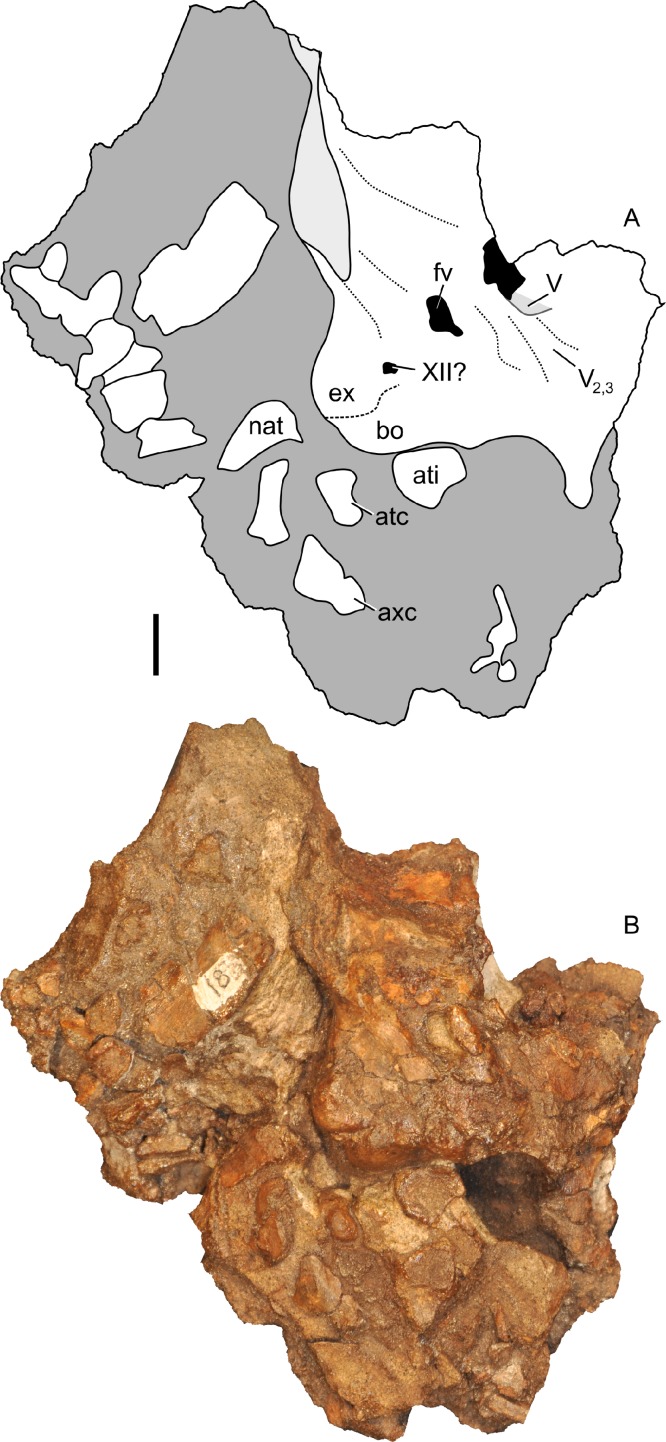
Partial braincase of *Parasaurolophus* sp., RAM 14000, in right lateral view. (A) interpretive drawing; (B) photograph. Abbreviations: atc, atlas centrum (odontoid); ati, atlas intercentrum; axc, axis centrum; bo, basioccipital; ex, exoccipital; fv, foramen vestibuli; nat, neural arch of atlas; XII?, foramen tentatively identified as that for CN XII; V, foramen for CN V; *V*_2,3_, sulcus for CN *V*_2_ and *V*_3_. Bone is shown in white, broken bone surface is shown in light gray, and matrix is shown in dark gray. Unlabeled bones are not confidently identified, but may represent vertebral fragments. Scale bar equals 1 cm.

Most of the braincase was partially disarticulated from the rest of the skull by weathering, and the right side was prepared out to show relevant details (Fig. 15). Additional features are seen as impressions on the right skull block (Figs. 13B and 13D). This section describes only visible features. Additional internal details were reconstructed from CT scans and are described in the section on the endocast. With the exception of the sutures between the exoccipital and basioccipital on the occipital condyle, sutures within the braincase are not visible due to crushing, weathering, and fusion.

The parasphenoid, represented by an impression, is 28 mm long, gently arched along its length, and tapered to a point at its rostral end (Figs. 13B and 13D). It terminates just caudal to the midpoint of the orbit. A shallow sulcus occupies the lateral surface of the bone. Faint impressions tentatively identified as presphenoid occur dorsal to the parasphenoid, but no notable details are visible. The form is generally similar to that seen in *P. tubicen* (NMMNH P-25100, PMU.R1250).

A foramen interpreted as that for cranial nerve XII (hypoglossal nerve) is small (1.9 by 2.2 mm) and located roughly midway between the caudal extent of the occipital condyle and a ridge of bone that slants caudodorsally along the braincase (Fig. 15). Additional foramina may have occurred also, as in *Hypacrosaurus altispinus* (Evans, 2010), but cannot be confirmed in the specimen’s current state of preparation and preservation.

A portion of the trigeminal foramen is exposed at the front of the right side of the isolated braincase (Fig. 15), and the remainder of the impression is seen on the right skull block (Figs. 13B and 13D). This impression is triangular, measuring 11 mm long and 9 mm tall. Two distinct grooves (ridges on the natural mold) extend from the foramen; one trends directly rostrally from the rostral edge of the foramen (probably representing the path for CN *V*_1_), and the other trends rostroventrally from the ventral edge (representing the path for CN *V*_2,3_).

The left caudal semicircular canal is exposed through a fortuitous break (Fig. 10). The maximum diameter of its lumen is 1.8 mm.

The occipital condyle is roughly cardoid in caudal view, composed of the basioccipital at the ventral and ventrolateral edges and the exoccipitals at the dorsolateral edges (Fig. 15). All three elements are bulbous on their caudal edges. The rounded basal tuberosity has its maximum lateral extent slightly lateral to the extreme edge of the occipital condyle. In lateral view, the exoccipitals rise to bound the exposed portion of the foramen magnum, sweeping dorsally.

The exoccipital and opisthotic are fused both in gross examination and CT scans. The most prominent and best-preserved aspect of these elements is the paroccipital process, which curves rostrally and tapers dorsoventrally along the caudal margin of the paroccipital process and upper squamosal (Figs. 7, 8F and 8G). The caudal surface of the bones is remarkably flat, with only a slight concavity at its distal extent (Figs. 8F and 8G). The fenestra vestibuli (fenestra ovalis) measures approximately 5 mm tall by 2.6 mm long. The auditory recess is deepest and narrowest by the fenestra vestibuli, becoming broader and shallower dorsocaudally (Fig. 15).

#### Dentary

The ramus of the left dentary has an average height of 27 mm. The edentulous process is roughly 25 percent of the dentary’s length, and the rostral border of the process is rostroventrally inclined (Fig. 7). The ventral border of the dentary is relatively straight, with comparatively little declination at its rostral portion. This is comparable to the morphology in *Parasaurolophus walkeri* (Fig. 14F, ROM 768; Evans, 2010), but different from the more inclined morphology in *P. tubicen* (NMMNH P-25100), a dentary from the Fruitland Formation tentatively identified as juvenile *Parasaurolophus* sp. (SMP VP-1090; Sullivan & Bennett, 2000), and other lambeosaurines. The condition in *P. cyrtocristatus* is unknown.

The lateral surface of the body of the dentary is strongly convex (Fig. 7). The coronoid process is perpendicular to the ventral margin of the dentary, and, based on CT scans and the incomplete dentary on the right half of the skull (Figs. 13B and 13D), reaches the ventral margin of the orbit when in articulation, roughly 72 mm above the ventral margin of the dentary. The rostral margin of the coronoid process is more prominently extended than the caudal process. Rostrally, the dentary tapers to articulate with the caudal margin of the predentary. Caudally, the dentary articulates with the surangular along a sinuous suture (Fig. 7). The number of dentary teeth cannot be determined.

#### Predentary

Only the left side of the predentary is preserved (Figs. 7, 8A, 8B and 14E), but the element can be mirrored to reconstruct the overall shape. In dorsal view, the element would have been roughly horseshoe-shaped, with a moderately convex rostral margin. As exposed at the midline, the cross-section of the rostral portion is approximately triangular (Fig. 10). The dorsal triturating surface is approximately 14 mm long and only slightly rostrally inclined. This inclination becomes more extreme towards the lateral and caudal wings of the predentary, so that the triturating surface is nearly vertical and laterally facing (14 mm tall) at its caudal end. Thus, the surface only changes its orientation and not its width. The ventral surface of the predentary is gently convex. The caudal edge of the lateral wing of the predentary is forked; the ventral process of this fork is slightly longer and more sharply pointed (Fig. 7). This is in contrast to the unforked lateral wing in the holotype of *P. walkeri*, ROM 768 (Fig. 14F), but similar to the condition in other lambeosaurines. The morphology is not known in other species of *Parasaurolophus*. The median process of the predentary is not definitively preserved in RAM 14000.

#### Surangular

The surangular (Figs. 7, 13A, 13C and 14E) buttresses the caudal margin of the coronoid process, with a smoothly continuous lateral surface at this point. A ridge at the base of the contribution to the coronoid process continues onto the lateral edge of the articular surface for the quadrate. This coronoid process is also relatively broader than in ROM 768 or NMMNH P-25100. The surangular’s ventral margin is slightly convex, with a strong curvature caudally on the articular process. The surangular receives the ventral condyle of the quadrate and articulates with the angular caudomedially. The retroarticular process of the surangular is thinner and more horizontal than in *P. walkeri* (ROM 768, Fig. 14F).

#### Angular

The angular is a flattened bone that curves caudodorsally and articulates medially with the surangular. On both sides, the element has been displaced downward so that its ventral margins are visible beyond that of the surangular (Figs. 7, 13A and 13C). It is inferred to receive the distal end of the quadrate. In ventral view, the element is long and narrow.

#### Hyoid

A bone interpreted as the caudal end of the first ceratobranchial is positioned immediately ventral to the surangular (Figs. 7, 13A and 13C); the right first ceratobranchial is slightly better preserved than the left. The element is partially exposed, and described from gross examination as well as CT scan reconstructions (Figs. 12C–12E). Although the ceratobranchials of hadrosaurids (including *Hypacrosaurus sternbergii*, adults of *Saurolophus osborni*, *Lambeosaurus lambei*, and *Corythosaurus casuarius*, as well as juveniles of *Hypacrosaurus altispinus* and *H. stebingeri*) previously have been described as generally uniform (Ostrom, 1961; Gates et al., 2007; Brink et al., 2011), the morphology of these elements in RAM 14000 has some unique aspects. These differences may be taxonomic or perhaps ontogenetic. However, the hyoids of embryonic *H. stebingeri* (RTMP 89.79.52) are quite similar to those of *Corythosaurus* in major details, so we posit that taxonomic differences are most influential here. The articulated preserved portion of each ceratobranchial in RAM 14000 is gently arched ventrally, with a slight dorsoventral curvature. The caudal portion is dorsoventrally flattened (rather than cylindrical, as described for other hadrosaurids; Ostrom, 1961). Rostrally, the bone twists so that it is mediolaterally compressed at the rostral-most preserved portion. This caudal portion is approximately 43 mm long, as preserved. The rostral end of the right first ceratobranchial is within a disarticulated block of matrix; impressions of surrounding elements permit confident placement of the bone. The part that connects with the rest of the ceratobranchial is missing, but the preserved portion in this separate block (including a partial impression) is 37 mm long. The impression is 8 mm tall at narrowest, 14 mm tall at its rostral end, and only 3.5 mm thick mediolaterally. Such extremely expanded rostral ends are typical of known hadrosaurid ceratobranchials (Ostrom, 1961). Including both portions, the total ceratobranchial length was at least 80 mm. The rostral end of the left first ceratobranchial was displaced by erosion (Fig. 10), and is similar in overall morphology to the element on the right.

#### Endocast

**Figure 16:**
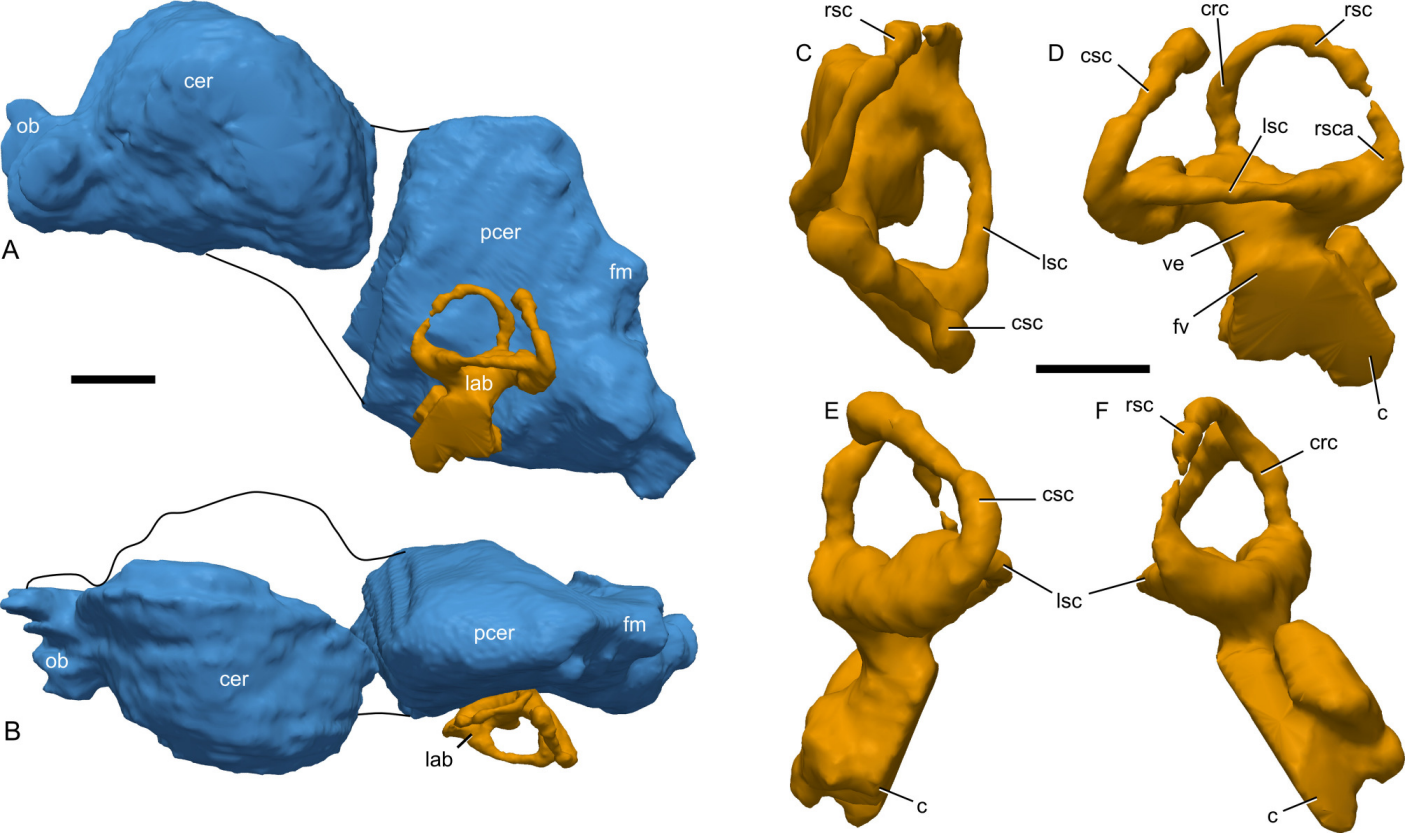
Cranial endocast of *Parasaurolophus* sp., RAM 14000, reconstructed from CT scans. (A) left lateral view of entire endocast; (B) dorsal view of entire endocast. The rostral and caudal portions were disarticulated, and thus their relative positions (joined by the black lines) should be considered tentative. The caudal portion was mirrored to match the rostral portion. (C–F), endosseous labyrinth of right inner ear. (C) dorsal view; (D) lateral view; (E) caudal view; (F) rostral view. The endocast of the brain is colored blue and the endocast of the bony labyrinth is colored orange. Abbreviations: c, cochlear duct; cer, cerebrum; crc, crus communis; csc, caudal semicircular canal; fm, endocast of foramen magnum; fv, fenestra vestibulae (approximate location); lab, endosseous labyrinth; lsc, lateral semicircular canal; ob, olfactory bulbs; pcer, postcerebral region; rsc, rostral semicircular canal; rsca, ampulla of rostral semicircular canal; ve, vestibule. Scale bar at left is for (A) and (B) and equals 1 cm. Scale bar at right is for (C–F) and equals 5 mm. Because of differences in perspective between images, the scale bar is only approximate.

A partial cranial endocast for RAM 14000 was reconstructed from CT scan data (Fig. 16, Figs. S1 and S2), the first ever for *Parasaurolophus* of any ontogenetic stage. The endocast was reconstructed in two sections, one on the portion of the braincase articulated with the left half of the skull (Fig. S1) and the remainder on the disarticulated portion of the braincase (Fig. S2). Their relative position was then approximated based on cranial landmarks and comparison with other hadrosaurids. Because of weathering, many of the smaller neurovascular canals and foramina could not be discerned with confidence.

The overall shape of the endocast is broadly similar to that previously described for juvenile and adult lambeosaurines (Evans, 2006; Evans, Ridgely & Witmer, 2009). In dorsal view, the cerebrum is strongly expanded laterally (Fig. 16B), with an estimated width across the midline of 36 mm, dorsoventral height (perpendicular to the aforementioned width) of 28 mm, and an estimated cerebral length of 39 mm. In lateral view (Fig. 16A), the cerebrum is very strongly arched, much more so than in larger juvenile (*Lambeosaurus* sp., ROM 758; *Corythosaurus* sp., ROM 759), subadult *(Corythosaurus* sp., CMN 34825), or adult (*Hypacrosaurus altispinus*, ROM 702) lambeosaurins. This may in part be due to the young ontogenetic status of RAM 14000, in that the frontals (and hence cerebra) are more strongly arched in young individuals (e.g., *Hypacrosaurus stebingeri*, MOR 548). An opposite trend in cerebral morphology may occur in *Alligator mississippiensis*, in that the cerebral region of the endocast is less strongly arched in hatchlings than in adults (e.g., the hatchling OUVC 10606 versus the adult OUVC 9761; A Farke, personal observation).

The olfactory bulb endocast is a maximum of 14 mm across. As reconstructed, the olfactory bulbs are approximately half the thickness of the cerebrum in lateral view, and are depressed considerably below the cranial roof (frontal, in this case), particularly at their origin (Figs. 16A and 16B).

Angulation between the cerebrum and postcerebral region (equivalent to “cephalic flexure” as measured in endocasts of the ornithopod *Dysalotosaurus lettowvorbecki* by Lautenschlager & Hübner, 2013) cannot be determined with confidence, so any taxonomic or ontogenetic comparisons of this region cannot be conducted. The postcerebral region (a term used here because the cerebellum itself is not well distinguished in hadrosaurid endocasts; Evans, 2006) is much narrower and deeper than the cerebrum (Figs. 16A and 16B). The ventral margin is broadly rounded in lateral view, contrasting with the straighter margin seen in lambeosaurins (Evans, Ridgely & Witmer, 2009; Fig. 7). The dorsal margin of this region, equivalent to the dural peak of Lautenschlager & Hübner (2013; Fig. 2) is much more sharply angled (approximately 90°) than in larger lambeosaurins (e.g., around 120° in subadult *Corythosaurus* sp., CMN 34825). The angulation (but not the prominence) of the dural peak is mostly unchanged through known ontogenetic stages of the small ornithopod *Dysalotosaurus lettowvorbecki* (Lautenschlager & Hübner, 2013), so we hypothesize that phylogenetic differences between lambeosaurins and parasaurolophins explain these differences among hadrosaurids.

The endosseous labyrinth is best-preserved on the right side, although not all aspects of the labyrinth could be traced continuously on the CT scan data (Figs. 16C–16F and Fig. S2). The rostral semicircular canal is only slightly taller than the caudal semicircular canal (when the lateral canal is oriented horizontally; Fig. 16D), a less marked size disparity than seen in ontogenetically older lambeosaurins (Evans, Ridgely & Witmer, 2009; Fig. 8). We estimate the maximum breadth of the rostral canal at 11 mm (from ampulla to crus communis), and that for the caudal canal at 10.5 mm (from ampulla to crus communis). Between its bounding ampullae, the lateral semicircular canal spans approximately 9 mm (Fig. 16C). The rostral canal has the tightest arch whereas the caudal canal is broadest, and the lateral semicircular canal is the smallest. The lateral ampulla is the largest of the three. From the foramen vestibuli, cochlea is estimated to be approximately 7.6 mm long. Note that the ventral margins of the cochlea are poorly visible on the CT scans (Fig. 16), and thus this measurement should be considered only an approximation. The endolymphatic duct is not clearly visible. Overall, the morphology of the endosseous labyrinth is broadly similar to that described for other hadrosaurids (e.g., Ostrom, 1961; Evans, Ridgely & Witmer, 2009).

#### Stapes

The left stapes of RAM 14000 is immediately caudal to the quadrate and pterygoid and rostral to the paroccipital-opisthotic process (Fig. 10), consistent with the position in adult *Corythosaurus casuarius* AMNH 5338 (Colbert & Ostrom, 1958). The proximal end of the stapes is presumed missing, probably due to post-depositional separation of the braincase. The remaining structure suggests that the stapes was a cylindrical, rod-like element. The bone is slightly bent mediolaterally, probably from taphonomic deformation. The maximum preserved length is 12.5 mm, whereas the maximum width is between 0.6 and 0.8 mm (with the widest portion proximally; i.e., towards the braincase), suggesting a slight tapering of the bone laterally (away from the braincase). The distal end of the stapes is positioned 8 mm dorsal to the ventral tip of the paroccipital-opisthotic process.

### Postcranial axial skeleton

#### Vertebrae

The vertebral column is poorly preserved, with many details obscured by fragmentation of the bones and matrix. Thus, the following description is necessarily incomplete. We are unable to evaluate neurocentral fusion in any vertebrae, although we do note that the sacral ribs are not fused to the sacral vertebrae. Measurements are presented in Table 6.

The complete count of cervical vertebrae cannot be determined, because the most caudally placed cervicals are missing (Fig. 13). Individual components of the atlas are unfused and partially exposed (Figs. 13 and 15). The atlas intercentrum, as exposed, is triangular in cross-section, with a sharp ventral keel (Fig. 15). Its caudal, dorsal, and cranial edges are not exposed, so the nature of their articulations is not known. The odontoid (atlas centrum) is partially exposed and globulose, showing a convex cranial margin and a concave caudal margin (Fig. 15). The fragmentary neural arch for the atlas shows no remarkably morphology. An impression of the neural spine of the axis (C2; Figs. 13B and 13D) shows the element to be tall (∼26 mm) and elongate (17 mm long at its base). The morphologies of both the atlas and axis broadly agree with those previously described for *Gryposaurus incurvimanus* (Parks, 1920), although the preservation in RAM 14000 is not sufficient to compare any details, nor has adequate comparative material been illustrated or described for other lambeosaurines.

In the cervicals for which a centrum is preserved (?C4–?C7), the centrum is strongly opisthocoelous and sharply pinched in mediolateral cross-section, with a strong ridge on the lateral surface of the centrum (Figs. 13A and 13C). The dorsal edge of the lamina connecting the zygapophyses is strongly arched, and the diapophyses in the middle of the cervical vertebral series project laterally with a ventral inclination from medial to lateral. The tips of the diapophyses are at approximately the upper half of the centrum. The vertebrae themselves are partly eroded, so little more can be said about their morphology. In both proportions and overall shape, the preserved cervical vertebrae appear similar to comparable elements in the adult *P. walkeri* ROM 767 (Parks, 1922).

**Figure 17:**
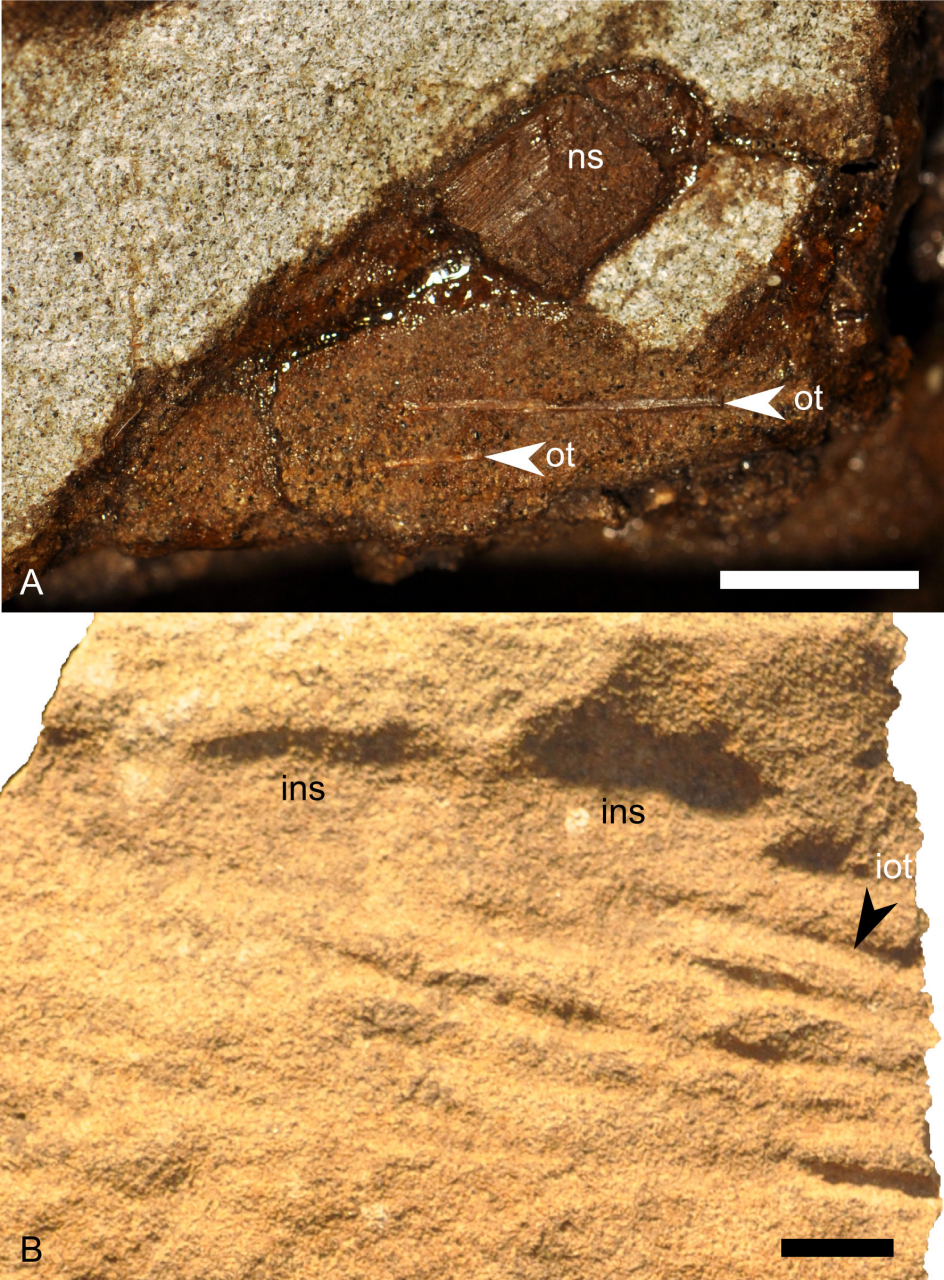
Ossified tendons of *Parasaurolophus* sp., RAM 14000. (A) ossified tendons associated with cranial dorsal vertebra (?D4), with medial surface of tendons visible (cranial end is to left of image); (B) impressions of ossified tendons associated with either caudally placed dorsal vertebrae or cranially placed sacral vertebrae (cranial end is to right of image). Abbreviations: ins, impression of neural spine; iot, impression of ossified tendon; ns, neural spine; ot, ossified tendon. Scale bars equal 1 cm.

The dorsal vertebrae are poorly preserved (Figs. 2, 3 and 13). There were at least 17 dorsal vertebrae (determined by counting the centra, exposed transverse processes, and ribs), but the exact count is unknown. Impressions of the neural spines for three cranial dorsals show the spines to be strongly caudally inclined, mediolaterally compressed, and craniocaudally narrow (Figs. 13B, 13D and 17A), contrasting with the more robust (craniocaudally elongated) spines in adult lamebosaurines (e.g., *P. walkeri*, ROM 767; *Corythosaurus casuarius*, AMNH 5338). The associated transverse spines for these vertebrae are triangular in lateral view and rounded along their lateral extrema (Figs. 13A and 13C). Centra are visible (but poorly preserved) only for the caudal dorsals; here, the centra are slightly taller than long.

The sacrum is neither well exposed nor well-preserved. The fragmentary neural spines are erect and straight (Fig. 3), similar to the condition in adult *Parasaurolophus* spp. (e.g., FMNH P 27393, ROM 767; A Farke, personal observation).

The caudal vertebrae are best exposed on the left side (Fig. 3) but are poorly preserved. As articulated, the series of caudal centra is gently arched, with an overall ventral concavity along its margin. In contrast with the neural spines of the sacral vertebrae, the preserved neural spines of the cranial caudal vertebrae are distinctly curved. The proximal portion (nearest the neural arch) projects caudally at approximately 45° and then curves dorsally at its distal two-thirds. Thus, the cranial margin of the neural spine is concave and the caudal margin convex. The neural spines at the cranial end of the tail are quite tall relative to the centra, as is typical for hadrosaurids. Moving distally along the tail, the neural spines lose their curvature by approximately caudal 8. This contrasts with adult *P. cyrtocristatus*, in which the neural spines maintain their curvature at least through the middle section of the tail (Ostrom, 1963), but is similar to adult *P. walkeri* (ROM 767). The transverse processes are most pronounced in the cranial caudal vertebrae, becoming successively less prominent distally. By caudal 13 or 14, the transverse processes are gone. A total of 19 centra are visible, and they exhibit the typical hexagonal shape of hadrosaurids. Assuming a typical caudal vertebral count and proportion of the tail for lambeosaurines (see data in Lull & Wright, 1942), just under half of the physical length of the tail is preserved in RAM 14000, and perhaps another 30 to 40 additional vertebrae are missing.

#### Ribs

Cervical ribs are visible on the fourth through seventh cervical vertebrae (Figs. 13A and 13C). The fourth cervical rib is tripartite, with a rounded capitulum and a tab-like tuberculum of approximately the same size. The shaft of the rib is short and triangular in cross-section, with a distinct flange on its lateral surface. This flange terminates in a discrete knob on the lateral surface of the proximal end of the rib, equidistant between the capitulum and tuberculum. Both the ridge and the knob become less pronounced on the successive two cervical ribs (with C5 and C6) and are entirely absent by C7. Similarly, the tuberculum becomes successively less pronounced relative to the capitulum on the ribs associated with C5 and C6. All of the preserved cervical ribs are short, no longer than the centra of their associated vertebrae. In general, the cervical ribs of RAM 14000 are less expanded distally than seen in adult *P. walkeri*, ROM 768 (Parks, 1922; plate VII, Fig. 1). In this specimen, the distal ends are expanded so as to be somewhat paddle-shaped.

Thirteen dorsal ribs are preserved with RAM 14000 (Figs. 2 and 13); some of the more caudally placed ribs may be missing or unexposed, which may explain the discrepency from the count of 17 dorsal ribs in adult *P. walkeri* (Parks, 1922). The first three ribs drastically increase in size successively, and the third rib is the longest by far. The fourth through seventh ribs are approximately the same length, with a drastic, successive decrease in size for the eighth through eleventh dorsal ribs. The twelfth and thirteenth dorsal ribs are approximately the same size. By contrast, the successive elongation of dorsal ribs in adult *P. walkeri* (ROM 767) continues through the fifth rib, and the successive shortening of dorsal ribs commences at position nine. This may reflect ontogenetic or potentially species-level differences. The preserved portions of the first two ribs in RAM 14000 show that their shafts are quite straight in lateral and cranial view. The third through tenth ribs are straight in lateral view but have a gentle medial concavity. The eleventh through thirteenth ribs are once again straight in lateral and cranial view. As a consequence of the flexion of the dorsal vertebrae, the first through eighth ribs converge upon each other distally. The ninth and tenth ribs are less convergent along their shafts, but appear to be mostly in natural position. The eleventh through thirteenth ribs are slightly disarticulated on the right (up) side, suggesting disturbance from scavengers or water currents prior to burial. The ribs on the left side are too fragmented to evaluate their anatomy.

The first and second sacral ribs are visible on the right side (Fig. 2), with the first better exposed. The following description focuses on the first sacral rib. As with the caudal dorsal ribs, this sacral rib is slightly out of articulation. Its proximal end is strongly flared; the capitulum and tuberculum are connected by a thin web of bone. The distal termination of the rib flares slightly relative to the shaft, at approximately 17 mm wide. Both dorsal and ventral borders of the rib are concave, with the dorsal border more strongly so.

Measurements for the ribs are presented in Table 7.

#### Haemal arches

The haemal arches are fragmented. Only one cranial arch (perhaps with Ca6?) is sufficiently preserved for description (Fig. 3). This element is exposed along its lateral surface only, and the shaft of the bone is straight.

### Appendicular skeleton

#### Pectoral girdle

The blades of both scapulae are preserved (Figs. 2 and 3), with the left more complete. Measurements are presented in Table 8. The scapular neck has a distinct constriction cranially, typical of lambeosaurines. However, the caudal expansion of the blade is less pronounced than in adult *Parasaurolophus* (FMNH P 27393, ROM 768). Overall, the scapula is more robust than seen in *Corythosaurus*, *Lambeosaurus*, or *Nipponosaurus* (Suzuki, Weishampel & Minoura, 2004). The preserved ventral border of the left scapula is entirely intact in RAM 14000, whereas the dorsal border is less complete. Only the ventral border is intact on the right side; here, it is slightly sinuous, with a distinct constriction at the cranial third of the element. No sternal elements are preserved.

#### Pelvic girdle

The pubes are somewhat fragmented, but the general shape of the prepubic process (pubic blade) is intact (Figs. 2 and 3). The cranial end is dorsoventrally expanded (as is typical of lambeosaurines), with the ventral margin slightly more extended cranially than the dorsal margin. The blade narrows caudally. A short segment of the postpubic rod is exposed (Fig. 2), showing that this was a thin process with morphology typical of lambeosaurines.

The right ischium is represented by bone proximally and by impressions distally (Fig. 3). The dorsal acetabular process is longer and broader than the ventral, as seen in other hadrosaurids. The impression of the shaft is comparatively straight, showing that the shaft expands dorsoventrally towards its distal end. Unlike adult-sized *Parasaurolophus cyrtocristatus* (FMNH P 27393), the distal extremity of the ischium is not prominently hooked cranially. At most, there was only a slight expansion. A similar expansion of the distal hook during ontogeny occurs in *Hypacrosaurus stebingeri* (Horner & Currie, 1994), but in this taxon the hook develops at a comparatively smaller body size than that of RAM 14000.

**Figure 18:**
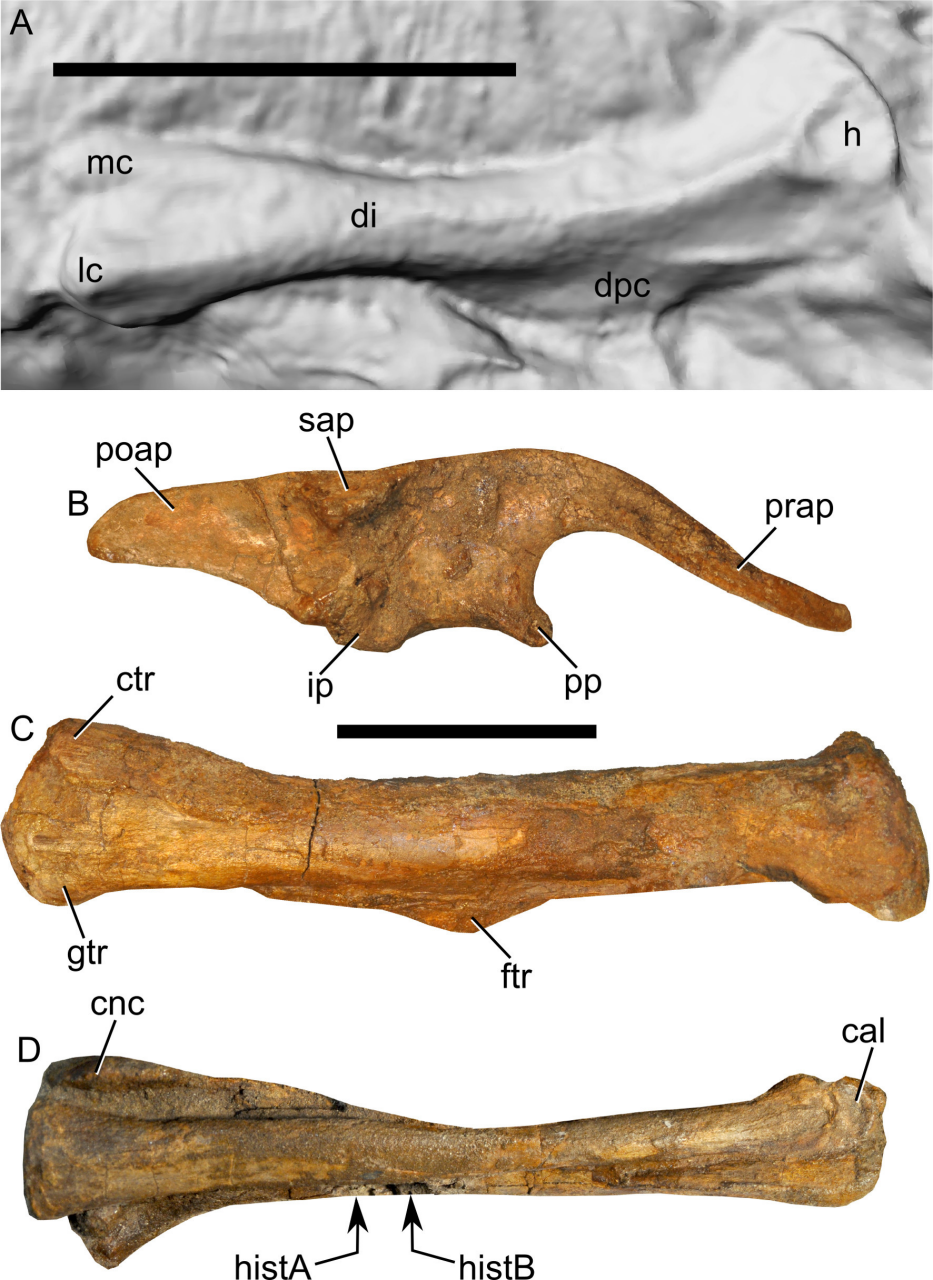
Major limb bones from the right side of *Parasaurolophus* sp., RAM 14000, in lateral view. (A) humerus; (B) ilium; (C) femur; (D) tibia and fibula. The image in (A) is a digital surface model generated from photogrammetric reconstruction of the natural mold. The head appears unusually flat because some bone still fills that area. Abbreviations: cal, calcaneum; cnc, cnemial crest of tibia; ctr, cranial trochanter; di, diaphysis of humerus; dpc, deltopectoral crest; ftr, fourth trochanter; gtr, greater trochanter; h, head; histA, location of histology sample A; histB, location of histology sample B; ip, ischiadic peduncle; lc, lateral condyle of humerus; mc, medial condyle of humerus; poap, postacetabular process; pp, pubic peduncle; prap, preacetabular process; sap, supraacetabular process. Scale bars equal 10 cm; upper scale bar is for (A); lower scale bar is for (B–D).

**Figure 19:**
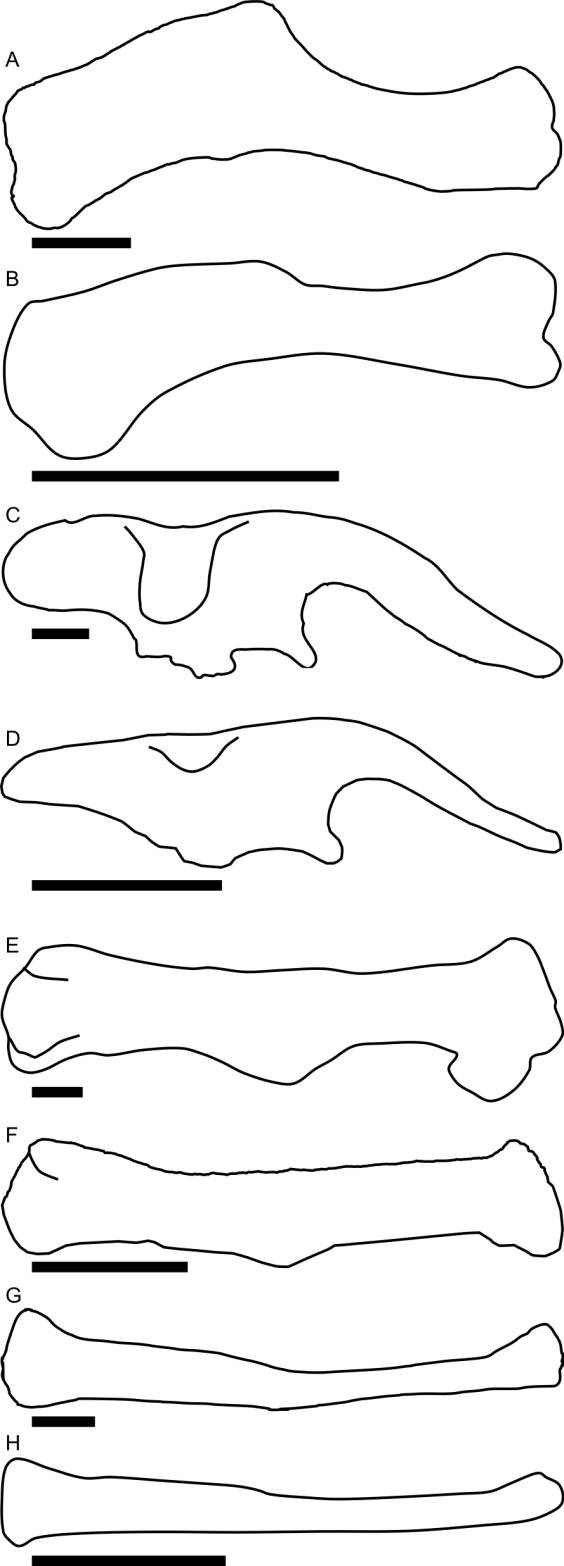
Comparisons of selected postcranial elements in adult (A, C, E, G) and juvenile (B, D, F, H) *Parasaurolophus*. (A) and (B) right humerus; (C) and (D) right ilium; (E) and (F) right femur; (G) and (H) right fibula. Juvenile elements are from RAM 14000; adult elements represent FMNH P 27393, *Parasaurolophus cyrtocristatus*, and are traced from Ostrom (1963). (A) is reversed from the original. Scale bars equal 10 cm.

The ilium is well-preserved, particularly on the right side (Figs. 2, 18B and 19D), but only the lateral surface is exposed. The caudal end is tapered, rather than tab-like and rounded as seen in *P. cyrtocristatus* (Fig. 19C) or *P. walkeri*. Little ontogenetic change in the shape of the caudal end is evident in *Hypacrosaurus stebingeri* (Horner & Currie, 1994), so this may be due to taxonomic differences or individual variation. The lateral surface of the postacetabular process is slightly concave and strongly sloped laterally, so that the ventral edge is more laterally placed than the dorsal edge. The ventral edge of the postacetabular process is slightly concave, but the dorsal margin of the blade is nearly straight, up to the preacetabular blade. This contrasts with the prominent concavity seen dorsal to the supraacetabular process in other *Parasaurolophus* (FMNH P 27393, Fig. 19C; ROM 768); development of the concavity seems to be an ontogenetic feature, more strongly pronounced in adults than juveniles (Guenther, 2009; Fig. 9). The preacetabular blade is longer and more slender than the postacetabular blade, narrowing towards the cranial-most tip. The ventral edge of the preacetabular blade is broadly sinuous. Compared to adult *Parasaurolophus*, the preacetabular process is relatively shorter (Figs. 19C and 19D). The supraacetabular process protrudes laterally and is proportionately smaller than seen in adult *Parasaurolophus* (Figs. 19C and 19D). This too is a general ontogenetic trend across hadrosaurids (Guenther, 2009). The pubic peduncle has a gentle cranial slant and is more robust and better defined than the smoothly curved ischiadic peduncle. In between the peduncles the concavity of the acetabulum is shallow and hemielliptical in profile.

Measurements for the pelvic girdle are presented in Table 8.

#### Forelimb

The forelimbs are entirely missing, with the exception of an impression of the medial surface of the right humerus (maximum length = 175 mm; Figs. 18A and 19B; Fig. S4). The deltopectoral crest extends for more than half the length of the humerus (101 mm), with a slight inward curvature at the crest’s midpoint. Compared to adult *Parasaurolophus* (e.g., *P. cyrtocristatus*, FMNH P 27393, Fig. 19A; *P. walkeri*, ROM 768), the overall form of the humerus in RAM 14000 is less sigmoidal and more slender (Fig. 19B), with a less prominent (but just as long) deltopectoral crest. This contrasts with *Hypacrosaurus stebingeri*, in which the humerus was reported to be “relatively stout” in juveniles versus larger specimens (Horner & Currie, 1994), and with negative allometry reported for the circumference of the midshaft of the humerus regressed upon the length of the humerus in *Maiasaura peeblesorum* (Dilkes, 2001; Kilbourne & Makovicky, 2010; note that the results are identical but presented differently in the two studies). Thus, RAM 14000 may suggest that *Parasaurolophus* departs from the expected allometric pattern for hadrosaurids. However, the specimen’s slender appearance could be misleading if it only preserves a portion of the bone’s profile. More specimens are needed to evaluate this hypothesis. The lateral distal condyle is more prominent than the medial condyle, but this is at least in part preservational. Measurements are presented in Table 9.

#### Hind limb

The right femur is mostly exposed although its caudomedial surface and parts of the distal condyles are partially obscured by rock (Figs. 2, 18C and 19F; Fig. S5). The head and greater trochanter are separated by a broad, v-shaped sulcus. The greater trochanter is prominent, with a flattened lateral surface and a broadly and convexly arched dorsal (proximal) margin in lateral view (Fig. 18C). The cranial trochanter extends for approximately one-fourth the length of the femur; this structure is a low and narrow process situated at the craniolateral surface of the greater trochanter. The shaft of the femur is straight, with the fourth trochanter centered at mid-shaft. The trochanter is clearly defined but relatively less prominent in terms of length and height than in adult lambeosaurines (e.g., *P. cyrtocristatus*, FMNH P 27393, Fig. 19E; *P. walkeri*, ROM 768). The medial and lateral distal condyles are not well-separated on their cranial surfaces, although a shallow depression occurs at the midline just dorsal to the condyles.

The tibia is a robust bone, approximately equal in length to the femur (Figs. 2 and 18D; Fig. S5). The cnemial crest is prominent, extending for at least a third of the tibial shaft. The crest is hooked laterally, wrapping around the cranial surface of the fibula at its proximal end. The proximal third of the crest is most robust and situated farthest from the main portion of the tibia, with the rest of the crest tapering gently towards the main shaft of the tibia. The distal end of the tibia is flattened caudally with a craniocaudal expansion relative to the mid-shaft in lateral view. A distinct ridge occurs on the caudo-lateral aspect of the distal quarter of the tibia. The proximal half of the shaft is gently concave in lateral view.

The fibula (Figs. 2, 18D and 19H; Fig. S5), as is typical for hadrosaurids, is a slender bone that tapers distally. It is strongly mediolaterally compressed, particularly at the proximal half. The proximal articular end has a fairly linear profile in comparison to larger hadrosaurids, and the caudal edge of the shaft is also quite straight. The cranial edge is gently and broadly curved, particularly at the distal half. The distal end of the fibula is gently expanded and slightly hooked cranially, articulating tightly with the calcaneum. Overall, the element is less robust and has a less prominent distal curvature than seen in larger *Parasaurolophus* (e.g., FMNH P 27393, Fig. 19G).

The calcaneum is articulated with the fibula, but only the lateral surface is exposed (Figs. 2 and 18D). It is slightly concave and has a kidney-shaped outline, with the convex end pointing distally. A small but bulbous lump of bone interposed between the distal articular surfaces of the calcaneum and astragalus may represent tarsal IV. The astragalus is insufficiently exposed to comment upon its morphology.

**Figure 20:**
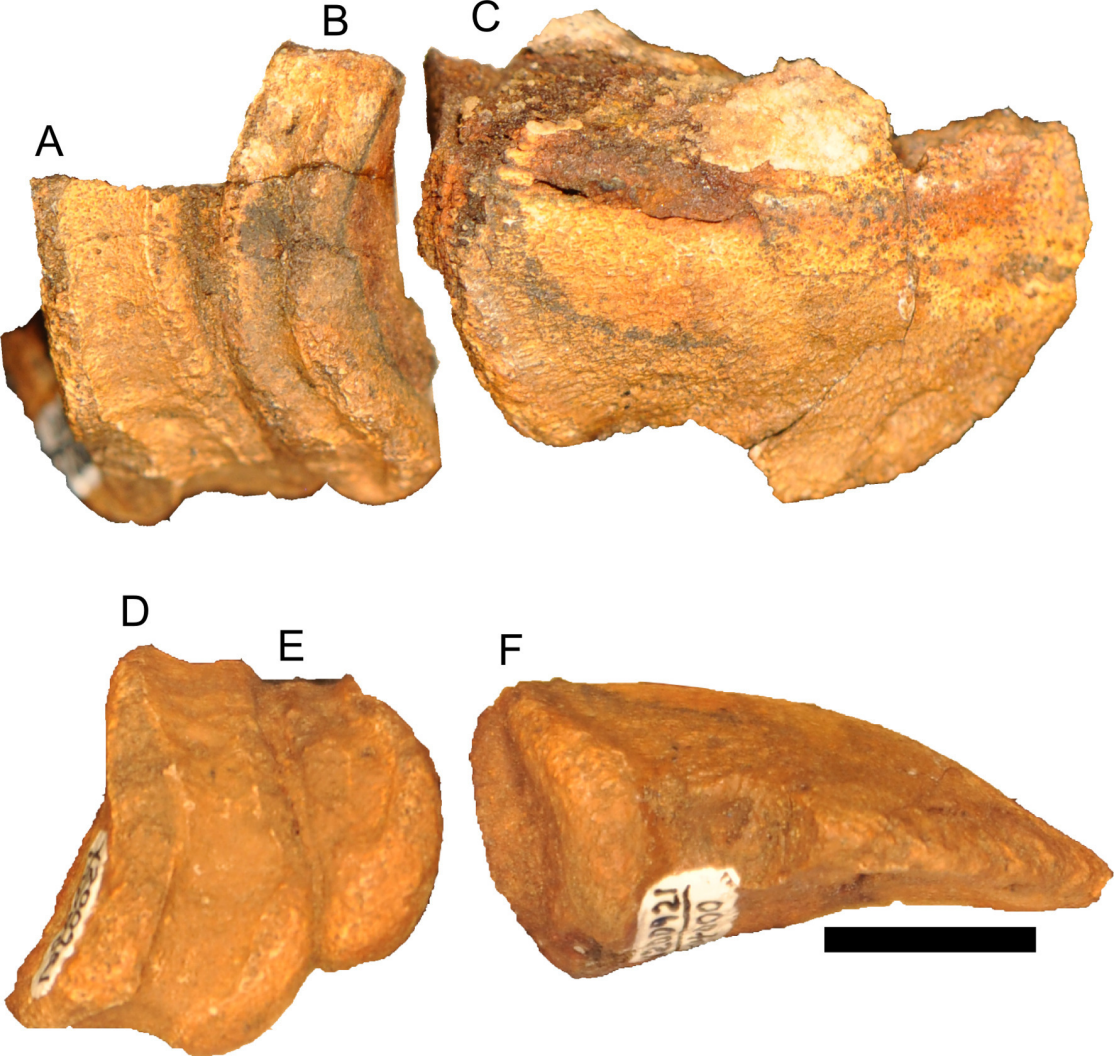
Phalanges of right pedal digit III of *Parasaurolophus* sp., RAM 14000. (A) and (D) phalanx III-2; (B) and (E) phalanx III-3; (C) and (F) phalanx III-4 (ungual); (A–C) dorsal view; (D–F) lateral view. The distal ends of the bones are to the right of the image. Scale bar equals 1 cm.

The right pes is poorly exposed, and only digits III and IV are represented (Figs. 2, 3 and 20). Digit IV conforms to the standard hadrosaurid phalangeal count of five phalanges. The first phalanx (IV-1) is longest, and following three (IV-2, IV-3, and IV-4) are considerably shorter proximo-distally. The terminal phalanx (IV-5) is expanded into a triangular ungual. Digit III has a similar pattern (Fig. 20), with the most proximal phalanx (III-1) being longest, the next two (III-2 and III-3; Figs. 20A, 20B, 20D and 20E) quite abbreviated proximo-distally, and a terminal ungual (III-4). Each phalanx after the most proximal one is broader than long. The unguals in RAM 14000 are fairly narrow (Figs. 20C and 20F), lacking the broader expansion of adults.

Measurements for the hind limb elements are presented in Table 9.

### Ossified tendons

Ossified tendons or their impressions are visible at only two portions of the skeleton. The first set occurs lateral and ventral to the neural spine of the ?fourth dorsal vertebra (Fig. 17A). Two tendons are preserved here, both of which roughly parallel the long axis of the vertebral column. They are approximately 0.8 mm in maximum diameter, and the longest preserved segment is 21 mm long (including an impression of the tendon in the measurement. Both tendons occur on the lateral surface of the caudal quarter of the neural spine and just dorsal to the transverse process, and trend very slightly ventrally. The longest preserved one extends at least to the cranial quarter of the next spinous process. Based on the position and orientation of these tendons, we hypothesize that they represent portions of M. iliocostalis or potentially M. longissimus dorsi (Organ, 2006).

A second set of ossified tendons, represented only by impressions, occurs ventral to the margins of the impressions of the neural spines of vertebrae and dorsal to the fragments of the transverse processes on the left side (Fig. 17B). The tendons themselves were destroyed by weathering prior to discovery. The exact position of the tendons in the vertebral column cannot be determined, but the tendons lie just dorsal to the cranial end of the ilium and thus at the very caudal end of the dorsal vertebrae or cranial end of the sacral vertebrae. At least seven parallel tendons occur here, with a maximum diameter of each impression 2.0–2.5 mm and the longest impression with a preserved length of at least 55 mm (and probably longer). The tendons were lateral to the neural spines, moving dorsally towards the caudal direction (i.e., caudodorsally inclined). Based on the position and orientation of these impressions, we hypothesize that they represent tendons of M. tendinoarticularis within M. transversospinalis (Organ, 2006).

### Integument

Soft (non-bone) tissue impressions are preserved around the left foot and rostral end of the skull. Despite careful mechanical preparation with an aim to identify other areas of soft tissue preservation, no additional, unambiguous impressions were identified.

#### Upper rhamphotheca

A series of parallel, dorsoventrally oriented grooves rostral and ventral to the oral margin of the premaxilla (Figs. 8A, 8B and 8E) are interpreted as impressions of the internal surface of the upper rhamphotheca, in light of similarly interpreted anatomy in other hadrosaurids (Morris, 1970). From the inferred midline, a total of eight grooves are preserved (Fig. 8E); they may have extended farther laterally. Each groove is 2.5–3.5 mm wide. In rostral view, the ventral edge of the series dips ventrally from medial to lateral. This suggests a broad, inverted “V” profile for the complete series when including both left and right sides of the skull (Fig. 6C). The greatest mediolateral width of the series is 38 mm. The greatest preserved dorsoventral depth of the preserved flutes is 16 mm, but their proximal and lateral portions were inadvertently prepared away. Thus, the distance between the oral margin of the premaxilla and the distal extremity of the rhamphotheca averaged around 25 mm.

These impressions indicate that the soft-tissue profile of the oral margin extended significantly beyond the bone (Fig. 7A). This is consistent with previous reports of an internally fluted beak that extended well beyond the premaxilla in *Edmontosaurus annectens* (Versluys, 1923; Morris, 1970). The margins of the premaxilla and impressions are closely parallel in *E. annectens*, indicating that bone shape is a fairly accurate proxy for soft tissue shape. We hypothesize a similar pattern for *Parasaurolophus* based on RAM 14000.

A specimen of *Corythosaurus casuarius* (CMN 8676) originally preserved a portion of the impressions of the rhamphotheca (Sternberg, 1935). Initially interpreted as the lower rhamphotheca (Ostrom, 1961), we agree with later interpretations of the structure as the upper rhamphotheca (Morris, 1970). Only a fragment of this impression is now available. Based on the original photographs (Sternberg, 1935), the internal surface of the upper rhamphotheca was grooved as in RAM 14000. However, we cannot determine with confidence the shape of the margin of the rhamphotheca in CMN 8676 for comparison. Although such features are not evident in RAM 14000, projections from the oral margin of the premaxilla in many hadrosaurids may correlate with the grooves on the rhamphotheca. Additional work is needed to verify this.

#### Skin impressions

**Figure 21:**
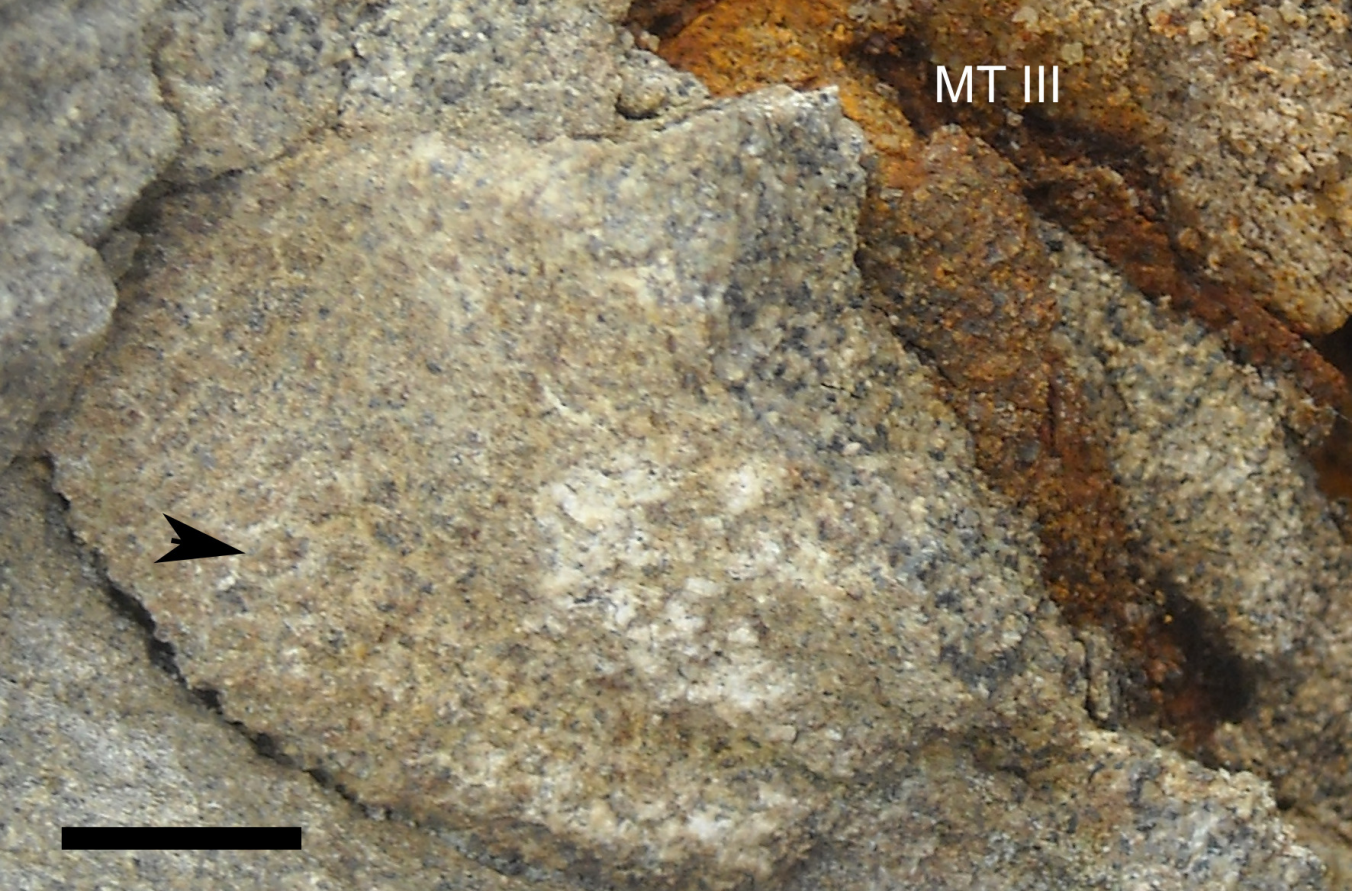
Skin impressions of *Parasaurolophus* sp., RAM 14000, from plantar surface of right pedal digit III. The proximal end of the digit is to the lower right end of the image, and the distal end is towards the top middle edge of the image. The arrow indicates one individual tubercle. Abbreviations: MT III, caudal surface of metatarsal III. Scale bar equals 1 cm.

Two small (<5 cm maximum dimension) patches of skin impressions are associated with the region caudal to the right metatarsal III and phalanx III-1 (Figs. 3 and 21). This impression is gently folded upon itself, and covered by non-imbricating, roughly circular tubercles that average ∼2 mm in maximum diameter (Fig. 3; pebble-type basement scales of Bell, 2012). The impression was exposed to weathering prior to discovery, and thus surface detail is muted. The overall appearance, including the folding, is reminiscent of the equivalent region in *Corythosaurus casuarius* (AMNH 5240; Brown, 1916). The only major difference in RAM 14000 concerns the smaller tubercles relative to AMNH 5240, which are undoubtedly related to the animal’s small body size.

### Bone histology

We describe the histology of the tibia based on two samples from the caudolateral quadrant of the proximal shaft, close to the mid-diaphysis (see Fig. 18D for positions). We follow the terminology of Francillon-Vieillot et al. (1990), with additional terminology related to the orientation and arrangement of osteocytes following Werning (2012).

**Figure 22:**
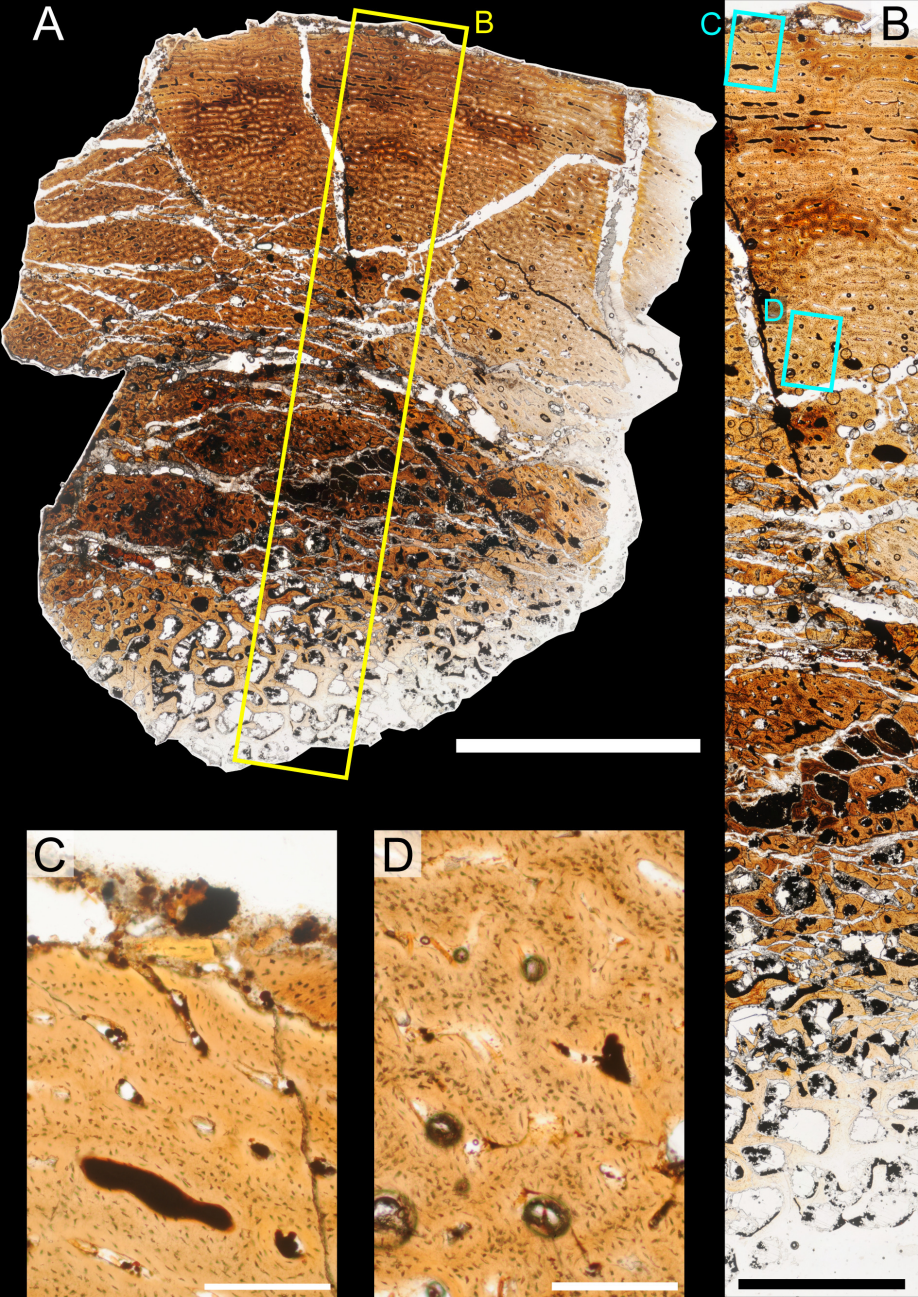
Bone microstructure of juvenile *Parasaurolophus* tibia in regular transmitted light (RAM 14000, histological sample B, near mid-diaphysis; see Fig. 18D for position of sample). (A) entire sample in cross-section (transverse plane), with inset box showing the position of B; (B) radial “transect” across A, with inset box showing positions of enlargements C and D; (C) and (D) enlargements of B showing primary osteons and osteocytic arrangement. The primary cortex is virtually unremodeled and shows no lines of arrested growth. Most longitudinal primary osteons anastomose circumferentially with two to five other canals, especially in the mid-cortex. The woven component of the bone is less prominent in the outer cortex (C) compared to the midcortex and inner cortex (D). The periosteum lies to the top of each image. Scale bar equals 5 mm for (A), 2 mm for (B), and 250 µm for (C) and (D).

**Figure 23:**
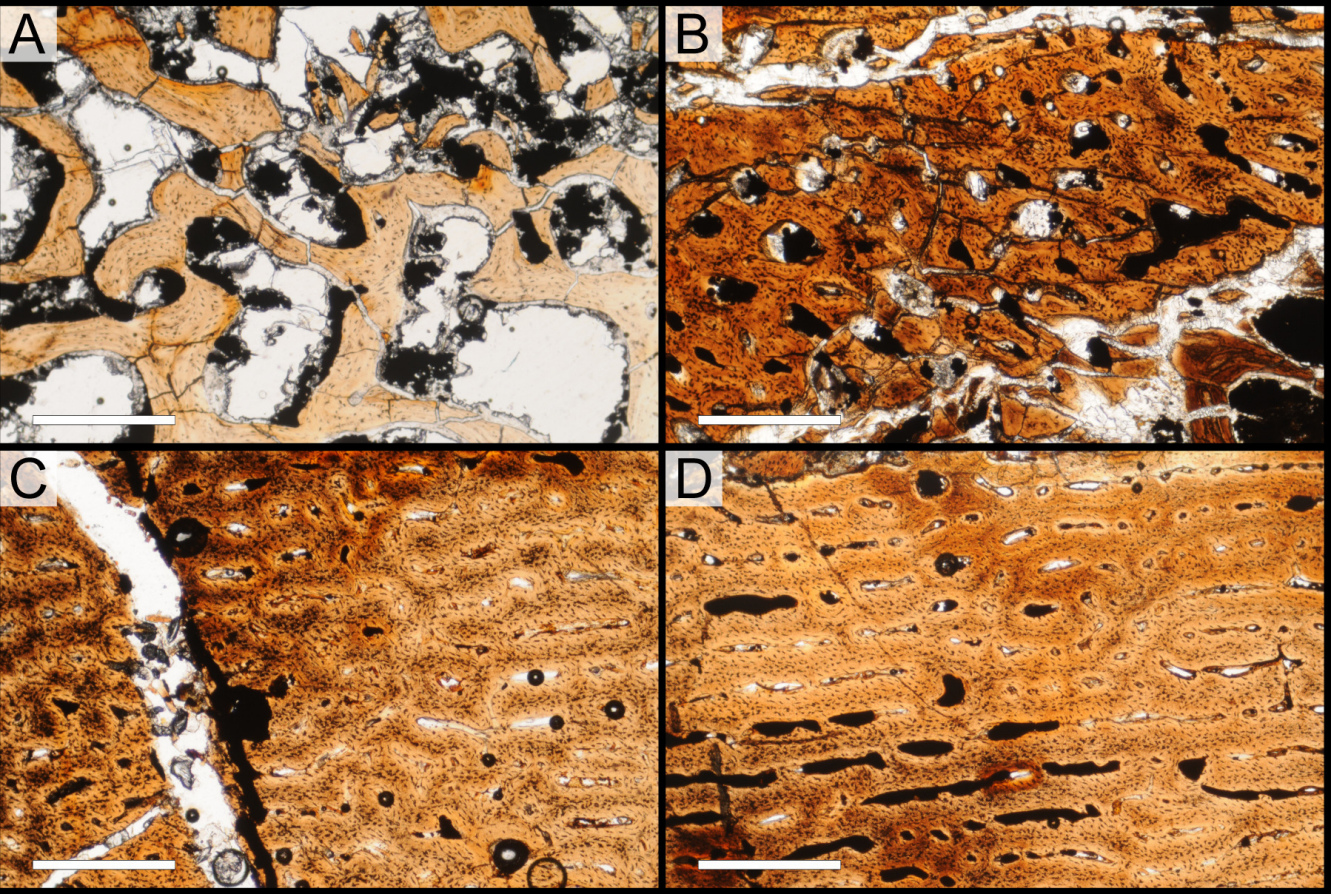
Bone microstructure of juvenile *Parasaurolophus* tibia in regular transmitted light (RAM 14000, histological sample B, cross-section near mid-diaphysis; see Fig. 18D for position of sample). (A) inner cancellous region; (B) outer cancellous region; (C) inner/mid-cortex; (D) outer cortex close to the periosteum. In (A), the cores of trabeculae are comprised of unremodeled primary woven bone tissue and the edges lined by pseudolamellae of parallel-fibered bone and true lamellar bone. In (B), incipient cancellous bone, forming by expansion of canals. Most spaces are unlined. In (C), woven bone forms much of the laminae and parallel-fibered bone lines the primary osteons rather than lamellar bone. In (D), longitudinal simple primary canals and primary osteons begin to anastomose laterally. Osteocyte density is noticeably higher in (B), (C), and (D) compared to (A), though they are randomly oriented through the entire section. The periosteum lies to the top of each image. All scale bars equal 500 µm.

Section B (Figs. 22 and 23) lies closer to the mid-diaphysis. The section is ∼15–16 mm thick ( = radially “deep”) including the cortical and cancellous bone. The cancellous region comprises the inner 3–7 mm of the sample. A good deal of fracturing is visible throughout the section. In the cancellous region, the cracks are infilled by crystals and/or a dark, amorphous matrix, but in the cortex, many of the cracks lack infilling. It is possible that these cortical cracks formed during extraction from the ground or extraction of the sample for histological preparation. Crystals and the black matrix also infill the interstices between trabecular and the canals of the cortex. The bone is strongly birefringent, but shows a negative optical sign when viewed under elliptically polarized light (e.g., Fig. 25). This suggests that the collagen has been secondarily replaced by apatite crystals (Lee & O’Connor, 2013).

**Figure 24:**
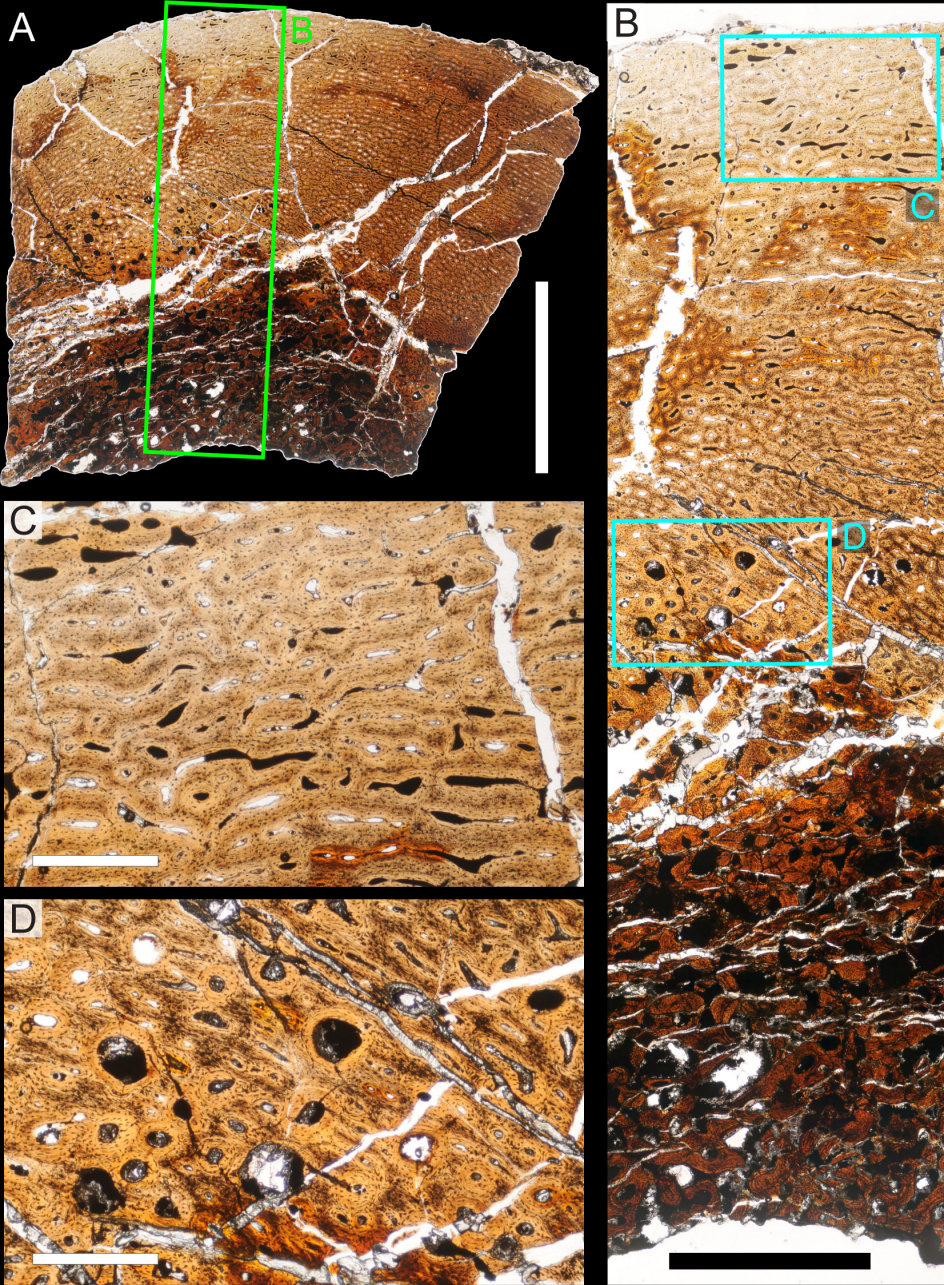
Bone microstructure of juvenile *Parasaurolophus* tibia in regular transmitted light (RAM 14000, histological sample A, through proximal portion of diaphysis; see Fig. 18D for position of sample). (A) Entire sample in cross-section (transverse plane); (B) radial “transect” across A; (C) and (D), enlargements of B showing primary osteons and osteocytic arrangement. Proximal in the diaphysis, the cortex is better organized (C) compared to the mid-diaphysis (Fig. 22) and there is much more secondary remodeling in the inner cortex (D). The periosteum lies to the top of each image. Scale bar equals 5 mm for A, 2 mm for B, and 250 µm for C and D.

**Figure 25:**
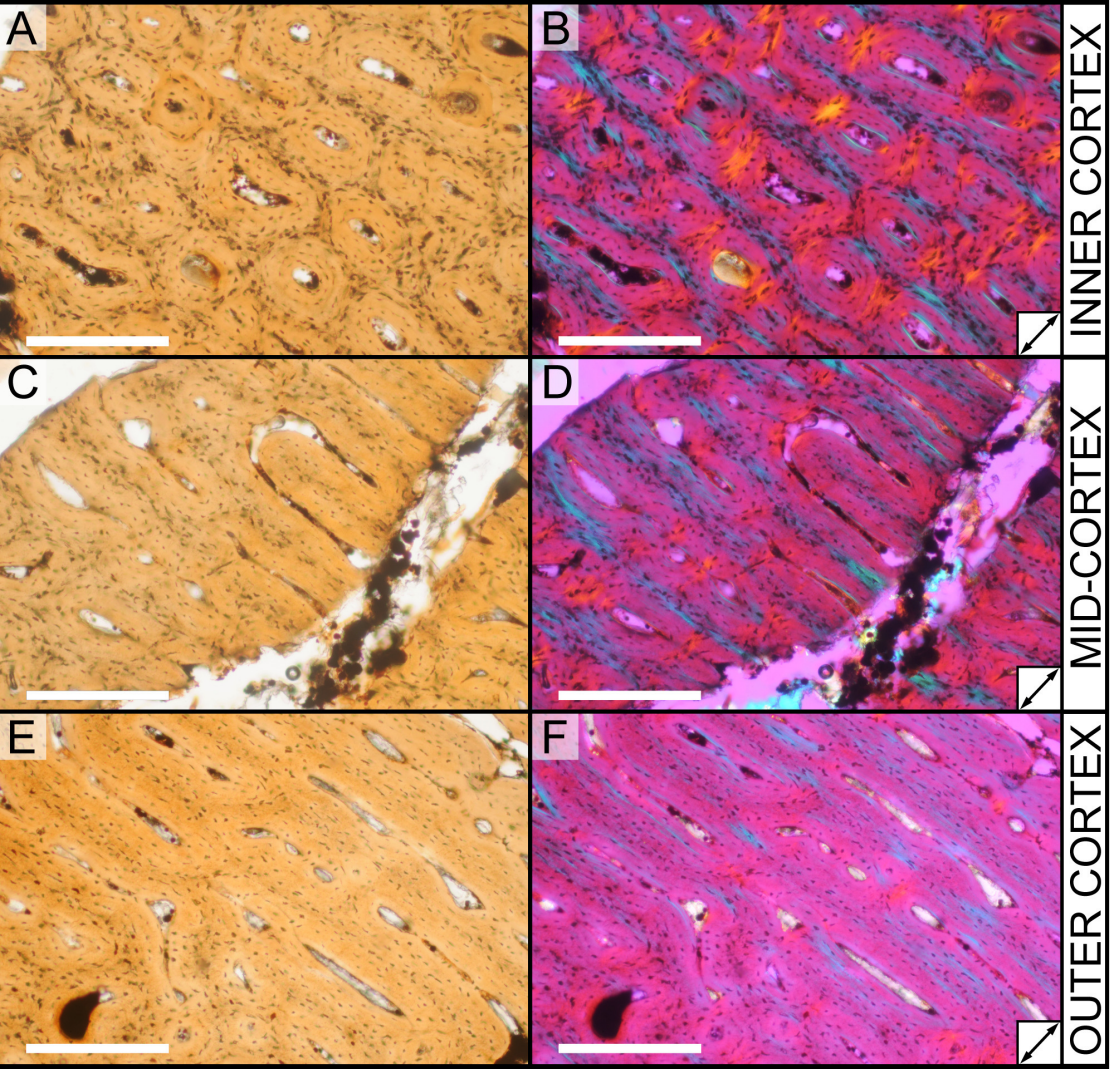
Histological variation in the cortex of juvenile *Parasaurolophus* tibia in regular transmitted light (A, C, E) and elliptically polarized (B, D, F) light (RAM 14000, sample A). The elliptically polarized light is under full λ retarder plate, and the sample is a cross-section through the proximal portion of the diaphysis. Moving periosteally through the cortex, the bone tissue comprising the laminae becomes progressively more organized. In the inner cortex (A, B), osteocytes are densely packed in the interstices between vascular canals, and their lacunae are oriented randomly with respect to the long axis of the bone and to each other. In this region, the laminae are mainly comprised of woven bone, with lamellar bone surrounding each vascular canal. In the mid-cortex (C, D), woven bone comprises a smaller portion of the laminae, and parallel-fibered bone lies between the woven and lamellar components. In the outer cortex (E, F), at most only a thin band of woven bone lies in the cores of the laminae, and much of the interstices are comprised of parallel-fibered bone. The periosteum lies to the upper right of each image. Arrows in (B), (D), and (F) indicate the orientation of the slow axis of the λ plate. All scale bars equal 250 µm.

The internal-most portion of the cancellous region (Figs. 22A and 23A) shows thick trabeculae that delineate large (up to 1 mm) amorphous intertrabecular chambers. The cores of the largest trabeculae are comprised of woven or parallel-fibered primary bone and the edges are lined with several lamellae (true lamellar bone) or pseudolamellae (loose layers of parallel-fibered bone). The lamellae do not appear to cut across the cores; rather, they appear to have been deposited appositionally to them. The osteocyte density is much higher in the trabecular cores than in the lamellar/pseudolamellar bone lining the trabecular margins; in fact, they appear as densely packed as they are in the primary woven bone that forms the internal-most cortex. These osteocytes are aligned along the long axis of each trabecula and change orientation as trabecular orientation changes. This strongly suggests that at least some of these trabeculae formed *de novo* rather than by resorption of primary cortical tissues; if this is correct, these are remnants of embryonic or perinatal bone tissue.

The appearance of the trabeculae in this region is extremely similar to that described for embryonic ornithopods, including those of *Maiasaura* (Horner, de Ricqlès & Padian, 2000; Horner, Padian & de Ricqlès, 2001), *Hypacrosaurus* (Horner & Currie, 1994; Horner, Padian & de Ricqlès, 2001), *Dryosaurus* (Horner et al., 2009) and *Tenontosaurus* (Horner et al., 2009; Werning, 2012). Given the tibial diameter of ∼40 mm in RAM 14000, and that this region comprises no more than 3.5 mm of the preserved section (i.e., there is 11.5–12.5 mm of cortical bone external to it), the neonatal tibial diameter could not have exceeded 15–17 mm (47–53 mm circumference). This is very close in size to the embryonic femora of *Hypacrosaurus* (32.5–40 mm circumference; Horner & Currie, 1994).

Other, incipient cancellous bone is visible just external to the preserved embryonic tissues (Fig. 23B). Contrary to the cancellous bone described above, this tissue is not as porous and clearly formed by the resorption of primary cortical tissues. In this region, the cores of the incipient trabeculae comprise primary woven bone, but the orientations of primary osteons and osteocytes do not correspond with trabecular orientation. Erosion rooms ranging in size from .1–2 mm cut across primary bone tissue, and many are unlined. Where lamellar bone lines the erosion rooms, it cuts across the primary tissues forming the cores of the incipient trabeculae.

The cortex near the mid-diaphysis is comprised mainly of well-vascularized, woven primary bone tissue. In the inner cortex (Fig. 23C), the bone is exclusively woven and the canals are a mixture of primary osteons and simple primary canals. The canals of this region are mainly longitudinal, but short radial, circumferential, and oblique canals are also common. The canals in this region are not as organized as the mid- or outer cortex and generally anastomose with several (two to four) other canals. Osteocyte density in this region is extremely high, and osteocytes show no preferred orientation relative to the long axis of the bone nor a preferred arrangement relative to each other. Osteocytes encircle some primary osteons, but equally often, they are oriented oblique to the canals in the tissue that surround them. In the regions closest to the canals, the bone is less cellular compared to the cores of the laminae/interstices between them.

In the mid- and outer cortex (Fig. 23D), the bone is similar in its components but shows more organization in its vascular canals and in its osteocytes. Vascular canals are again a mix of primary osteons and simple primary canals, which are generally wider in diameter compared to those of the inner cortex. As noted by Starck & Chinsamy (2002), this reflects the ontogeny of the canals themselves; the inner canals are older and have had more time to deposit bone since the initial bone scaffolding was deposited. Canals in this region show strong circumferential signal; in the mid-cortex, circumferential canals dominate, and in the outer cortex, the longitudinal canals are arranged circumferentially in rows. Osteocyte density is lower in the outer cortex compared to the inner cortex. Additionally, the disorganization is confined to a slightly narrower region at the center of each lamina. In the outermost cortex, the canals are often encircled by a thin band of fairly acellular bone (Fig. 23D). In some cases, it appears that a region of parallel-fibered bone, less dense in osteocytes, separates the acellular lining bone from the “core” of woven bone at the center of each lamina. Despite histological indications that bone deposition rate was slightly lower in the outer cortex compared to the inner cortex, no annuli or lines of arrested growth (LAGs) are visible in this section.

**Figure 26:**
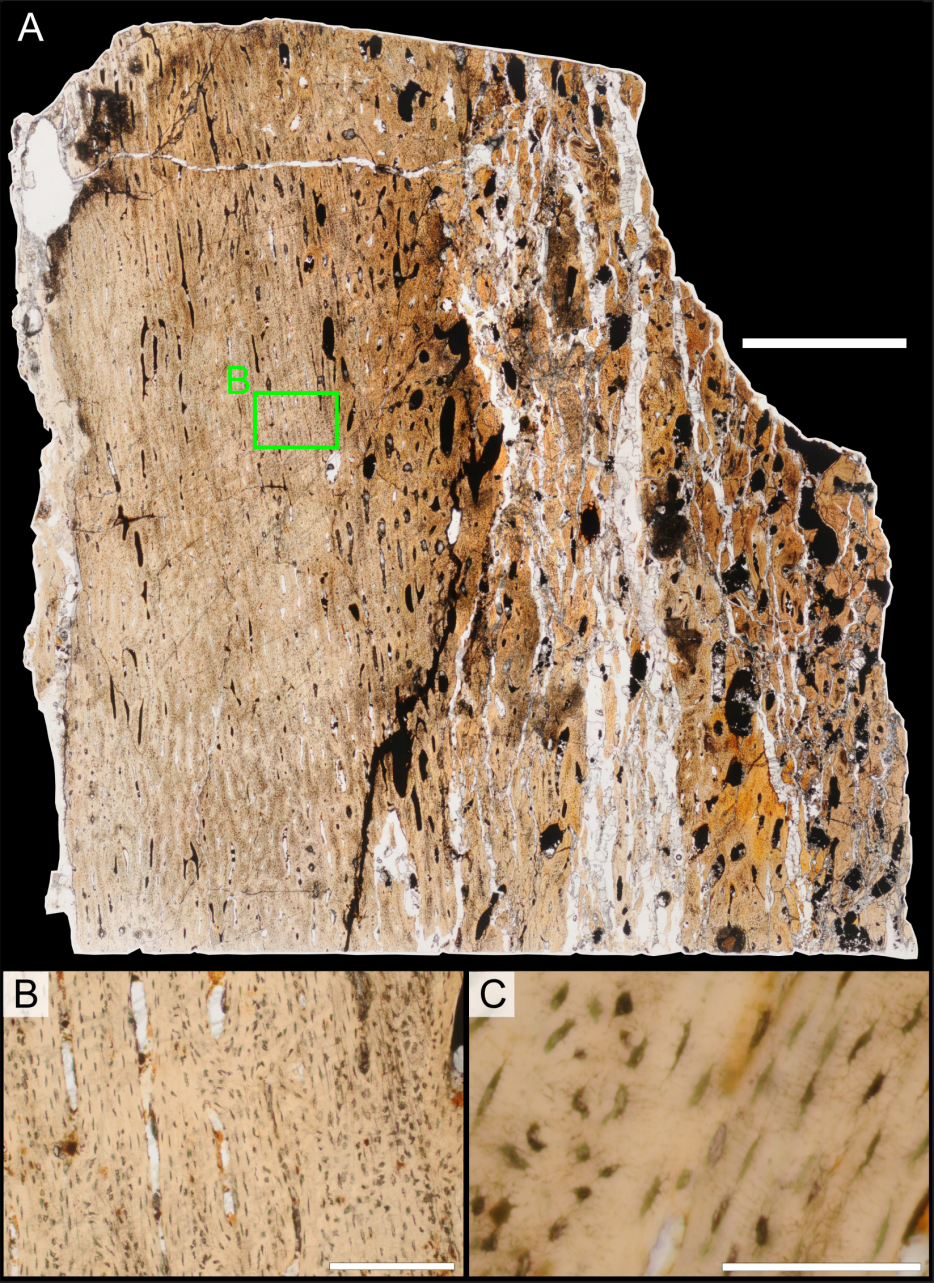
Bone microstructure of juvenile *Parasaurolophus* tibia in regular transmitted light (RAM 14000, sample A; proximal portion of diaphysis). (A) Entire sample in longitudinal section; (B) enlargement of (A) showing primary osteons and osteocytic arrangement in the cortex; (C) osteocytes. The amount of woven bone in the laminae between primary osteons varies through the cortex. In (B), osteocytes in regions of woven bone are randomly oriented and densely packed (e.g., to right of image). Closer to canals, osteocytes are fewer in number and much more organized (center of image). In (C), the more disorganized woven bone is visible on the left side of the image and the more organized parallel-fibered bone is to the right. The periosteum lies to the left in all images. Scale bar equals 2 mm for (A), 250 µm for (B), and 100 µm for (C).

Section A (Figs. 24–26) is taken from a more proximal portion of the diaphysis. The section is ∼11–12 mm thick (deep), including the cortical and cancellous bone. The cancellous region comprises only the inner ∼2.5 mm of the sample. The section sampled in longitudinal section runs proximally along the shaft for 12 mm from the site of the cross-sectional sample. In cross-section (Fig. 24), section A resembles section B in its vascular patterning. It differs in the degree of organization of the primary cortical tissues (Figs. 24C and 25), in the secondary remodeling of the inner cortex (Fig. 24D), and in its thicker and more closely spaced trabeculae.

As in Section B, the trabeculae show no secondary osteons in their cores. The edges of all trabeculae are lined with distinct lamellae, often five or more in number. Some of these trabeculae clearly formed by resorption and secondary deposition; the lamellae on one side of a trabecula often cut through the lamellae on the other side. Although a few larger erosion rooms are present, most are between .1 and .5 mm in diameter.

The inner cortex was clearly experiencing active secondary remodeling at the time of the animal’s death. Distinct erosion rooms of varying age (based on number of lamellae) are visible throughout the innermost cortex; these may be up to .15 mm wide. Several generations of secondary osteons are also visible (Fig. 24D). Although some primary tissue is clearly visible between secondary osteons, they cut across each other in places.

The primary cortical tissues increase in organization moving periosteally through the section (Fig. 25). As in section B, the inner cortex of section A (Figs. 25A and 25B) shows highly disorganized bone tissue. The interstices between vascular canals (nearly all are randomly arranged longitudinal primary osteons) form laminae mainly comprised of woven bone that shows a high density of randomly oriented osteocytes. Parallel-fibered bone or, more often, lamellar bone encircles the canals themselves. This is also cellular, but not to the extent of that in the interstices. The boundary between the woven bone and lamellar bone is distinct. This bone is clearly “fibro-lamellar tissue” as defined by Francillon-Vieillot et al. (1990).

In the mid-cortex (Figs. 25C and 25D), the bone is more laminar, because the primary osteons anastomose circumferentially with adjacent canals. Here, the woven “cores” of the interstitial bone have fewer osteocytes, but the ones that are present are equally disorganized. The bone encircling the primary osteons is exclusively lamellar and less cellular than in the inner cortex. Very thin bands of parallel-fibered bone lie between the woven component and the lamellar component surrounding the canals. Thus, the woven component grades into the lamellar component and the boundary between interstitial and circumvascular bone tissue is not as abrupt. In the outer cortex (Figs. 25E and 25F), woven bone is restricted to the center of the interstitial “cores” and much more parallel-fibered bone separates the woven component from the lamellar component. In this region, far fewer osteocytes occur in either the interstices or the lamellar bone of the primary osteons. The same pattern is also evident in longitudinal section (Fig. 26).

Stein & Prondvai (2013) describe a similar condition in the long bones of the sauropods *Alamosaurus*, *Apatosaurus*, and *Camarasaurus*. In these taxa, woven bone is restricted to a thin splint at the center of bony laminae, and highly organized parallel-fibered and lamellar bone fills the space between the woven splint and the vascular canals. This reflects the process of bone deposition; a scaffold of woven bone is deposited rapidly around vascular canals so that the diameter of the bone can be rapidly expanded. Subsequently, more organized tissues are deposited onto this scaffold (Stein & Prondvai, 2013).

### Body size and completeness

Aside from RAM 14000, the most complete and smallest associated juvenile lambeosaurine skeleton is AMNH 5340, referable to *Lambeosaurus* sp. (Evans, 2010). This individual had a total length of 4.31 m (Lull & Wright, 1942), and comparable-sized postcranial elements are 1.74 to 1.89 times the length of those in RAM 14000 (Table 10; ischium length is excluded as an outlier). Scaling from AMNH 5340, we estimate total body length in RAM 14000 conservatively at 2.28 to 2.48 m, considerably smaller than the 9.45 m total body length estimated for the holotype of *Parasaurolophus walkeri*, ROM 768 (Lull & Wright, 1942).

Associated cranial bones from the Kirtland Formation of New Mexico, SMP VP-1090, were tentatively identified as a juvenile *Parasaurolophus* sp. (Sullivan & Bennett, 2000). The quadrate in this specimen is 185 mm long, 67 percent larger than the quadrate in RAM 14000 (111 mm long). A braincase assigned to juvenile *Parasaurolophus* sp. from the Dinosaur Provincial Park region of Alberta, CMN 8502, has a frontal width of 38 mm, 20 percent larger than the equivalent dimension in RAM 14000 (31.7 mm). By contrast, the skull length (horizontal from rostrum to paroccipital process) in the *P. walkeri* holotype (ROM 768) is 745 mm versus 246 mm in RAM 14000, or 303 percent larger. Thus, RAM 14000 represents the smallest confidently identifiable specimen of *Parasaurolophus* known to date.

In terms of skeletal representation, RAM 14000 is the most complete single individual of *Parasaurolophus* described to date (Table S1). Approximately 46 percent of skeletal elements are preserved here, contrasting with 43 percent in the holotype of *P. walkeri* (ROM 768) and 35 percent in the holotype of *P. cyrtocristatus* (FMNH P 27393).

### Crest acoustics

Following the methods of Weishampel (1981a), we estimated the resonant frequencies of the main passageway of the nasal cavity for RAM 14000. Because the lateral diverticulum is poorly separated from the rest of the nasal passages and is not close-ended in RAM 14000, we did not calculate its corresponding resonant frequency. Necessary parameters to calculate *f* (frequency, in Hz) included *n* (resonance mode, set between 1 and 5), *v* (velocity of sound at sea level, 340 m/s), and *L* (length of tube, set at 0.195 m for RAM 14000 as measured from CT scan data). These parameters were entered into the following equation:

\begin{eqnarray*} \displaystyle f=n(v/2 L)&&\displaystyle \end{eqnarray*}

Results are summarized in Table 11. The estimated resonant nasal frequencies of RAM 14000 are approximately 11 to 18 times higher than those for *P. cyrtocristatus* and *P. walkeri*, respectively, as expected given the difference in cavity lengths.

**Table 11:** Estimated resonant frequencies of the crest in *Parasaurolophus* skulls. Data for ROM 768 and FMNH P 27393 are from Weishampel (1981a).

			Frequency (Hz)				
Taxon	Specimen	Tube length (m)	Mode 1	Mode 2	Mode 3	Mode 4	Mode 5
*P. walkeri*	ROM 768	3.46	48	96	144	192	240
*P. cyrtocristatus*	FMNH P 27393	2.21	75	150	225	300	375
*P.* sp.	RAM 14000	0.195	872	1,744	2,616	3,488	4,360

### Mandibular mechanics

Most discussions of hadrosaurid jaw mechanics have focused, directly or indirectly, on the dental occlusal surfaces and movements associated with that complex (e.g., Ostrom, 1961; Weishampel, 1983; Erickson et al., 2012), but no detailed consideration has been given to the potential mechanical consequences of the premaxillary “beak”. Adding a keratinous rhamphotheca increases the minimum gape at which contact between upper and lower jaws is made with a food item, with corresponding effects upon bite force.

**Figure 27:**
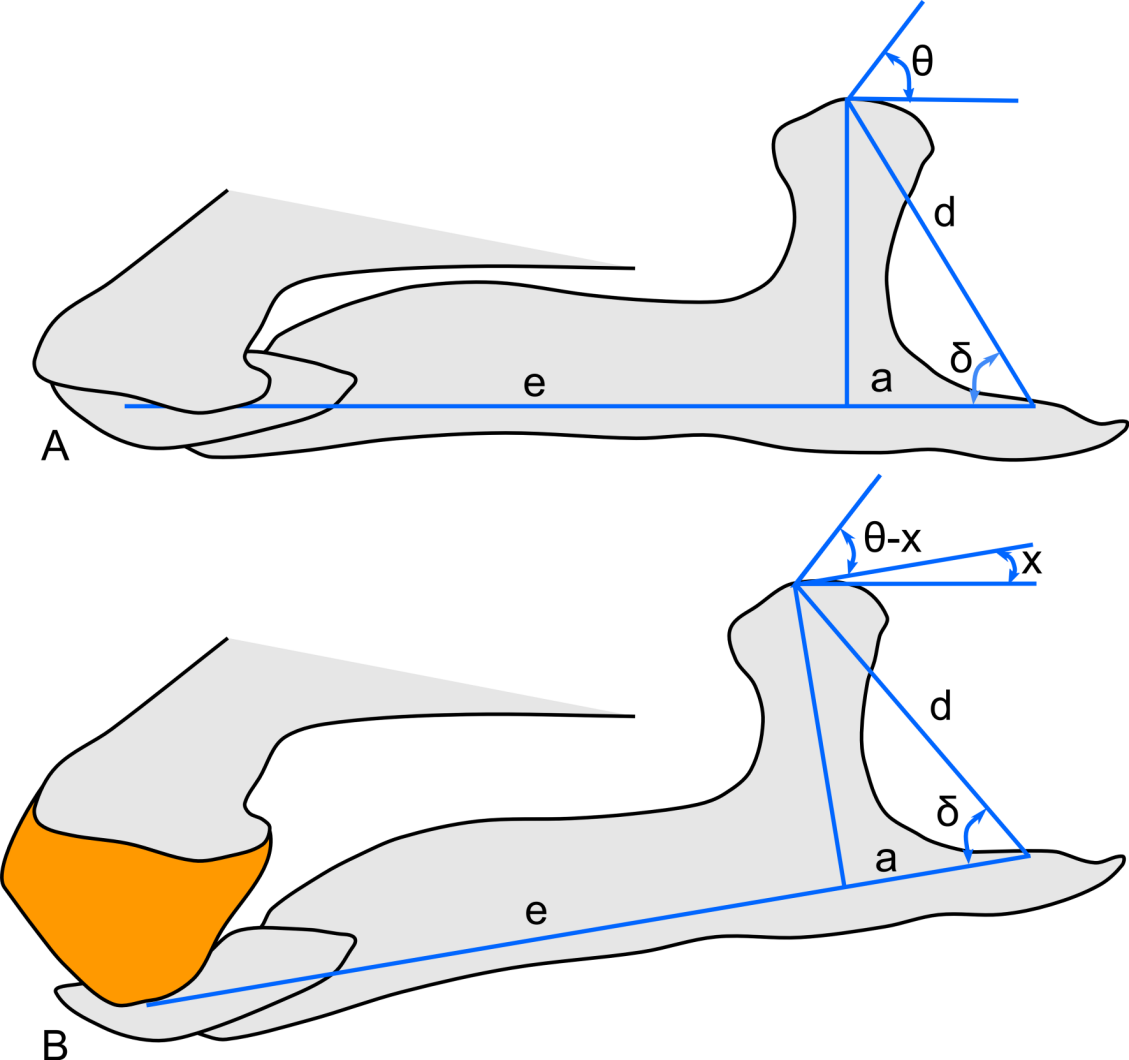
Schematic of the hadrosaur jaw system, showing the effects of a rhamphotheca on bite mechanics. In (A), the jaws are shown without the rhamphotheca, in the approximate position where the upper and lower bony beak surfaces would first make contact. In (B), the jaws are shown with the upper rhamphotheca (orange), in the approximate position where the upper and lower beak surfaces would first make contact. Note that this is at a wider gape than in (A). Abbreviations: *a*, distance from the center of the glenoid to the center of the coronoid process; *d*, distance from the glenoid to the top of the coronoid process; *e*, distance from the center of the coronoid process to the bite point; θ, the angle between the line of the jaw and the applied force on the coronoid process (measured from the center of the supratemporal fenestra); δ, the angle of the diagonal between the top of the coronoid and the glenoid, relative to the line of the jaw. *x* is the change in gape angle produced by the rhamphotheca. The equation to calculate bite force is given in the text.

As in previous studies (e.g., Ostrom, 1964; Bell, Snively & Shychoski, 2009), the ornithischian lower jaw can most simply be approximated as a third class lever, with the applied muscle force located between the fulcrum (glenoid) and the resistance (usually the dentition, but in this case the predentary). Here, the force lever arm is the distance between the coronoid process and glenoid, whereas the resistance lever arm is the distance from bite point to glenoid (following Ostrom, 1964). In order to calculate usable force at a given point on the mandible, five parameters are needed (see Ostrom, 1964, for a full explanation): *e*, distance from the center of the coronoid process to the bite point; *a*, distance from the center of the glenoid to the center of the coronoid process; *d*, distance from the glenoid to the top of the coronoid process; θ, the angle between the line of the jaw and the applied force on the coronoid process (measured from the center of the supratemporal fenestra); and δ, the angle of the diagonal between the top of the coronoid and the glenoid, relative to the line of the jaw (Fig. 27).

All of the parameters are then entered into the equation:

\begin{eqnarray*} \displaystyle S(e+a)=F\sin (\theta +\delta )d&&\displaystyle \end{eqnarray*}

where *S* is the percentage of usable force at a given point relative to the input force *F*. All variables are the same in all conditions, except for θ. When accounting for increased gape due to the rhamphotheca, θ is accordingly reduced (Fig. 27B). Thus, at a gape of 10°, θ–10° would be used as the appropriate value. Here, we make the simplification that *F* is constant across the relatively small differences in gape considered here.

Based on measurements from the original specimen, the rhamphotheca in RAM 14000 decreased the angle of gape at which upper and lower beaks contacted by approximately 7°. Thus, θ = 44° without a rhamphotheca and θ = 37° when the rhamphotheca is included. For other parameters, *e* = 150 mm (measured to rostral-most extent of predentary), *a* = 48 mm, *d* = 80 mm, and δ = 42°. Because absolute values are not a concern, *F* was set at 100%.

Using the above numbers and equation, the percentage of usable force at the predentary is 40.3% without the rhamphotheca and 39.6% with the rhamphotheca. Thus, the rhamphotheca introduced a 0.7% decrease in bite force relative to the condition without.

## Discussion

### RAM 14000 is *Parasaurolophus*

Although the visually striking and taxonomically diagnostic crests of lambeosaurine hadrosaurids do not reach their ultimate morphology until adulthood, many genus- and species-level autapomorphies appear earlier in ontogeny (Evans, Forster & Reisz, 2005; Evans, Reisz & Dupuis, 2007; Evans, 2010; Brink et al., 2011). Based on a combination of anatomical features in RAM 14000, as well as stratigraphic and geographic evidence, we identify the specimen as a juvenile *Parasaurolophus*. However, we do not assign it to a particular species.

Although both hadrosaurids and basal neornithischians (“hypsilophodontids”) occur in the Kaiparowits Formation (Gates et al., 2013), RAM 14000 is clearly identifiable as a hadrosaurid. It possesses numerous synapomorphies not found in “hypsilophodontids,” including three or more replacement teeth in each tooth family as well as absence of a surangular foramen or free palpebral (Horner, Weishampel & Forster, 2004).

Furthermore, a series of synapomorphies clearly place RAM 14000 within Lambeosaurinae. These include a domed frontal in subadults, development of an enlarged crest formed from the premaxillae and nasals as well as a nasal vestibule completely enclosed by the premaxillae, a triangular rostrolateral corner of the premaxilla, and many others (Horner, Weishampel & Forster, 2004; Prieto-Márquez, 2010).

**Figure 28:**
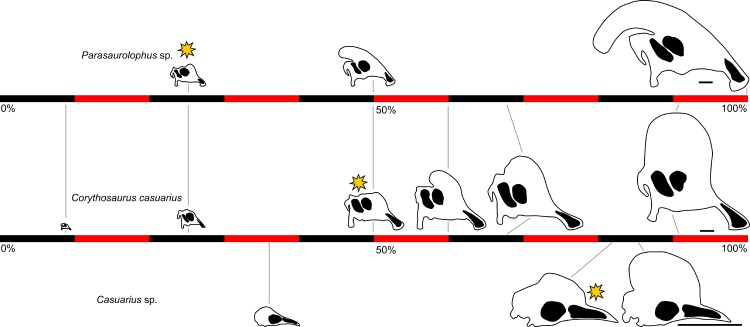
Cranial growth series for *Parasaurolophus*, *Corythosaurus*, and *Casuarius*, showing changes in bony ornamentation relative to maximum reported skull length for each taxon. The black and red scale indicates percentage of maximum reported skull length in increments of 10 percent. The yellow sunburst indicates the approximate skull size at which ornamentation initially appears. Note that *Parasaurolophus* develops its crest at a very small skull size relative to *Corythosaurus*, and both hadrosaurids initiate the development of cranial ornamentation at a smaller relative skull size than in *Casuarius*. Skulls for *Parasaurolophus* sp. are based on RAM 14000, a hypothetical subadult (Fig. 11B), and the holotype for *Parasaurolophus cyrtocristatus* (FMNH P 27393, with missing elements patterned after ROM 768). The growth series for *Corythosaurus* is a composite, with the two smallest skulls (at left) patterned after *Hypacrosaurus stebingeri*. Because the two taxa are so closely related, and because they show broadly similar patterns of cranial growth where individuals of overlapping size are known (Evans, 2010; Brink et al., 2011), we consider this a reasonable assumption. The smallest skull (at left) is based on RTMP 89.79.52, 87.79.206, 87.79.241, 87.77.92, and 87.79.333, and represents an embryonic individual (redrawn from Horner & Currie, 1994). The next smallest skull is patterned after MOR 548 (also redrawn from Horner & Currie, 1994). The remaining skulls, from left, are patterned after ROM 759, CMN 34825, ROM 5856, and ROM 871, redrawn from Evans (2010). Skulls of *Casuarius* are redrawn from Dodson (1975) and based on (from left) YPM 6208, YPM 1736 (snout region reconstructed), and AMNH 3870. Maximum skull lengths for *Parasaurolophus*, *Corythosaurus*, and *Casuarius* are 745 mm (ROM 768), 750 mm (ROM 792) and 200 mm, respectively (Dodson, 1975; Evans, 2010). Scale bars equal 10 cm.

Based on CT scan data, we reconstruct RAM 14000 as lacking an S-loop in the proximal portion of the nasal passages (Fig. 9), an ontogeny-independent synapomorphy that occurs in most post-embryonic lambeosaurins (Evans & Reisz, 2007; Evans, Ridgely & Witmer, 2009). Additionally, there is no solid, fin-like caudal extension of the crest, found in all juvenile and adult lambeosaurins for which CT scan data are available (Evans, Ridgely & Witmer, 2009). Relative to *Velafrons coahuilensis*, RAM 14000 lacks the unique “kinked” squamosal morphology of that taxon (Gates et al., 2007). In all juvenile and adult lambeosaurins for which the feature is known, the caudolateral process of the premaxilla is moderately to extremely angled at its contact with the maxilla, rather than straight as in RAM 14000 (Fig. 7A; a feature otherwise found in *Parasaurolophus*). Finally, RAM 14000 shows accelerated development of some features relative to known lambeosaurins (outlined below; Fig. 28). There are thus no firm characters to identify RAM 14000 as a lambeosaurin.

Previous authors have identified a suite of characteristics that unite parasaurolophins (*Charonosaurus* and *Parasaurolophus*), which can also be potentially evaluated in RAM 14000 (characters that are not preserved in the specimen are not considered here). These include: (1) a massive frontal platform extending caudally at least to the level of the supratemporal fenestrae; (2) thickening of the dorsal surface of the postorbital in adults to form a promontorium; and (3) an expanded distal head of the fibula (Godefroit, Alifanov & Bolotsky, 2004; Evans & Reisz, 2007; Evans, Reisz & Dupuis, 2007; Prieto-Márquez, 2010). Characters 1 and 2 are intimately linked with the development of the massive crest (at least in *Parasaurolophus*, where crest morphology is known, and presumably also in *Charonosaurus*). RAM 14000 lacks these features, but their absence is not surprising in light of the crest’s incipient development in this specimen. The distal end of the fibula is slightly expanded in RAM 14000, but not to the degree seen in *P. cyrtocristatus* or *C. jiayinensis* (Ostrom, 1963; Godefroit, Bolotsky & Alifanov, 2001). However, this feature also occurs to a lesser degree in *Corythosaurus intermedius* and *Hypacrosaurus stebingeri* (Prieto-Márquez, 2010), and thus cannot be considered taxonomically significant in RAM 14000. Other important characters, such as the number of cervical vertebrae, relative length of metacarpal V, and the participation of the parietal in the occiput, cannot be determined in RAM 14000.

Based on a referred juvenile braincase (CMN 8502), Evans and colleagues (2007) identified several features of the skull roof that they hypothesized to be relatively consistent through ontogeny in *Parasaurolophus*, at least for the sample known at that time. These included: (1) frontal with a thick and steeply angled nasal articular surface; (2) frontals comparatively short; (3) frontals with a poorly developed median cleft at rostral-most extent; (4) rostral processes of frontal meet at broad and obtuse angle in dorsal view; and (5) olfactary depression offset ventrally from roof of cerebral fossa. Characters 1, 2, 4, and 5 are demonstrably absent in RAM 14000, and character 3 cannot be evaluated. Arguably, all of the characters (particularly character 1) are related to the development of the enlarged bony crest supported by the frontals. We thus attribute their absence in RAM 14000, an extremely young individual in which the crest is only incipient, to ontogenetic effects.

Prieto-Márquez (2010) also identified several unambiguous synapomorphies from his dataset that unite *Parasaurolophus* species. Unfortunately, these are either not preserved in RAM 14000 (number of teeth per alveolus at mid-dentary; morphology of deltoid ridge of scapula; proportions of ulna) or are widely distributed across lambeosaurines (proportions of humerus). *Parasaurolophus* is also reconstructed as having an extreme lateroventral extension of the supraacetabular process of the ilium (Fig. 19C), lacking in RAM 14000 (Figs. 18B and 19D). However, this character is ontogenetically dependent (Guenther, 2009), and even variable among *Parasaurolophus* species (much more prominent in *P. cyrtocristatus* specimen FMNH P 27393 than in *P. walkeri* specimen ROM 768). Thus, its absence in RAM 14000 is not unexpected, nor is it of taxonomic consequence.

Although RAM 14000 does not preserve major, previously recognized autapomorphies for *Parasaurolophus*, several cranial features strongly support referral to this taxon. Most significantly, the caudal edge of the caudolateral process of the premaxilla is interpreted as nearly straight along its entire length (Fig. 7A), a feature also found in all species of *Parasaurolophus*. In every other lambeosaurine of all ontogenetic stages for which the character can be determined, the edge is moderately to strongly kinked. The feature may be associated at least in part with the development of the S-loop in the nasal passages, another feature lacking in RAM 14000 and presumed absent in *Parasaurolophus*, based on CT-scan data (Sullivan & Williamson, 1999). Additionally, the nasal passages fill nearly the entire crest in RAM 14000 (Figs. 9A and 11C), as in *Parasaurolophus* but unlike the condition in lambeosaurins (adults and juveniles alike).

Morphology of the jugal is also informative in RAM 14000, with a relatively long and slender quadrate process that, in concert with the postorbital process, bounds a narrow infratemporal fenestra (width:length ratio = 0.3; Fig. 7). This morphology is also consistently seen in adult *Parasaurolophus* (*P. tubicen*, NMMNH P-25100, PMU.R1250; *P. walkeri*, ROM 768; *P. species*, UMNH VP 16666, UCMP 143270; Fig. 28). A narrow infratemporal fenestra also occurs variably in subadults and adults of other lambeosaurines (e.g., *Hypacrosaurus altispinus*, CMN 8501; *Kazaklambia convincens*, PIN 2230; *Velafrons coahuilensis*, CPC-59), but never in combination with a narrow quadrate process of the jugal. Furthermore, the quadrate process is distinctly constricted (Fig. 7), so that the ventral border is slightly concave along its entire length. This feature occurs in other *Parasaurolophus* specimens (*P. tubicen*, PMU.R1250; *P. walkeri*, ROM 768; UMNH VP 16666). Some other lambeosaurines have a similar concave border (e.g., *Lambeosaurus lambei*, CMN 2869), but never in combination with other features. We also note that the quadrate process on the jugal of *Kazaklambia convincens* expands caudally, contrasting with the comparatively uniform width seen in RAM 14000 and other juvenile lambeosaurines. The impression of the jugal on the right side shows a narrow, triangular extension of the maxillary process between the maxilla and lacrimal (Fig. 13B), also found only in *Parasaurolophus* (e.g., ROM 768; Fig. 14D). Thus, although individual features of the jugal in RAM 14000 are found in various lambeosaurines, the combination of features is exclusive to *Parasaurolophus*.

Within the Kaiparowits Formation of Utah, three hadrosaurid taxa are known: the hadrosaurines *Gryposaurus monumentensis* and *Gryposaurus* sp., as well as the lambeosaurine *Parasaurolophus* sp. (Gates et al., 2013; Weishampel & Jensen, 1979; Gates & Sampson, 2007). The known Kaiparowits Formation adult material is most similar to *Parasaurolophus cyrtocristatus*, but some differences in skull morphology suggest that the specimens may represent a distinct but closely related species or a different ontogenetic stage relative to the *P. cyrtocristatus* holotype specimen (Gates et al., 2013). This issue is currently under study (TA Gates and DC Evans, pers. comm. to A Farke, 2012). Of eight adult lambeosaurine skulls from the Kaiparowits Formation (BYU 2467; UMNH VP 16394, 16666, 16689, two unnumbered; RAM unnumbered; UCMP 143270), all are referable to *Parasaurolophus* (Gates et al., 2013). Continued collecting may certainly uncover evidence of other taxa, but to date *Parasaurolophus* is the only known lambeosaurine from the Kaiparowits Formation. This circumstantial evidence is also consistent with the referral of RAM 14000 to the genus.

The described species of *Parasaurolophus* are distinguished by autapomorphies of the crest (Sullivan & Williamson, 1999) that had not yet developed in RAM 14000. Thus, we cannot assign RAM 14000 to a particular species based upon morphology.

In summary, the bulk of the evidence—morphological and geological—is most parsimonious with the referral of RAM 14000 to *Parasaurolophus*. Specific autapomorphies for the genus that are lacking in the specimen—such as the unique crest and frontal morphology—are hypothesized to have developed later in ontogeny. Furthermore, the skull of RAM 14000 shows unique morphology relative to known juvenile lambeosaurins.

### Age of RAM 14000

The tibial bone microstructure of RAM 14000 preserves no lines of arrested growth (LAGs) or annuli, suggesting that this animal did not stop, pause, or dramatically slow its growth at any time between hatching and death. As noted in Nesbitt et al. (2013), the absence of LAGs does not necessarily imply that an animal died within its first year of growth, although that is one possibility. LAGs are not visible when they are deposited but later obscured by secondary remodeling, in animals that grow to full size in less than a year but live for several years afterward, or in animals that grow to full size over several years without pausing or stopping (Horner, de Ricqlès & Padian, 1999; Nesbitt et al., 2013).

Secondary remodeling of primary tissues that once preserved LAGs can be eliminated for RAM 14000. Near the mid-diaphysis (section B; Fig. 22), bone tissue strongly resembles that of embryonic and perinatal ornithopods (e.g., Horner & Currie, 1994; Horner, de Ricqlès & Padian, 2000; Horner, Padian & de Ricqlès, 2001; Horner et al., 2009; Werning, 2012). This region extends to a radius consistent with the size of other perinatal lambeosaurines (Horner & Currie, 1994; Horner, de Ricqlès & Padian, 2000). This possible embryonic/perinatal tissue is not remodeled by secondary osteons, nor is any of the tissue external to it. Because of this, we are confident that the section represents an unobscured record of growth from a time near birth to death and that no LAGs are missing.

We also find it unlikely that RAM 14000 lacks LAGs because *Parasaurolophus* finished growth in less than a year. All four of the other hadrosaurids that have been examined histologically [*Telmatosaurus* (Benton et al., 2010), *Maiasaura* (Horner, de Ricqlès & Padian, 2000; Horner, Padian & de Ricqlès, 2001), *Hypacrosaurus* (Horner & Currie, 1994; Horner, de Ricqlès & Padian, 1999; Cooper et al., 2008), and *Edmontosaurus* (Reid, 1985)] exhibit several LAGs in the cortices of adult limb bones. Because LAGs are deposited annually in vertebrates (Castanet, 1985; Castanet, 1986–1987; Castanet & Naulleau, 1985; Francillon-Vieillot et al., 1990), this suggests that hadrosaurids required more than one year to reach full size. The presence of several cortical LAGs in related taxa also suggests that large hadrosaurids did not grow over several years without stopping, though in the absence of samples from subadult or adult specimens, this possibility cannot be excluded for *Parasaurolophus*.

The histology of RAM 14000 excludes some broader age categories. The section studied here preserves some possible embryonic or perinatal tissues, but it has clearly deposited a significant amount of tissue external to these. Most of this tissue is mature enough to show primary osteons, indicating that some time has passed since deposition of the initial woven “scaffolding” (Stein & Prondvai, 2013). Additionally, more organized bone microstructure and a larger parallel-fibered component of the bony laminae suggests slower bone depositional rates in the outer cortex relative to the inner cortex of RAM 14000 (Stein & Prondvai, 2013). Embryos, perinates, and very young juvenile hadrosaurids exhibit only woven bone (Horner & Currie, 1994; Horner, de Ricqlès & Padian, 2000), so RAM 14000 does not likely belong to these age categories.

Despite relative slowing of growth between the inner and outer regions of the cortex, RAM 14000 was still growing actively at the time of death. It does not exhibit the LAGs or secondary remodeling of the mid-diaphyseal cortex of subadult or adult hadrosaurids (Horner, de Ricqlès & Padian, 1999; Horner, de Ricqlès & Padian, 2000; Horner, Padian & de Ricqlès, 2001), and certainly not the external fundamental system observed in senescent, large-bodied archosaurs (e.g., Woodward, Horner & Farlow, 2011). Therefore, we feel it is also unlikely that RAM 14000 is a subadult or senescent individual. Given that RAM 14000 is not likely a perinate or a subadult, we hypothesize it to be a large juvenile.

The only published histological section sampled from the long bones of a juvenile lambeosaurine is from the femur of MOR 548, a *Hypacrosaurus stebingeri* nestling. This material was described briefly by Horner & Currie (1994) and is currently being redescribed by Horner and his students as part of a larger study of *Hypacrosaurus* growth and ontogeny (JR Horner, pers. comm. to S Werning, 2013). The femur of MOR 548 is approximately 23 cm long (∼2.5 cm diameter; JR Horner, pers. comm. to S Werning, 2013); smaller than RAM 14000 (325 mm). As reported in Horner & Currie (1994), much of the cortex comprises woven bone organized around primary vascular canals. The published image shows a looser compacta relative to RAM 14000, but images of the full cross-section show a great deal of similarity in terms of the organization and patterning of primary osteons and compactness of the bone in the outer cortex (JR Horner, pers. comm. to S Werning, 2013). No LAGs were reported for MOR 548 (Horner & Currie, 1994).

The bone microstructure of an ontogenetic series of the saurolophine *Maiasaura* has also been described (Horner, de Ricqlès & Padian, 2000). RAM 14000 is intermediate in size between the *Maiasaura* juveniles YPM-PU-22472 and MOR-005JV in size (18 cm and 50 cm femur length, respectively; Horner, de Ricqlès & Padian, 2000) and compares well histologically to *Maiasaura* juveniles in most respects. Horner and colleagues note primary osteons with distinct/organized lamellae surrounding the vessels. These primary canals are most commonly longitudinal canals arranged in parallel circumferential rows, but also in laminar and even plexiform patterns. LAGs are rare in juveniles, despite being “animals of considerable size” (Horner, de Ricqlès & Padian, 2000; p. 119), although a LAG occurs in some elements of MOR-005JV (ibid.). RAM 14000 differs from *Maiasaura* juveniles in that it does not exhibit secondary osteons at the mid-diaphysis, though they occur in the more proximal section.

Given that RAM 14000 was clearly still growing at the time of its death, and that the skeletal morphology and bone microstructure are similar to juveniles of other hadrosaurids, we hypothesize that RAM 14000 was a large juvenile. Cooper et al. (2008) estimated growth curves for *Hypacrosaurus* based on LAG circumferences throughout the ontogeny of MOR 549, an adult. Using their models, we reconstruct an age of ∼1 year for juveniles the size of MOR 548, though again, no LAGs were reported by Horner & Currie (1994). A single LAG was reported in some elements of MOR-005JV (Horner, de Ricqlès & Padian, 2000), a juvenile *Maiasaura* that showed similar histology to RAM 14000. Because no LAGs occur in the similarly sized RAM 14000, we tentatively suggest that the animal was under a year of age at the time of death. However, we note that the number of hadrosaurids with good ontogenetic sampling is still very low (only *Maiasaura* and *Hypacrosaurus*), and so our estimate will need revision if future studies show that hadrosaurids sustained uninterrupted high growth rates for longer than the first year of growth.

If our estimates of age and size for RAM 14000 are correct, *Parasaurolophus* must have experienced extremely rapid growth rates. Our results suggest that RAM 14000 reached 25–32% of adult size (based on total body length length and skull length, respectively) in less than a year. Growth curves based on estimates of circumference and mass (as derived from circumference), have been modeled for *Hypacrosaurus* (Cooper et al., 2008) and *Maiasaura* (Erickson, Rogers & Yerby, 2001) Because we lack histological samples from any adult *Parasaurolophus* specimens, we cannot construct growth curves directly comparable to those estimated for *Hypacrosaurus* and *Maiasaura*. However, our estimates of growth (25–32% of adult size in less than a year) are reasonable based on the ontogeny of femoral length reconstructed for both *Maiasaura* and *Hypacrosaurus*.

MOR-005JV, a juvenile *Maiasaura*, was estimated to be one year old at time of death by Erickson, Rogers & Yerby (2001). That individual had a femoral length half that of MOR-005A (50 cm vs. 100 cm; Horner, de Ricqlès & Padian, 2000), an adult specimen estimated to be six years old at time of death (Erickson, Rogers & Yerby, 2001). MOR 548, a juvenile *Hypacrosaurus* approximately 1 year old (see above) had a femur of 23 cm, whereas the adult MOR 549 had a femur 102 cm long (Horner, de Ricqlès & Padian, 1999). In light of similarly rapid first-year growth in these other hadrosaurids, our assessment for *Parasaurolophus* is reasonable.

### Ontogeny in *Parasaurolophus*

Accepting the identification of RAM 14000 as a juvenile *Parasaurolophus*, several notable ontogenetic changes can be inferred for the skull and postcrania in this taxon. Some of these are consistent with previous reports on other lambeosaurines, but others are exclusive to *Parasaurolophus*. Because the following discussion includes at least three different species (*P. walkeri*, *P. cyrtocristatus*, and *P. tubicen*), we caution that some ontogenetic changes may be more phylogenetically restricted than indicated here. Nonetheless, broad similarities across *Parasaurolophus* species imply that many changes are universal to the taxon.

The crest of RAM 14000 differs from that of all known adult *Parasaurolophus* in several important ways. First, the crest in RAM 14000 is restricted to a low eminence rather than an elongated, curved tube that overhangs the braincase (Figs. 11A and 11C) . Second, the crest in RAM 14000 is bordered caudally and at its apex by the nasal. Although the exact sutural relations of adult *Parasaurolophus* are controversial (e.g., differing reconstructions in Weishampel & Jensen, 1979; Fig. 2, versus Sullivan & Williamson, 1999; Fig. 5), it seems likely that the nasal formed only a small portion of the ventral margin of the crest in adult individuals (Sullivan & Williamson, 1999). Thus, the already minimal contribution of the nasal was further minimized through ontogeny. Third, the premaxilla-nasal fontanelle is open, whereas it is completely closed in all other, ontogenetically older specimens.

These differences between the crests of juvenile and adult *Parasaurolophus* are intimately tied to ontogenetic changes in the braincase. The frontal of *Parasaurolophus* thickened and achieved a nearly vertical contact with the nasal only in later ontogenetic stages. At the latest, this occurred by the time the individual reached half of adult skull size (Evans, Reisz & Dupuis, 2007). Finally, a broad nasal-frontal suture also only occurred at half maximum skull size. In all of these details, where known, RAM 14000 is more similar to juveniles of most lambeosaurin species than to subadult or adult *Parasaurolophus*.

Based on reconstructions of the nasal passages in *Parasaurolophus* (Fig. 11), RAM 14000 indicates that several important transformations occurred as the crest elongated. The lateral diverticulum exhibits perhaps the most notable changes. In the smallest juvenile condition (Fig. 11C), the diverticulum completely obscures the main airway in lateral view. In adults (*P. walkeri*, *P. tubicen*, and *P. cyrtocristatus*; Fig. 11A), the main airway greatly exceeded the extent of the lateral diverticulum, as well as bounding the diverticulum dorsally and ventrally. Additionally, the lateral diverticulum is reconstructed as a single blind chamber in adult *P. cyrtocristatus*, whereas it is clearly looped in young juveniles (however, a looped lateral diverticulum has been reconstructed for *P. tubicen*; Sullivan & Williamson, 1999). Additional information (particularly for adult *Parasaurolophus* from the Kaiparowits Formation) may revise this reconstructed sequence. In any case, juvenile *Parasaurolophus* differ markedly in most aspects of their nasal passages from all known adult *Parasaurolophus* as well as from lambeosaurins of all ontogenetic stages. The only major feature that appears to be constant is the lack of an S-loop; *Parasaurolophus* lacks this feature throughout ontogeny, whereas lambeosaurins retain the feature throughout ontogeny (Evans, Ridgely & Witmer, 2009).

The extent of the contributions of the nasal and premaxillae to the crest in *Parasaurolophus* has been a long-standing problem (summarized in Sullivan & Williamson, 1999). Based on the new information from RAM 14000 and comparison with lambeosaurins, we offer some new observations. In lambeosaurins, the relationships of different sections of the nasal passages (e.g., lateral diverticulum) and the surrounding bones (premaxillae and nasals) are relatively invariant through ontogeny. For instance, the nasal bounds the caudal edge of the lateral diverticulum in juvenile (ROM 759) and subadult (CMN 34825) *Corythosaurus* (Evans, Ridgely & Witmer, 2009). A similar relationship exists between the nasal and the lateral diverticulum in RAM 14000. Unlike lambeosaurins, adult *Parasaurolophus* have a much more extensive lateral diverticulum (occupying up to half the length of the crest; e.g., Fig. 11A). However, the most recent interpretation of the crest sutures require that the lateral diverticulum, particularly at its caudal end, be enclosed nearly exclusively by the premaxillae (Sullivan & Williamson, 1999). In contrast, Weishampel (1981b) proposed that the nasals in *P. walkeri* (ROM 768) reached to the mid-length of the crest (see Fig. 2H in that paper). This is roughly coincidental with the extent of the lateral diverticula in *P. walkeri*. We thus summarize two alternative hypotheses: (1) the relationships between the bony elements and the nasal passages were highly plastic through ontogeny in *Parasaurolophus*, due in part to its massive crest, and the crest predominantly is composed of the premaxillae; or (2) the nasal forms a major portion of the crest. We speculate that the latter hypothesis is most likely, based on the lateral diverticulum. Unfortunately, sutures are ambiguous in many skulls of adult *Parasaurolophus* due to crushing or fusion, and thus a rigorous test of the hypothesis will require description of better material and other ontogenetic stages.

The morphology of RAM 14000 also implies that several features of the skull were relatively invariant in *Parasaurolophus* throughout ontogeny. The narrow infratemporal fenestra, constrained by a tightly angled jugal, occurs at all ontogenetic stages. The shape of the oral margin of the premaxilla is also relatively unchanged through ontogeny. Finally, the absence of an S-loop also appears to be invariant throughout ontogeny.

Ontogenetic changes in the postcrania of hadrosaurids are well-documented (Horner & Currie, 1994; Dilkes, 2001; Suzuki, Weishampel & Minoura, 2004; Guenther, 2009; Kilbourne & Makovicky, 2010). The patterns in RAM 14000 and *Parasaurolophus*, both for limb proportions and overall morphology, generally are consistent with observations from other taxa, particularly the lambeosaurine *Hypacrosaurus stebingeri*. Notably, the distal expansion of the ischium is minimal in RAM 14000 (unlike adult individuals of *P. cyrtocristatus*). Similar ontogenetic patterns in the ischium occur in *H. stebingeri* (Horner & Currie, 1994), suggesting that this change is generalized across lambeosaurines with the feature. The most dramatic changes are seen in the ilium, particularly in the reduced size of the supraacetabular process relative to that in adult *Parasaurolophus* (Figs. 19C and 19D). Again, this pattern is probably generalized across hadrosaurids (Guenther, 2009). The humerus:femur ratio is approximately the same in RAM 14000 and adult *Parasaurolophus* ROM 768 and FMNH P 27393 (0.53, 0.50, and 0.51, respectively), but the femur:fibula ratio differs more strongly (1.14 and 1.24 in RAM 14000 and FMNH P 27393, respectively; no complete tibias are known for adult *Parasaurolophus*). Thus, the portion of the leg below the knee joint is slightly longer in the younger animal. This may differ from the condition in *Alligator mississippiensis*, which shows isometry over the ontogeny of the fibula relative to the femur (Livingston et al., 2009). Both *Alligator* and *Parasaurolophus* seem to maintain a consistent humerus:femur ratio over ontogeny. Interestingly, this differs from strong negative allometry seem in the latter ratio for *Massospondylus*, although the sample in this case is much larger (Reisz et al., 2005). A broader sample of associated limb elements is needed to assess ontogenetic changes in limb proportions in hadrosaurids relative to other archosaurs.

### Cranial functional morphology

The rhamphotheca on the upper jaw resulted in a minor reduction in bite force at the tip of the beak, relative to the condition without a rhamphotheca. Although this arguably enforced a slight limitation on the type of food items that could be cropped and ingested, a rhamphotheca would also have had some potential benefits. In particular, the expanded keratinous structure would have increased the area available for cropping, and thus the potential volume of food taken in per bite. Additionally, the rhamphotheca may have allowed the hadrosaur to more efficiently crop plants at ground level, by moving the bite point closer to the ground without having to bend the neck. The effect of a rhamphotheca upon mastication is a topic worthy of additional exploration.

As expected by its smaller size and shorter airway, the crest of RAM 14000 produced a higher resonant frequency than did the crests of adults (assuming that the structure was indeed used in sound production). If such vocalizations played a role in the social behavior of *Parasaurolophus*, perhaps in distinguishing different age categories, (Weishampel, 1981a; Evans, Ridgely & Witmer, 2009), the vastly different frequencies of adult and juvenile animals would have been easily distinguishable (Table 11).

### Heterochrony in hadrosaurids and other ornithischians

Heterochrony—variation in developmental timing of the appearance of anatomical features relative to the ancestral condition (e.g., Gould, 1977; Alberch et al., 1979; Klingenberg, 1998; Smith, 2001)—presumably played an important part in the evolution of lambeosaurine crests. A robust assessment of crest heterochrony requires knowledge of the extent of crest development at given sizes and absolute ages for several taxa, in addition to their stratigraphic ranges and phylogenetic relationships. Estimates of absolute age for fossil taxa are only obtainable from skeletochronological assessments of bone histology. Unfortunately, despite much higher taxonomic diversity within Ankylopollexia and especially within Hadrosauridae, the vast majority of ornithopods sampled for histological study fall outside Ankylopollexia (Werning, 2012). Prior to this study, only four hadrosaurids had been sampled: *Telmatosaurus* (Benton et al., 2010), *Maiasaura* (Horner, de Ricqlès & Padian, 2000; Horner, Padian & de Ricqlès, 2001), *Hypacrosaurus* (Horner & Currie, 1994; Horner, de Ricqlès & Padian, 1999; Cooper et al., 2008), and *Edmontosaurus* (“*Anatosaurus*”; Reid, 1985). Of these, the only lambeosaurine is *Hypacrosaurus*, a lambeosaurin. Additionally, only the histology of *Maiasaura* has been studied throughout ontogeny (Horner, de Ricqlès & Padian, 2000; Horner, Padian & de Ricqlès, 2001), and growth curves have been estimated only for *Maiasaura* (Erickson, Rogers & Yerby, 2001) and *Hypacrosaurus* (Cooper et al., 2008). Thus, the skeletochronological data necessary to link skull size, body size, and crest development with age are virtually nonexistent for lambeosaurines. This is especially unfortunate given that the phylogeny (e.g., Evans & Reisz, 2007; Gates et al., 2007; Prieto-Márquez, 2010) and stratigraphic context (e.g., Ryan & Evans, 2005; Mallon et al., 2012) of hadrosaurids is increasingly well resolved.

Embryonic and post-hatchling material (Horner & Currie, 1994) necessarily imply extremely young age (∼a few months at most) for such specimens. To date, only the holotype specimen of *Hypacrosaurus stebingeri*, MOR 549, has been aged (Horner, de Ricqlès & Padian, 1999; Cooper et al., 2008), with an estimate of approximately 13 years old. This specimen is the only described adult specimen for *H. stebingeri*, and unsurprisingly has the largest crest of any specimen. Nonetheless, the structure is still relatively modest in size relative to the largest crests seen in specimens of *Corythosaurus casuarius* or *Hypacrosaurus altispinus*. This may reflect taxonomic differences, individual variation, sexual dimorphism, or another factor, but these hypotheses cannot be tested without a larger sample.

Extrapolating from the growth curve presented for *H. stebingeri*, and assuming that sexual maturity occurred at or near the growth curve inflection (Erickson et al., 2007; Lee & Werning, 2008), sexual maturity occurred in this species at two to three years of age (Cooper et al., 2008). The reconstructed mean femoral length at this point was 450 mm. This is slightly smaller than the femoral lengths associated with juvenile skeletons referred to *H. stebingeri* (590 mm and 522 mm for AMNH 5340 and 5461, respectively; Lull & Wright, 1942; Evans, 2010). In both cases, the crest is only barely developed, suggesting that crest development in *H. stebingeri* did not occur until after the onset of sexual maturity but well before the animal reached full adult size. Additional histological work is needed to test this hypothesis. Assuming that RAM 14000 was still in its exponential growth phase (pre-inflection), a reasonable assumption given its bone microstructure, it had not yet reached sexual maturity despite already initiating crest development.

Using skull length as a rough proxy for ontogenetic age, it is clear that *Parasaurolophus* initiated externally visible crest development at a much earlier point than did lambeosaurins (∼30% maximum skull size versus ∼50% maximum skull size exclusive of the crest; Fig. 28). Juvenile lambeosaurins nearly twice the size of RAM 14000 have far more subdued crests relative to the rest of the skull; a similar pattern is seen for the potentially basal lambeosaurine *Kazaklambia convincens*. This could result from different life history parameters (e.g., differences in overall growth rate or the onset of sexual maturity), but we suggest it is more likely related to the larger and more “extreme” nature of the crest in *Parasaurolophus* versus lambeosaurins. In other words, the crest had to begin growth at an earlier stage in order to achieve its full extent. Within a standard terminological framework for heterochrony, the earlier and more extreme final development of the crest in *Parasaurolophus* relative to lambeosaurins is a classic example of peramorphosis (*sensu* Alberch et al., 1979). Assuming that *Kazaklambia convincens* is a basal lambeosaurine (Bell & Brink, in press), lambeosaurins such as *Corythosaurus* retained the ancestral condition of crest development occurring relatively late in ontogeny (∼50% maximum skull size). *Parasaurolophus*, relative to the ancestral condition, thus shows the peramorphic phenomena of predisplacement (related to early onset of growth of the crest) as well as probable hypermorphosis (continued, extreme growth of the crest).

Despite the differences in crest development between lambeosaurins and *Parasaurolophus*, lambeosaurine hadrosaurids fit an overall pattern of relatively early development of bony cranial ornamentation in ornithischian dinosaurs. This contrasts with birds that initiate crest growth only as the animal reaches nearly full adult size, such as the cassowary (Fig. 28; Dodson, 1975). Two potential factors may contribute to these differences. First, among neornithines, sexual maturity occurs well after somatic maturity (roughly equivalent to full adult body size). This condition contrasts with that of non-avian dinosaurs which apparently achieved sexual maturity well before somatic maturity (Erickson et al., 2007; Lee & Werning, 2008). Thus, if cranial crests in ornithischians had some species-specific display function—whether for species recognition, sexual selection, or any related use—it is intuitive that the structures appeared before the animal reached full adult body size, and conversely for neornithines.

A second important factor considers the integration of cranial ornamentation into the overall skull in ornithischians versus avians. In birds such as cassowaries and hornbills, the massive casques are simple “add-ons” to the overall skull, formed strictly of a bony core without major involvement of respiratory or muscular systems (Rothschild, 1900; A Farke, personal observation). By contrast, the crests of hadrosaurids are intimately integrated with the respiratory system, by virtue of the airway passing through the crest (Fig. 11). Thus, the crest *had* to form early in development, simply so that the animals could continue to breathe. Similar constraints may have affected the frills of ceratopsians, at least part of which supported jaw musculature (Rieppel, 1981). However, this does not necessarily explain the early development of horns in ceratopsids (Horner & Goodwin, 2006), or nodes and spikes in pachycephalosaurs (Horner & Goodwin, 2009; Schott et al., 2011), structures that seem to be decoupled from more “utilitarian” aspects of the skull. Here, timing of sexual maturity may have played a role. We thus hypothesize that development of different structures was subject to different constraints depending upon their function and location.

## Conclusions

RAM 14000 represents the smallest and most complete *Parasaurolophus* specimen described to date and illustrates a unique juvenile morphology of this taxon relative to other lambeosaurine dinosaurs. Based on histology of the tibia, RAM 14000 exhibits no lines of arrested growth and thus was likely less than a year old at the time of death. Notably, *Parasaurolophus* initiated crest development at a much smaller body size (and presumably younger age) than did lambeosaurin lambeosaurines. At least in part, this is probably because of the extreme morphology of the crest in *Parasaurolophus*, which required a longer period of development.

The timing of the onset of ornamentation development varies dramatically across amniotes, a topic that deserves considerably more attention. This timing is probably influenced by life history traits such as the timing of reproductive maturity, functional demands upon the skull, and phylogenetic history. As a group, lambeosaurine hadrosaurids initiated crest growth well before reaching adult size (between 25 and 50 percent maximum skull length), a condition shared with most other ornithischian dinosaurs with cranial ornamentation. This may result from the intimate association of the ornamentation with essential functional complexes such as the nasal passages (in the case of hadrosaurids) or musculature (in the case of ceratopsians). If cranial ornamentation played at least some role in sexual selection and/or species recognition, early reproductive maturity may also be related to the precocious development of such ornamentation. Understanding these attributes in dinosaurs requires the documentation of more juvenile specimens with associated skeletochronological data, as well as documentation of patterns in extant species.

## Supplemental Information

10.7717/peerj.182/supp-1 Article S1 Full details on data for RAM 14000 uploaded to Figshare

10.7717/peerj.182/supp-2 Figure S1 Interactive reconstruction of skull, nasal passages, and endocranial cavity of *Parasaurolophus* sp., RAM 14000 This digital reconstruction is based on CT scan data, with the skull is shown in white, the nasal passages in green, and the endocranial cavity in blue. A high-resolution two-dimensional image is included in Fig. 9, and raw data are available from Figshare (Table 1, Article S1).

10.7717/peerj.182/supp-3 Figure S2 Interactive reconstruction of endocranial cavity and endosseous labyrinth of *Parasaurolophus* sp., RAM 14000 This digital reconstruction is based on CT scan data, with the endocranial cavity in blue and the endosseous labyrinth in yellow. A high-resolution two-dimensional image is included in Fig. 15, and raw data are available from Figshare (Table 1, Article S1).

10.7717/peerj.182/supp-4 Figure S3 Interactive reconstruction of right side of skeleton of *Parasaurolophus* sp., RAM 14000 This digital image is based on photogrammetric reconstruction of the specimen. Raw data are available from Figshare (Table 1, Article S1).

10.7717/peerj.182/supp-5 Figure S4 Interactive reconstruction of right humerus of *Parasaurolophus* sp., RAM 14000 This digital image is based on photogrammetric reconstruction of the specimen. Raw data are available from Figshare (Table 1, Article S1).

10.7717/peerj.182/supp-6 Figure S5 Interactive reconstruction of right hind limb of *Parasaurolophus* sp., RAM 14000 This digital image is based on photogrammetric reconstruction of the specimen. Raw data are available from Figshare (Table 1, Article S1).

10.7717/peerj.182/supp-7 Table S1 Representation of elements in skeletons of *Parasaurolophus*

## Additional Information and Declarations

### Field Study Permissions

The following information was supplied relating to ethical approvals (i.e., approving body and any reference numbers): Fieldwork was conducted under United States Department of the Interior Bureau of Land Management Paleontological Resources Use Permit (surface collection permit UT06-001S and excavation permit UT10-006E-Gs).

### Data Deposition

The following information was supplied regarding the deposition of related data: Figshare (listed in Table 1):
http://dx.doi.org/10.6084/m9.figshare.664171

http://dx.doi.org/10.6084/m9.figshare.664167

http://dx.doi.org/10.6084/m9.figshare.692150

http://dx.doi.org/10.6084/m9.figshare.664168

http://dx.doi.org/10.6084/m9.figshare.664169

http://dx.doi.org/10.6084/m9.figshare.691047

http://dx.doi.org/10.6084/m9.figshare.692151

http://dx.doi.org/10.6084/m9.figshare.692152

http://dx.doi.org/10.6084/m9.figshare.664170

http://dx.doi.org/10.6084/m9.figshare.691053

http://dx.doi.org/10.6084/m9.figshare.692153

http://dx.doi.org/10.6084/m9.figshare.692155
MorphoBank (listed in Table 2; http://morphobank.org/permalink/?P836, project p836): M193513, M193511, M283554, M283547, M283550, M283548, M283551, M283553, M283549, M283552, M193514, M193515, M193522, M283543, M283544, M283545, M283546, M151601, M193512, M283539, M283540, M283537, M283541, M283538, M283542.
