# Supplementary material for: Ontogeny in the tube-crested dinosaur Parasaurolophus (Hadrosauridae) and heterochrony in hadrosaurids
Source: PeerJ. 2013 Oct 22;1:e182. doi: 10.7717/peerj.182 (PMC3807589; doi:10.7717/peerj.182)
Supplement: Article S1 [file peerj-01-182-s001.pdf]

Farke, A. A., D. J. Chok, A. Herrero, B. Scolieri, and S. Werning. 2013. Ontogeny in the tube-crested dinosaur *Parasaurolophus* (Hadrosauridae) and heterochrony in hadrosaurids. *PeerJ* 1:e182. http://dx.doi.org/10.7717/peerj.182

Supplemental Article S1. Full details on data for RAM 14000 uploaded to Figshare.

Description: CT scan, 3D segmentation, and 3D surface models for RAM 14000 are available via Figshare. The following pages reproduce the metadata associated with each digital item, exactly as uploaded to Figshare.

Title: CT scan of braincase of *Parasaurolophus* sp. (Hadrosauridae: Dinosauria)
Published URL: http://dx.doi.org/10.6084/m9.figshare.664167
File Name: Braincase_RAM14000.zip

Description: This dataset includes a CT (computed tomography) scan of the partial braincase of RAM 14000, a hadrosaurid dinosaur referred to *Parasaurolophus* sp.

Taxon: *Parasaurolophus* sp. (Lambeosaurinae: Hadrosauridae: Ornithischia: Dinosauria)

Specimen: RAM 14000, Raymond M. Alf Museum of Paleontology, Claremont, California, USA. This catalog number comprises the articulated skull and skeleton of a single individual.

Locality and Horizon: Locality RAM V200921, Grand Staircase-Escalante National Monument, Garfield County, Utah, USA; upper part of middle unit of the Kaiparowits Formation (late Campanian, Cretaceous). Detailed locality data are on file at the RAM and are available to qualified investigators upon request.

Scan Parameters: The specimen was scanned on a Toshiba Aquilion 64 scanner at Pomona Valley Hospital Medical Center, Claremont, California, USA, on December 18, 2012. Scan parameters were set at 120 kV and 400 mA, slice thickness of 0.5 mm, and reconstruction diameter of 140.625 mm, using a standard bone reconstruction algorithm, resulting in an in-plane resolution of 0.274 mm by 0.274 mm per pixel. Each slice is 512 by 512 pixels. Slices were exported in DICOM format, with a total of 230 individual files and a combined file size of 121.1 Mb. The files were compressed into a single ZIP archive, with a file size of 57.1 Mb.

Additional Information: RAM 14000 was collected under United States Department of the Interior Bureau of Land Management Paleontological Resources Use Permit (surface collection permit UT06-001S and excavation permit UT10-006E-Gs). The specimen is accessioned into the permanent collection of the Raymond M. Alf Museum of Paleontology (RAM), Claremont, California, USA.

The Alf Museum kindly requests that any publications resulting from these data acknowledge the museum and cite this dataset as well as the original Farke et al. publication, and that a link to or copy of any resulting publications be forwarded to the museum director and/or curator.

Publication Citation: Farke, A. A., D. J. Chok, A. Herrero, B. Scolieri, and S. Werning. 2013. Ontogeny in the tube-crested dinosaur *Parasaurolophus* (Hadrosauridae) and heterochrony in hadrosaurids. *PeerJ* 1:e182. http://dx.doi.org/10.7717/peerj.182

Title: CT scan of right maxilla of *Parasaurolophus* sp. (Hadrosauridae: Dinosauria)
Published URL: http://dx.doi.org/10.6084/m9.figshare.664168
File Name: Maxilla_RAM14000.zip

Description: This dataset includes a CT (computed tomography) scan of the right maxilla of RAM 14000, a hadrosaurid dinosaur referred to *Parasaurolophus* sp.

Taxon: *Parasaurolophus* sp. (Lambeosaurinae: Hadrosauridae: Ornithischia: Dinosauria)

Specimen: RAM 14000, Raymond M. Alf Museum of Paleontology, Claremont, California, USA. This catalog number comprises the articulated skull and skeleton of a single individual.

Locality and Horizon: Locality RAM V200921, Grand Staircase-Escalante National Monument, Garfield County, Utah, USA; upper part of middle unit of the Kaiparowits Formation (late Campanian, Cretaceous). Detailed locality data are on file at the RAM and are available to qualified investigators upon request.

Scan Parameters: The specimen was scanned on a Toshiba Aquilion 64 scanner at Pomona Valley Hospital Medical Center, Claremont, California, USA, on December 18, 2012. Scan parameters were set at 120 kV and 400 mA, slice thickness of 0.5 mm, and reconstruction diameter of 140.625 mm, using a standard bone reconstruction algorithm, resulting in an in-plane resolution of 0.274 mm by 0.274 mm per pixel. Each slice is 512 by 512 pixels. Slices were exported in DICOM format, with a total of 239 individual files and a combined file size of 125.9 Mb. The files were compressed into a single ZIP archive, with a file size of 56.2 Mb.

Additional Information: RAM 14000 was collected under United States Department of the Interior Bureau of Land Management Paleontological Resources Use Permit (surface collection permit UT06-001S and excavation permit UT10-006E-Gs). The specimen is accessioned into the permanent collection of the Raymond M. Alf Museum of Paleontology (RAM), Claremont, California, USA.

The Alf Museum kindly requests that any publications resulting from these data acknowledge the museum and cite this dataset as well as the original Farke et al. publication, and that a link to or copy of any resulting publications be forwarded to the museum director and/or curator.

Publication Citation: Farke, A. A., D. J. Chok, A. Herrero, B. Scolieri, and S. Werning. 2013. Ontogeny in the tube-crested dinosaur *Parasaurolophus* (Hadrosauridae) and heterochrony in hadrosaurids. *PeerJ* 1:e182. http://dx.doi.org/10.7717/peerj.182

Title: CT scan of left half of skull of *Parasaurolophus* sp. (Hadrosauridae: Dinosauria)
Published URL: http://dx.doi.org/10.6084/m9.figshare.664169
File Name: LeftSkull_RAM14000.zip

Description: This dataset includes a CT (computed tomography) scan of the left half of the skull of RAM 14000, a hadrosaurid dinosaur referred to *Parasaurolophus* sp.

Taxon: *Parasaurolophus* sp. (Lambeosaurinae: Hadrosauridae: Ornithischia: Dinosauria)

Specimen: RAM 14000, Raymond M. Alf Museum of Paleontology, Claremont, California, USA. This catalog number comprises the articulated skull and skeleton of a single individual.

Locality and Horizon: Locality RAM V200921, Grand Staircase-Escalante National Monument, Garfield County, Utah, USA; upper part of middle unit of the Kaiparowits Formation (late Campanian, Cretaceous). Detailed locality data are on file at the RAM and are available to qualified investigators upon request.

Scan Parameters: The specimen was scanned on a Toshiba Aquilion 64 scanner at Pomona Valley Hospital Medical Center, Claremont, California, USA, on December 18, 2012. Scan parameters were set at 120 kV and 400 mA, slice thickness of 0.5 mm, and reconstruction diameter of 229.687 mm, using a standard bone reconstruction algorithm, resulting in an in-plane resolution of 0.45 mm by 0.45 mm per pixel. Each slice is 512 by 512 pixels. Slices were exported in DICOM format, with a total of 531 individual files and a combined file size of 279.6 Mb. The files were compressed into a single ZIP archive, with a file size of 140.2 Mb.

Additional Information: RAM 14000 was collected under United States Department of the Interior Bureau of Land Management Paleontological Resources Use Permit (surface collection permit UT06-001S and excavation permit UT10-006E-Gs). The specimen is accessioned into the permanent collection of the Raymond M. Alf Museum of Paleontology (RAM), Claremont, California, USA.

The Alf Museum kindly requests that any publications resulting from these data acknowledge the museum and cite this dataset as well as the original Farke et al. publication, and that a link to or copy of any resulting publications be forwarded to the museum director and/or curator.

Publication Citation: Farke, A. A., D. J. Chok, A. Herrero, B. Scolieri, and S. Werning. 2013. Ontogeny in the tube-crested dinosaur *Parasaurolophus* (Hadrosauridae) and heterochrony in hadrosaurids. *PeerJ* 1:e182. http://dx.doi.org/10.7717/peerj.182

Title: CT scan of right half of skull and neck of *Parasaurolophus* sp. (Hadrosauridae: Dinosauria)
Published URL: http://dx.doi.org/10.6084/m9.figshare.664170
File Name: RightSkull_RAM14000.zip

Description: This dataset includes a CT (computed tomography) scan of the right half of the skull and neck of RAM 14000, a hadrosaurid dinosaur referred to *Parasaurolophus* sp.

Taxon: *Parasaurolophus* sp. (Lambeosaurinae: Hadrosauridae: Ornithischia: Dinosauria)

Specimen: RAM 14000, Raymond M. Alf Museum of Paleontology, Claremont, California, USA. This catalog number comprises the articulated skull and skeleton of a single individual.

Locality and Horizon: Locality RAM V200921, Grand Staircase-Escalante National Monument, Garfield County, Utah, USA; upper part of middle unit of the Kaiparowits Formation (late Campanian, Cretaceous). Detailed locality data are on file at the RAM and are available to qualified investigators upon request.

Scan Parameters: The specimen was scanned on a Toshiba Aquilion 64 scanner at Pomona Valley Hospital Medical Center, Claremont, California, USA, on October 12, 2011. Scan parameters were set at 120 kV and 350 mA, slice thickness of 0.5 mm, and reconstruction diameter of 300 mm, using a standard bone reconstruction algorithm, resulting in an in-plane resolution of 0.586 mm by 0.586 mm per pixel. Each slice is 512 by 512 pixels. Slices were exported in DICOM format, with a total of 637 individual files and a combined file size of 335.5 Mb. The files were compressed into a single ZIP archive, with a file size of 174.6 Mb.

Additional Information: RAM 14000 was collected under United States Department of the Interior Bureau of Land Management Paleontological Resources Use Permit (surface collection permit UT06-001S and excavation permit UT10-006E-Gs). The specimen is accessioned into the permanent collection of the Raymond M. Alf Museum of Paleontology (RAM), Claremont, California, USA.

The Alf Museum kindly requests that any publications resulting from these data acknowledge the museum and cite this dataset as well as the original Farke et al. publication, and that a link to or copy of any resulting publications be forwarded to the museum director and/or curator.

Publication Citation: Farke, A. A., D. J. Chok, A. Herrero, B. Scolieri, and S. Werning. 2013. Ontogeny in the tube-crested dinosaur *Parasaurolophus* (Hadrosauridae) and heterochrony in hadrosaurids. *PeerJ* 1:e182. http://dx.doi.org/10.7717/peerj.182

Title: Segmentation data for braincase of *Parasaurolophus* sp. (Hadrosauridae: Dinosauria)
Published URL: http://dx.doi.org/10.6084/m9.figshare.664171
File Name: RAM14000_braincase_Slicer.zip

Description: This dataset includes segmentation data used to produce 3D digital models for the partial braincase of RAM 14000, a hadrosaurid dinosaur referred to *Parasaurolophus* sp.

Taxon: *Parasaurolophus* sp. (Lambeosaurinae: Hadrosauridae: Ornithischia: Dinosauria)

Specimen: RAM 14000, Raymond M. Alf Museum of Paleontology, Claremont, California, USA. This catalog number comprises the articulated skull and skeleton of a single individual.

Locality and Horizon: Locality RAM V200921, Grand Staircase-Escalante National Monument, Garfield County, Utah, USA; upper part of middle unit of the Kaiparowits Formation (late Campanian, Cretaceous). Detailed locality data are on file at the RAM and are available to qualified investigators upon request.

Source Data: The original CT scan data are provided at http://dx.doi.org/10.6084/m9.figshare.664167

File Information: This archive includes trimmed scan data (Analyze file format, with .img and .hdr file extensions), segmentation labels (NRRD format, with .nrrd file extension), surface models (VTK format, .vtk file extension), a scene preview (PNG format, .png file extension), and a scene information file (MRML file format, .mrml file extension). These files were produced in the open source imaging program 3D Slicer 4.2, and may be opened using this program (available from www.slicer.org). This single ZIP archive contains a total of 16 individual files and a combined file size of 59.7 Mb, compressed to 41.0 Mb. Note that the models are mirrored relative to the original specimen, due to a glitch in the import of the CT scan data.

Additional Information: RAM 14000 was collected under United States Department of the Interior Bureau of Land Management Paleontological Resources Use Permit (surface collection permit UT06-001S and excavation permit UT10-006E-Gs). The specimen is accessioned into the permanent collection of the Raymond M. Alf Museum of Paleontology (RAM), Claremont, California, USA.

The Alf Museum kindly requests that any publications resulting from these data acknowledge the museum and cite this dataset as well as the original Farke et al. publication, and that a link to or copy of any resulting publications be forwarded to the museum director and/or curator.

Publication Citation: Farke, A. A., D. J. Chok, A. Herrero, B. Scolieri, and S. Werning. 2013. Ontogeny in the tube-crested dinosaur *Parasaurolophus* (Hadrosauridae) and heterochrony in hadrosaurids. *PeerJ* 1:e182. http://dx.doi.org/10.7717/peerj.182

Title: Segmentation data for left half of skull of *Parasaurolophus* sp. (Hadrosauridae: Dinosauria)
Published URL: http://dx.doi.org/10.6084/m9.figshare.691047
File Name: RAM14000_leftskull_Slicer.zip

Description: This dataset includes segmentation data used to produce 3D digital models for the left half of the skull of RAM 14000 (nasal passages, endocranial cavity, and skull), a hadrosaurid dinosaur referred to *Parasaurolophus* sp.

Taxon: *Parasaurolophus* sp. (Lambeosaurinae: Hadrosauridae: Ornithischia: Dinosauria)

Specimen: RAM 14000, Raymond M. Alf Museum of Paleontology, Claremont, California, USA. This catalog number comprises the articulated skull and skeleton of a single individual.

Locality and Horizon: Locality RAM V200921, Grand Staircase-Escalante National Monument, Garfield County, Utah, USA; upper part of middle unit of the Kaiparowits Formation (late Campanian, Cretaceous). Detailed locality data are on file at the RAM and are available to qualified investigators upon request.

Source Data: The original CT scan data are provided at http://dx.doi.org/10.6084/m9.figshare.664169

File Information: This archive includes trimmed scan data (Analyze file format, with .img and .hdr file extensions), segmentation labels (NRRD format, with .nrrd file extension), surface models (VTK format, .vtk file extension), a scene preview (PNG format, .png file extension), and a scene information file (MRML file format, .mrml file extension). These files were produced in the open source imaging program 3D Slicer 4.2, and may be opened using this program (available from www.slicer.org). This single ZIP archive contains a total of 11 individual files and a combined file size of 119.4 Mb, compressed to 86.4 Mb. Note that the models are mirrored relative to the original specimen, due to a glitch in the import of the CT scan data.

Additional Information: RAM 14000 was collected under United States Department of the Interior Bureau of Land Management Paleontological Resources Use Permit (surface collection permit UT06-001S and excavation permit UT10-006E-Gs). The specimen is accessioned into the permanent collection of the Raymond M. Alf Museum of Paleontology (RAM), Claremont, California, USA.

The Alf Museum kindly requests that any publications resulting from these data acknowledge the museum and cite this dataset as well as the original Farke et al. publication, and that a link to or copy of any resulting publications be forwarded to the museum director and/or curator.

Publication Citation: Farke, A. A., D. J. Chok, A. Herrero, B. Scolieri, and S. Werning. 2013. Ontogeny in the tube-crested dinosaur *Parasaurolophus* (Hadrosauridae) and heterochrony in hadrosaurids. *PeerJ* 1:e182. http://dx.doi.org/10.7717/peerj.182

Title: Segmentation data for right half of skull and neck of *Parasaurolophus* sp. (Hadrosauridae: Dinosauria)
Published URL: http://dx.doi.org/10.6084/m9.figshare.691053
File Name: RAM14000_rightskull_Slicer.zip

Description: This dataset includes segmentation data used to produce 3D digital models for the right half of the skull and neck of RAM 14000 (including the hyoid), a hadrosaurid dinosaur referred to *Parasaurolophus* sp.

Taxon: *Parasaurolophus* sp. (Lambeosaurinae: Hadrosauridae: Ornithischia: Dinosauria)

Specimen: RAM 14000, Raymond M. Alf Museum of Paleontology, Claremont, California, USA. This catalog number comprises the articulated skull and skeleton of a single individual.

Locality and Horizon: Locality RAM V200921, Grand Staircase-Escalante National Monument, Garfield County, Utah, USA; upper part of middle unit of the Kaiparowits Formation (late Campanian, Cretaceous). Detailed locality data are on file at the RAM and are available to qualified investigators upon request.

Source Data: The original CT scan data are provided at http://dx.doi.org/10.6084/m9.figshare.664170

File Information: This archive includes trimmed scan data (Analyze file format, with .img and .hdr file extensions), segmentation labels (NRRD format, with .nrrd file extension), surface models (VTK format, .vtk file extension), a scene preview (PNG format, .png file extension), and a scene information file (MRML file format, .mrml file extension). These files were produced in the open source imaging program 3D Slicer 4.2, and may be opened using this program (available from www.slicer.org). This single ZIP archive contains a total of 8 individual files and a combined file size of 196.4 Mb, compressed to 190.5 Mb.

Additional Information: RAM 14000 was collected under United States Department of the Interior Bureau of Land Management Paleontological Resources Use Permit (surface collection permit UT06-001S and excavation permit UT10-006E-Gs). The specimen is accessioned into the permanent collection of the Raymond M. Alf Museum of Paleontology (RAM), Claremont, California, USA.

The Alf Museum kindly requests that any publications resulting from these data acknowledge the museum and cite this dataset as well as the original Farke et al. publication, and that a link to or copy of any resulting publications be forwarded to the museum director and/or curator.

Publication Citation: Farke, A. A., D. J. Chok, A. Herrero, B. Scolieri, and S. Werning. 2013. Ontogeny in the tube-crested dinosaur *Parasaurolophus* (Hadrosauridae) and heterochrony in hadrosaurids. *PeerJ* 1:e182. http://dx.doi.org/10.7717/peerj.182

Title: Surface models for braincase of *Parasaurolophus* sp. (Hadrosauridae: Dinosauria)
Published URL: http://dx.doi.org/10.6084/m9.figshare.692150
File Name: BraincaseModels_RAM14000.zip

Description: This dataset includes 3D surface models for the partial braincase of RAM 14000, a hadrosaurid dinosaur referred to *Parasaurolophus* sp.

Taxon: *Parasaurolophus* sp. (Lambeosaurinae: Hadrosauridae: Ornithischia: Dinosauria)

Specimen: RAM 14000, Raymond M. Alf Museum of Paleontology, Claremont, California, USA. This catalog number comprises the articulated skull and skeleton of a single individual.

Locality and Horizon: Locality RAM V200921, Grand Staircase-Escalante National Monument, Garfield County, Utah, USA; upper part of middle unit of the Kaiparowits Formation (late Campanian, Cretaceous). Detailed locality data are on file at the RAM and are available to qualified investigators upon request.

Source Data: The original CT scan and segmentation data are provided at:
http://dx.doi.org/10.6084/m9.figshare.664167
http://dx.doi.org/10.6084/m9.figshare.664171

File Information: This archive includes 3D surface model data in STL format (.stl file extension). Files include bone.stl (bone and matrix), brain_endocast.stl (endocast of cranial cavity), ear_labyrinth.stl (endocast of endosseous labyrinth), and pituitary.stl (endocast of pituitary fossa). These files were produced in the open source imaging program 3D Slicer 4.2 (available from www.slicer.org), and reformatted within the open source program MeshLab (available from www.meshlab.org). All are to scale (i.e., 1 unit on the models equals 1 mm). This single ZIP archive contains a total of 4 individual files and a combined file size of 58.5 Mb, compressed to 31.3 Mb.

Additional Information: RAM 14000 was collected under United States Department of the Interior Bureau of Land Management Paleontological Resources Use Permit (surface collection permit UT06-001S and excavation permit UT10-006E-Gs). The specimen is accessioned into the permanent collection of the Raymond M. Alf Museum of Paleontology (RAM), Claremont, California, USA.

The Alf Museum kindly requests that any publications resulting from these data acknowledge the museum and cite this dataset as well as the original Farke et al. publication, and that a link to or copy of any resulting publications be forwarded to the museum director and/or curator.

Publication Citation: FFarke, A. A., D. J. Chok, A. Herrero, B. Scolieri, and S. Werning. 2013. Ontogeny in the tube-crested dinosaur *Parasaurolophus* (Hadrosauridae) and heterochrony in hadrosaurids. *PeerJ* 1:e182. http://dx.doi.org/10.7717/peerj.182

Title: Surface models for left half of skull of *Parasaurolophus* sp. (Hadrosauridae: Dinosauria)
Published URL: http://dx.doi.org/10.6084/m9.figshare.692151
File Name: LeftSkullModels_RAM14000.zip

Description: This dataset includes 3D surface models for the left half of the skull and endocranial structures of RAM 14000, a hadrosaurid dinosaur referred to *Parasaurolophus* sp.

Taxon: *Parasaurolophus* sp. (Lambeosaurinae: Hadrosauridae: Ornithischia: Dinosauria)

Specimen: RAM 14000, Raymond M. Alf Museum of Paleontology, Claremont, California, USA. This catalog number comprises the articulated skull and skeleton of a single individual.

Locality and Horizon: Locality RAM V200921, Grand Staircase-Escalante National Monument, Garfield County, Utah, USA; upper part of middle unit of the Kaiparowits Formation (late Campanian, Cretaceous). Detailed locality data are on file at the RAM and are available to qualified investigators upon request.

Source Data: The original CT scan and segmentation data are provided at:
http://dx.doi.org/10.6084/m9.figshare.664169
http://dx.doi.org/10.6084/m9.figshare.691047

File Information: This archive includes 3D surface model data in STL format (.stl file extension). Files include left skull.stl (bone and some matrix), left_nasalpassages.stl (endocast of nasal passages), and left_endocast.stl (endocast of left side of endocranial cavity). These files were produced in the open source imaging program 3D Slicer 4.2 (available from www.slicer.org), and reformatted within the open source program MeshLab (available from www.meshlab.org). All are to scale (i.e., 1 unit on the models equals 1 mm). This single ZIP archive contains a total of 3 individual files and a combined file size of 107.6 Mb, compressed to 56.9 Mb.

Additional Information: RAM 14000 was collected under United States Department of the Interior Bureau of Land Management Paleontological Resources Use Permit (surface collection permit UT06-001S and excavation permit UT10-006E-Gs). The specimen is accessioned into the permanent collection of the Raymond M. Alf Museum of Paleontology (RAM), Claremont, California, USA.

The Alf Museum kindly requests that any publications resulting from these data acknowledge the museum and cite this dataset as well as the original Farke et al. publication, and that a link to or copy of any resulting publications be forwarded to the museum director and/or curator.

Publication Citation: Farke, A. A., D. J. Chok, A. Herrero, B. Scolieri, and S. Werning. 2013. Ontogeny in the tube-crested dinosaur *Parasaurolophus* (Hadrosauridae) and heterochrony in hadrosaurids. *PeerJ* 1:e182. http://dx.doi.org/10.7717/peerj.182

Title: Surface model for left half of skull of *Parasaurolophus* sp. (Hadrosauridae: Dinosauria)
Published URL: http://dx.doi.org/10.6084/m9.figshare.692152
File Name: LeftSkullModelHighRes_RAM14000.stl.zip

Description: This dataset includes a 3D surface model for the left half of the skull of RAM 14000, a hadrosaurid dinosaur referred to *Parasaurolophus* sp.

Taxon: *Parasaurolophus* sp. (Lambeosaurinae: Hadrosauridae: Ornithischia: Dinosauria)

Specimen: RAM 14000, Raymond M. Alf Museum of Paleontology, Claremont, California, USA. This catalog number comprises the articulated skull and skeleton of a single individual.

Locality and Horizon: Locality RAM V200921, Grand Staircase-Escalante National Monument, Garfield County, Utah, USA; upper part of middle unit of the Kaiparowits Formation (late Campanian, Cretaceous). Detailed locality data are on file at the RAM and are available to qualified investigators upon request.

Source Data: The original CT scan and segmentation data are provided at: http://dx.doi.org/10.6084/m9.figshare.664169

File Information: This archive includes 3D surface model data in STL format (.stl file extension). Files include LeftSkullModelHighRes_RAM14000.stl (bone and matrix). Endocranial structures have not been segmented, and the labels were generated at a broad threshold so as to include relatively low-density regions of weathered bone and matrix (contrasting with the tighter threshold used for the companion models available at http://dx.doi.org/10.6084/m9.figshare.692151). These files were produced in the open source imaging program 3D Slicer 4.2 (available from www.slicer.org), and reformatted within the open source program MeshLab (available from www.meshlab.org). All are to scale (i.e., 1 unit on the models equals 1 mm). This single ZIP archive contains 1 file with a file size of 48.1 Mb, compressed to 25.1 Mb.

Additional Information: RAM 14000 was collected under United States Department of the Interior Bureau of Land Management Paleontological Resources Use Permit (surface collection permit UT06-001S and excavation permit UT10-006E-Gs). The specimen is accessioned into the permanent collection of the Raymond M. Alf Museum of Paleontology (RAM), Claremont, California, USA.

The Alf Museum kindly requests that any publications resulting from these data acknowledge the museum and cite this dataset as well as the original Farke et al. publication, and that a link to or copy of any resulting publications be forwarded to the museum director and/or curator.

Publication Citation: Farke, A. A., D. J. Chok, A. Herrero, B. Scolieri, and S. Werning. 2013. Ontogeny in the tube-crested dinosaur *Parasaurolophus* (Hadrosauridae) and heterochrony in hadrosaurids. *PeerJ* 1:e182. http://dx.doi.org/10.7717/peerj.182

Title: Surface models for right half of skull of *Parasaurolophus* sp. (Hadrosauridae: Dinosauria)
Published URL: http://dx.doi.org/10.6084/m9.figshare.692153
File Name: RightSkullModels_RAM14000.zip

Description: This dataset includes 3D surface models for the right half of the skull and neck of RAM 14000, a hadrosaurid dinosaur referred to *Parasaurolophus* sp.

Taxon: *Parasaurolophus* sp. (Lambeosaurinae: Hadrosauridae: Ornithischia: Dinosauria)

Specimen: RAM 14000, Raymond M. Alf Museum of Paleontology, Claremont, California, USA. This catalog number comprises the articulated skull and skeleton of a single individual.

Locality and Horizon: Locality RAM V200921, Grand Staircase-Escalante National Monument, Garfield County, Utah, USA; upper part of middle unit of the Kaiparowits Formation (late Campanian, Cretaceous). Detailed locality data are on file at the RAM and are available to qualified investigators upon request.

Source Data: The original CT scan and segmentation data are provided at:
http://dx.doi.org/10.6084/m9.figshare.664170
http://dx.doi.org/10.6084/m9.figshare.691053

File Information: This archive includes 3D surface model data in STL format (.stl file extension). Files include RightSkullModel_RAM14000.stl (bone and matrix) and HyoidRight_RAM14000.stl (isolated right hyoid). These files were produced in the open source imaging program 3D Slicer 4.2 (available from www.slicer.org), and reformatted within the open source program MeshLab (available from www.meshlab.org). All are to scale (i.e., 1 unit on the models equals 1 mm). This single ZIP archive contains a total of 2 individual files and a combined file size of 56.2 Mb, compressed to 30.6 Mb.

Additional Information: RAM 14000 was collected under United States Department of the Interior Bureau of Land Management Paleontological Resources Use Permit (surface collection permit UT06-001S and excavation permit UT10-006E-Gs). The specimen is accessioned into the permanent collection of the Raymond M. Alf Museum of Paleontology (RAM), Claremont, California, USA.

The Alf Museum kindly requests that any publications resulting from these data acknowledge the museum and cite this dataset as well as the original Farke et al. publication, and that a link to or copy of any resulting publications be forwarded to the museum director and/or curator.

Publication Citation: Farke, A. A., D. J. Chok, A. Herrero, B. Scolieri, and S. Werning. 2013. Ontogeny in the tube-crested dinosaur *Parasaurolophus* (Hadrosauridae) and heterochrony in hadrosaurids. *PeerJ* 1:e182. http://dx.doi.org/10.7717/peerj.182

Title: Surface model for right side of the skeleton of *Parasaurolophus* sp. (Hadrosauridae: Dinosauria)
Published URL: http://dx.doi.org/10.6084/m9.figshare.796442
File Name: RightSkeleton_RAM14000.zip

Description: This dataset includes a 3D surface model for the right side of the skeleton from RAM 14000, a hadrosaurid dinosaur referred to *Parasaurolophus* sp.

Taxon: *Parasaurolophus* sp. (Lambeosaurinae: Hadrosauridae: Ornithischia: Dinosauria)

Specimen: RAM 14000, Raymond M. Alf Museum of Paleontology, Claremont, California, USA. This catalog number comprises the articulated skull and skeleton of a single individual.

Locality and Horizon: Locality RAM V200921, Grand Staircase-Escalante National Monument, Garfield County, Utah, USA; upper part of middle unit of the Kaiparowits Formation (late Campanian, Cretaceous). Detailed locality data are on file at the RAM and are available to qualified investigators upon request.

File Information: The original point cloud was reconstructed using photogrammetry, between April 30 and May 2, 2013. Photographs at 2848×4288 pixel resolution were acquired with a Nikon D90 SLR digital camera (Nikon, Inc., Melville, New York, USA) fitted with a Tamron 179D lens (Tamron Co., Ltd., Saitama, Japan), and were resized to 2000×1500 pixels. Ten separate reconstructions were generated, for the ventral, central, and caudal portions of the rib cage (utilizing 15, 24, and 24 photos, respectively), femur (16 photos), tibia and fibula (29 photos), pes (27 photos), pelvic region (21 photos), skull (17 photos), tail (24 photos), and dorsal view of the skeletal block (18 photos). Data were processed using BundlerTools, which in turn uses Bundler 0.4, CMVS, and PMVS2. The resulting raw point cloud was processed further in MeshLab 1.3.0, in which a surface mesh was produced using a Poisson surface reconstruction algorithm (Octree Depth=12, Solver Divide=12, 5 samples per node, Surface Offsetting=1). The mesh was scaled by comparison with measurements of the original specimen (1 unit on the model equals 1 mm), and data were exported in PLY file format. This single ZIP archive contains one file with an original size of 47.6 Mb, compressed to 19.5 Mb.

Additional Information: RAM 14000 was collected under United States Department of the Interior Bureau of Land Management Paleontological Resources Use Permit (surface collection permit UT06-001S and excavation permit UT10-006E-Gs). The specimen is accessioned into the permanent collection of the Raymond M. Alf Museum of Paleontology (RAM), Claremont, California, USA.

The Alf Museum kindly requests that any publications resulting from these data acknowledge the museum and cite this dataset as well as the original Farke et al. publication, and that a link to or copy of any resulting publications be forwarded to the museum director and/or curator.

Publication Citation: Farke, A. A., D. J. Chok, A. Herrero, B. Scolieri, and S. Werning. 2013. Ontogeny in the tube-crested dinosaur *Parasaurolophus* (Hadrosauridae) and heterochrony in hadrosaurids. *PeerJ* 1:e182. http://dx.doi.org/10.7717/peerj.182

Title: Surface model for right humerus of *Parasaurolophus* sp. (Hadrosauridae: Dinosauria)
Published URL: http://dx.doi.org/10.6084/m9.figshare.692155
File Name: RightHumerus_RAM14000.zip

Description: This dataset includes a 3D surface model for the right humerus of RAM 14000, a hadrosaurid dinosaur referred to *Parasaurolophus* sp.

Taxon: *Parasaurolophus* sp. (Lambeosaurinae: Hadrosauridae: Ornithischia: Dinosauria)

Specimen: RAM 14000, Raymond M. Alf Museum of Paleontology, Claremont, California, USA. This catalog number comprises the articulated skull and skeleton of a single individual.

Locality and Horizon: Locality RAM V200921, Grand Staircase-Escalante National Monument, Garfield County, Utah, USA; upper part of middle unit of the Kaiparowits Formation (late Campanian, Cretaceous). Detailed locality data are on file at the RAM and are available to qualified investigators upon request.

File Information: The original point cloud was reconstructed using photogrammetry, on April 13, 2012. 12 color photos at 4000x3000 pixel resolution were acquired with a Nikon CoolPix L22 digital camera, and were resized to 2000x1500 pixels. Data were processed using BundlerTools, which in turn uses Bundler 0.4, CMVS, and PMVS2. The resulting raw point cloud was processed further in MeshLab 1.3.0, in which a surface mesh was produced using a Poisson surface reconstruction algorithm (Octree Depth=10, Solver Divide=9, 1 sample per node, Surface Offsetting=1). Because the original mesh represented a natural mold, normals were inverted to produce a digital cast. The mesh was scaled by comparison with measurements of the original specimen (1 unit on the model equals 1 mm), and data were exported in STL file format. This single ZIP archive contains one file with an original size of 9.3 Mb, compressed to 3.7 Mb.

Additional Information: RAM 14000 was collected under United States Department of the Interior Bureau of Land Management Paleontological Resources Use Permit (surface collection permit UT06-001S and excavation permit UT10-006E-Gs). The specimen is accessioned into the permanent collection of the Raymond M. Alf Museum of Paleontology (RAM), Claremont, California, USA.

The Alf Museum kindly requests that any publications resulting from these data acknowledge the museum and cite this dataset as well as the original Farke et al. publication, and that a link to or copy of any resulting publications be forwarded to the museum director and/or curator.

Publication Citation: Farke, A. A., D. J. Chok, A. Herrero, B. Scolieri, and S. Werning. 2013. Ontogeny in the tube-crested dinosaur *Parasaurolophus* (Hadrosauridae) and heterochrony in hadrosaurids. *PeerJ* 1:e182. http://dx.doi.org/10.7717/peerj.182

Title: Surface model for right hind limb of *Parasaurolophus* sp. (Hadrosauridae: Dinosauria)
Published URL: http://dx.doi.org/10.6084/m9.figshare.796441
File Name: RightHindLimb_RAM14000.zip

Description: This dataset includes a 3D surface model for the right hind limb from RAM 14000, a hadrosaurid dinosaur referred to *Parasaurolophus* sp.

Taxon: *Parasaurolophus* sp. (Lambeosaurinae: Hadrosauridae: Ornithischia: Dinosauria)

Specimen: RAM 14000, Raymond M. Alf Museum of Paleontology, Claremont, California, USA. This catalog number comprises the articulated skull and skeleton of a single individual.

Locality and Horizon: Locality RAM V200921, Grand Staircase-Escalante National Monument, Garfield County, Utah, USA; upper part of middle unit of the Kaiparowits Formation (late Campanian, Cretaceous). Detailed locality data are on file at the RAM and are available to qualified investigators upon request.

File Information: The original point cloud was reconstructed using photogrammetry, between April 30 and May 2, 2013. Photographs at 2848×4288 pixel resolution were acquired with a Nikon D90 SLR digital camera (Nikon, Inc., Melville, New York, USA) fitted with a Tamron 179D lens (Tamron Co., Ltd., Saitama, Japan), and were resized to 2000×1500 pixels. Three separate reconstructions were generated, for the femur (16 photos), tibia and fibula (29 photos), and pes (27 photos). Data were processed using BundlerTools, which in turn uses Bundler 0.4, CMVS, and PMVS2. The resulting raw point cloud was processed further in MeshLab 1.3.0, in which a surface mesh was produced using a Poisson surface reconstruction algorithm (Octree Depth=12, Solver Divide=12, 5 samples per node, Surface Offsetting=1). The mesh was scaled by comparison with measurements of the original specimen (1 unit on the model equals 1 mm), and data were exported in PLY file format. This single ZIP archive contains one file with an original size of 12.2 Mb, compressed to 5.0 Mb.

Additional Information: RAM 14000 was collected under United States Department of the Interior Bureau of Land Management Paleontological Resources Use Permit (surface collection permit UT06-001S and excavation permit UT10-006E-Gs). The specimen is accessioned into the permanent collection of the Raymond M. Alf Museum of Paleontology (RAM), Claremont, California, USA.

The Alf Museum kindly requests that any publications resulting from these data acknowledge the museum and cite this dataset as well as the original Farke et al. publication, and that a link to or copy of any resulting publications be forwarded to the museum director and/or curator.

Publication Citation: Farke, A. A., D. J. Chok, A. Herrero, B. Scolieri, and S. Werning. 2013. Ontogeny in the tube-crested dinosaur *Parasaurolophus* (Hadrosauridae) and heterochrony in hadrosaurids. *PeerJ* 1:e182. http://dx.doi.org/10.7717/peerj.182

Title: Surface model for squamosal of *Parasaurolophus* sp. (Hadrosauridae: Dinosauria)
Published URL: http://dx.doi.org/10.6084/m9.figshare.797519
File Name: RAM14000_Squamosal.zip

Description: This dataset includes 3D surface models for the right squamosal from RAM 14000, a hadrosaurid dinosaur referred to *Parasaurolophus* sp.

Taxon: *Parasaurolophus* sp. (Lambeosaurinae: Hadrosauridae: Ornithischia: Dinosauria)

Specimen: RAM 14000, Raymond M. Alf Museum of Paleontology, Claremont, California, USA. This catalog number comprises the articulated skull and skeleton of a single individual.

Locality and Horizon: Locality RAM V200921, Grand Staircase-Escalante National Monument, Garfield County, Utah, USA; upper part of middle unit of the Kaiparowits Formation (late Campanian, Cretaceous). Detailed locality data are on file at the RAM and are available to qualified investigators upon request.

File Information: The original point cloud was captured using a NextEngine 3D color laser scanner (NextEngine, Inc., Santa Monica, California), in September 2013. A total of 24 individual scans (2 sets of 12 divisions each, with 30 degree rotation of the specimen between scans) were acquired at a resolution of 6,200 points/cm^2. The individual scans were stitched together in ScanStudio HD Pro 1.3.2 (NextEngine, Inc., Santa Monica, California) and fused into a single watertight mesh (0.000254 cm tolerance, 10 pixel texture blending, 0.5 resolution ratio). The resulting mesh had 210,556 points and 323,907 triangles, and is scaled so that 1 unit equals 1 mm. Data were exported in PLY, OBJ, and STL formats, with a total size of 66.1 Mb; these files were compressed into a single ZIP archive, at 21.9 Mb.

Additional Information: RAM 14000 was collected under United States Department of the Interior Bureau of Land Management Paleontological Resources Use Permit (surface collection permit UT06-001S and excavation permit UT10-006E-Gs). The specimen is accessioned into the permanent collection of the Raymond M. Alf Museum of Paleontology (RAM), Claremont, California, USA.

The Alf Museum kindly requests that any publications resulting from these data acknowledge the museum and cite this dataset as well as the original Farke et al. publication, and that a link to or copy of any resulting publications be forwarded to the museum director and/or curator.

Publication Citation: Farke, A. A., D. J. Chok, A. Herrero, B. Scolieri, and S. Werning. 2013. Ontogeny in the tube-crested dinosaur *Parasaurolophus* (Hadrosauridae) and heterochrony in hadrosaurids. *PeerJ* 1:e182. http://dx.doi.org/10.7717/peerj.182

Title: Surface model for pedal phalanges of *Parasaurolophus* sp. (Hadrosauridae: Dinosauria)
Published URL: http://dx.doi.org/10.6084/m9.figshare.797520
File Name: RAM14000_Phalanges.zip

Description: This dataset includes 3D surface models for right pedal phalanges (III-2 and III-3) from RAM 14000, a hadrosaurid dinosaur referred to *Parasaurolophus* sp.

Taxon: *Parasaurolophus* sp. (Lambeosaurinae: Hadrosauridae: Ornithischia: Dinosauria)

Specimen: RAM 14000, Raymond M. Alf Museum of Paleontology, Claremont, California, USA. This catalog number comprises the articulated skull and skeleton of a single individual.

Locality and Horizon: Locality RAM V200921, Grand Staircase-Escalante National Monument, Garfield County, Utah, USA; upper part of middle unit of the Kaiparowits Formation (late Campanian, Cretaceous). Detailed locality data are on file at the RAM and are available to qualified investigators upon request.

File Information: The original point cloud was captured using a NextEngine 3D color laser scanner (NextEngine, Inc., Santa Monica, California), in September 2013. A total of 16 individual scans (2 sets of 8 divisions each, with 45 degree rotation of the specimen between scans) were acquired at a resolution of 6,200 points/cm^2. The individual scans were stitched together in ScanStudio HD Pro 1.3.2 (NextEngine, Inc., Santa Monica, California) and fused into a single watertight mesh (0.000254 cm tolerance, 10 pixel texture blending, 0.5 resolution ratio). The resulting mesh had 82.072 points and 127,256 triangles, and is scaled so that 1 unit equals 1 mm. Data were exported in PLY, OBJ, and STL formats, with a total size of 25.3 Mb; these files were compressed into a single ZIP archive, at 8.57 Mb.

Additional Information: RAM 14000 was collected under United States Department of the Interior Bureau of Land Management Paleontological Resources Use Permit (surface collection permit UT06-001S and excavation permit UT10-006E-Gs). The specimen is accessioned into the permanent collection of the Raymond M. Alf Museum of Paleontology (RAM), Claremont, California, USA.

The Alf Museum kindly requests that any publications resulting from these data acknowledge the museum and cite this dataset as well as the original Farke et al. publication, and that a link to or copy of any resulting publications be forwarded to the museum director and/or curator.

Publication Citation: Farke, A. A., D. J. Chok, A. Herrero, B. Scolieri, and S. Werning. 2013. Ontogeny in the tube-crested dinosaur *Parasaurolophus* (Hadrosauridae) and heterochrony in hadrosaurids. *PeerJ* 1:e182. http://dx.doi.org/10.7717/peerj.182

Title: Surface model for pedal ungual of *Parasaurolophus* sp. (Hadrosauridae: Dinosauria)
Published URL: http://dx.doi.org/10.6084/m9.figshare.797515
File Name: RAM14000_Ungual.zip

Description: This dataset includes 3D surface models for a right pedal ungual (III-4) from RAM 14000, a hadrosaurid dinosaur referred to *Parasaurolophus* sp.

Taxon: *Parasaurolophus* sp. (Lambeosaurinae: Hadrosauridae: Ornithischia: Dinosauria)

Specimen: RAM 14000, Raymond M. Alf Museum of Paleontology, Claremont, California, USA. This catalog number comprises the articulated skull and skeleton of a single individual.

Locality and Horizon: Locality RAM V200921, Grand Staircase-Escalante National Monument, Garfield County, Utah, USA; upper part of middle unit of the Kaiparowits Formation (late Campanian, Cretaceous). Detailed locality data are on file at the RAM and are available to qualified investigators upon request.

File Information: The original point cloud was captured using a NextEngine 3D color laser scanner (NextEngine, Inc., Santa Monica, California), in September 2013. A total of 14 individual scans (2 sets, the first comprising 6 scans with approximately 60 degree rotation between each, and the second comprising 8 scans with approximately 45 degree rotation between each) were acquired at a resolution of 6,200 points/cm^2. The individual scans were stitched together in ScanStudio HD Pro 1.3.2 (NextEngine, Inc., Santa Monica, California) and fused into a single watertight mesh (0.000254 cm tolerance, 10 pixel texture blending, 0.9 resolution ratio). The resulting mesh had 77,820 points and 124,936 triangles, and is scaled so that 1 unit equals 1 mm. Data were exported in PLY, OBJ, and STL formats, with a total size of 24.1 Mb; these files were compressed into a single ZIP archive, at 7.93 Mb.

Additional Information: RAM 14000 was collected under United States Department of the Interior Bureau of Land Management Paleontological Resources Use Permit (surface collection permit UT06-001S and excavation permit UT10-006E-Gs). The specimen is accessioned into the permanent collection of the Raymond M. Alf Museum of Paleontology (RAM), Claremont, California, USA.

The Alf Museum kindly requests that any publications resulting from these data acknowledge the museum and cite this dataset as well as the original Farke et al. publication, and that a link to or copy of any resulting publications be forwarded to the museum director and/or curator.

Publication Citation: Farke, A. A., D. J. Chok, A. Herrero, B. Scolieri, and S. Werning. 2013. Ontogeny in the tube-crested dinosaur *Parasaurolophus* (Hadrosauridae) and heterochrony in hadrosaurids. *PeerJ* 1:e182. http://dx.doi.org/10.7717/peerj.182
