# Supplementary figures and images for: Ontogeny in the tube-crested dinosaur Parasaurolophus (Hadrosauridae) and heterochrony in hadrosaurids
Source: PeerJ. 2013 Oct 22;1:e182. doi: 10.7717/peerj.182 (PMC3807589; doi:10.7717/peerj.182)

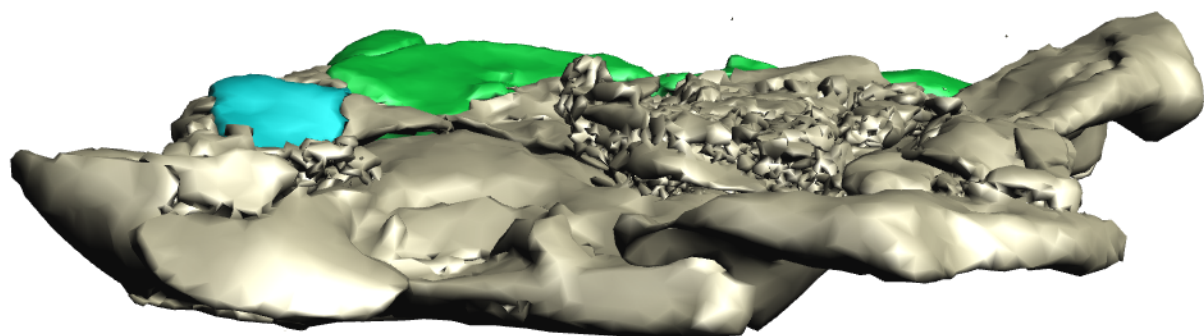

Supplement: Figure S1 — This digital reconstruction is based on CT scan data, with the skull is shown in white, the nasal passages in green, and the endocranial cavity in blue. A high-resolution two-dimensional image is included in Fig. 9, and raw data are available from Figshare (Table 1, Article S1). [file peerj-01-182-s002.pdf]

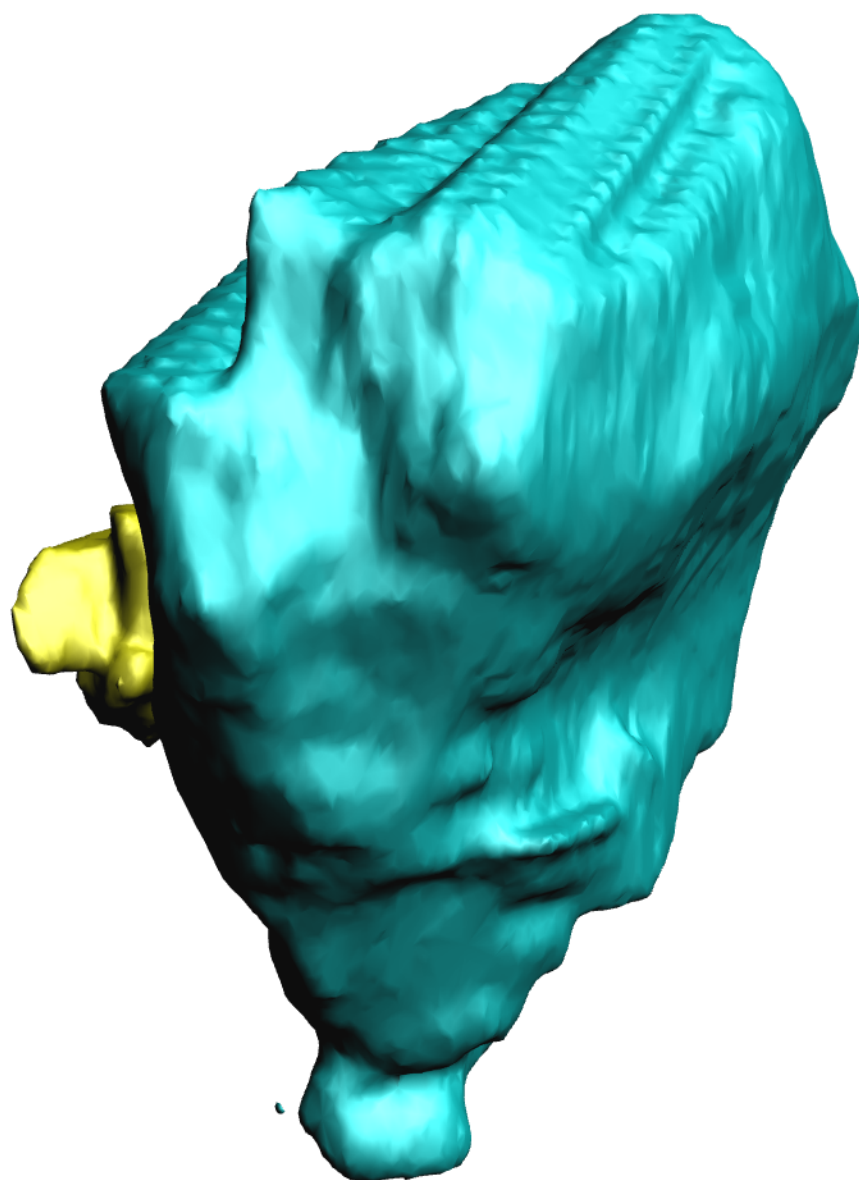

Supplement: Figure S2 — This digital reconstruction is based on CT scan data, with the endocranial cavity in blue and the endosseous labyrinth in yellow. A high-resolution two-dimensional image is included in Fig. 15, and raw data are available from Figshare (Table 1, Article S1). [file peerj-01-182-s003.pdf]
